# Association of tranexamic acid use and autologous predonation with blood loss and transfusion outcomes after periacetabular osteotomy: An arm‐based multilevel meta analysis

**DOI:** 10.1002/jeo2.70867

**Published:** 2026-07-28

**Authors:** Nikolai Ramadanov, Jonathan Lettner, Roland Becker, Robert Prill, Marko Ostojic, Sufian S. Ahmad

**Affiliations:** ^1^ Center of Orthopaedics and Traumatology, Brandenburg Medical School University Hospital Brandenburg an der Havel Brandenburg an der Havel Germany; ^2^ Faculty of Health Science Brandenburg Brandenburg Medical School Theodor Fontane Brandenburg an der Havel Germany; ^3^ Sports Traumatology Division, Traumatology Department “Draškovićeva” University Hospital “Sisters of Mercy” Zagreb Croatia; ^4^ Osteon Orthopaedics and Sports Medicine Clinic Mostar Bosnia and Herzegovina; ^5^ Department of Orthopaedic Surgery Hannover Medical School Hannover Germany

**Keywords:** blood loss, blood transfusion, hip dysplasia, meta‐analysis, periacetabular osteotomy, perioperative blood management

## Abstract

**Purpose:**

To evaluate the association between tranexamic acid (TXA) use, autologous predonation and perioperative blood management outcomes after periacetabular osteotomy (PAO) using an arm‐based multilevel meta‐analytic framework.

**Methods:**

A systematic search of PubMed, Embase, Epistemonikos and the Cochrane Central Register of Controlled Trials (CENTRAL) was performed up to 30 March 2026. Prospective and retrospective primary clinical studies reporting perioperative blood management outcomes after PAO were included. Comparisons between TXA and non‐TXA arms, as well as predonation and no‐predonation arms, were performed using arm‐based multilevel random‐effects meta‐analysis.

**Results:**

Forty‐three studies comprising 4315 patients and 4674 hips were included. TXA use was associated with lower estimated blood loss (mean difference [MD] −185.6 mL, 95% confidence interval [CI]: −341.1 to −30.1), lower intraoperative blood loss (MD: −299.4 mL, 95% CI: −392.2 to −206.6), fewer transfused units (MD: −0.67, 95% CI: −0.89 to −0.45) and a lower transfusion rate (OR: 0.14, 95% CI: 0.056–0.338). No significant differences were observed for haemoglobin‐related outcomes, complications, reoperations or conversion to total hip arthroplasty. Autologous predonation was not associated with reductions in blood loss or transfusion‐related outcomes and was associated with lower postoperative haemoglobin levels (MD: −5.5 g/L, 95% CI: −9.0 to −1.9). The certainty of evidence ranged from low to very low, and substantial heterogeneity was observed across most analyses.

**Conclusion:**

TXA use was associated with reduced blood loss and transfusion requirements after PAO, whereas autologous predonation was not associated with measurable clinical benefit. Given the substantial heterogeneity and low certainty of evidence, these findings should be interpreted cautiously.

**Level of Evidence:**

Level III, systematic review and meta‐analysis of predominantly retrospective cohort studies.

## Graphical abstract

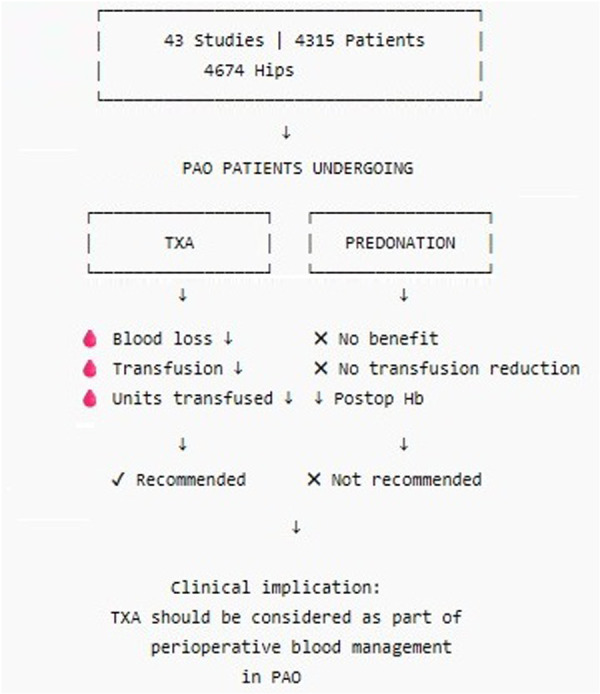

**Abbreviations**
BDDH: borderline developmental dysplasia of the hip
BMI: body mass index
CENTRAL: Cochrane Central Register of Controlled Trials
CI: confidence interval
DDH: developmental dysplasia of the hip
GRADE: grading of recommendations assessment, development and evaluation
HAS: hip arthroscopy
*I* ^2^
: Higgins’ *I*‐squared statistic
MD: mean difference
NR: not reported
OR: odds ratio
PAO: periacetabular osteotomy
PRISMA: preferred reporting items for systematic reviews and meta‐analyses
PROSPERO: international prospective register of systematic reviews
RCT: randomized controlled trial
REML: restricted maximum likelihood
ROBINS‐I: risk of bias in non‐randomised studies of interventions
RoB 2: Risk of bias 2 (tool for randomised trials)
SD: standard deviation
THA: total hip arthroplasty
TXA: tranexamic acid

## INTRODUCTION

Periacetabular osteotomy (PAO) is an established joint‐preserving procedure for the treatment of symptomatic acetabular dysplasia and has demonstrated favourable mid‐ to long‐term outcomes in appropriately selected patients [118]. Despite these encouraging results, PAO remains a technically demanding procedure that is consistently associated with substantial perioperative blood loss and a considerable rate of blood transfusion.

Effective blood management is therefore a key aspect of perioperative care in PAO. Several systematic reviews and meta‐analyses have investigated the role of antifibrinolytic agents in this setting. Early evidence suggested that antifibrinolytics are associated with reduced total blood loss, haemoglobin decline and transfusion rates without increasing complication rates [120]. Subsequent analyses focusing on tranexamic acid (TXA) confirmed these findings, demonstrating significant reductions in blood loss and, in part, transfusion requirements in patients undergoing PAO [94, 122]. However, these studies were limited by small sample sizes, inclusion of heterogeneous procedures such as high tibial osteotomy, and a primary focus on pairwise comparisons rather than arm‐based analyses.

Beyond pharmacological strategies, other aspects of perioperative management in PAO have been addressed in recent systematic reviews, including surgical technique, computer‐assisted modalities and patient selection [2, 75, 110]. These studies highlight substantial heterogeneity in surgical practice, patient populations and perioperative protocols [27], all of which may influence perioperative outcomes. In parallel, recent studies have expanded the evidence base in PAO and related conditions such as developmental dysplasia of the hip (DDH) and borderline DDH (BDDH), providing more comprehensive quantitative analyses of surgical outcomes and patient‐specific factors [1, 46, 89, 90]. However, despite these advances, perioperative blood management strategies remain insufficiently addressed in this context. In contrast, strategies such as autologous predonation have been inconsistently reported and remain insufficiently evaluated in the setting of modern blood management approaches.

To date, there is no comprehensive synthesis of the available literature using an arm‐based analytical framework to evaluate the association between TXA use, autologous predonation and blood‐related outcomes in PAO. In particular, the associations of these strategies with perioperative blood management outcomes across heterogeneous study populations remain unclear.

Therefore, the aim of this study was to perform an arm‐based multilevel meta‐analysis to assess the association of TXA use and autologous predonation with perioperative blood loss, transfusion requirements and clinical outcomes in PAO. It was hypothesised that TXA use would be associated with reduced blood loss and transfusion rates, whereas autologous predonation would not be associated with additional clinical benefit.

## METHODS

### Protocol registration and reporting standards

This systematic review was prospectively registered in the International prospective register of systematic reviews (PROSPERO) on 24 March 2026 (CRD420261350120). The study was conducted in accordance with the preferred reporting items for systematic reviews and meta‐analyses (PRISMA) 2020 statement [80]. The completed PRISMA checklist is provided in the Supplementary Appendix (Table S1).

### Search strategy

A comprehensive literature search was performed in four electronic databases: PubMed, Embase, Epistemonikos and the Cochrane Central Register of Controlled Trials (CENTRAL). The final search was conducted on 30 March 2026. The search strategy combined controlled vocabulary and free‐text terms related to PAO and DDH, using the following Boolean structure: ((periacetabular osteotomy OR PAO OR Bernese) AND (dysplasia OR DDH OR borderline dysplasia OR BDDH)). Search syntax was adapted as required for each database. No restrictions on publication date or language were applied.

### Study screening and eligibility

Two reviewers (N.R. and J.L.) independently screened all records in a two‐step process: (1) title and abstract screening and (2) full‐text assessment. Discrepancies were resolved through discussion until consensus was achieved. Interreviewer agreement was quantified using Cohen's kappa (*κ*). Eligible studies included prospective or retrospective primary clinical studies investigating PAO that reported data relevant to perioperative blood management. Both comparative studies and single‐arm cohorts were considered eligible, provided that they reported at least one blood management‐related parameter. Exclusion criteria comprised reviews, editorials, case reports, animal studies and grey literature.

### Research items and outcome measures

The primary exposures of interest were: (1) administration of TXA (intravenous, topical or combined) [32], and (2) use of autologous blood predonation strategies [33]. Continuous outcomes included estimated blood loss, intraoperative blood loss, postoperative haemoglobin levels, haemoglobin decrease, number of transfused units, operation time and length of hospital stay. Dichotomous outcomes included transfusion rate, complication rate, reoperation rate and conversion to total hip arthroplasty (THA).

### Data extraction

Data extraction was performed independently by two reviewers (N.R. and J.L.) using a standardised, predefined data collection form. Extracted variables included study characteristics (design, sample size), patient demographics, perioperative parameters and follow‐up duration. Information on TXA use and autologous predonation was extracted where available and evaluated as study‐arm characteristics within the arm‐based analyses. For missing data, a predefined hierarchical approach was applied: (1) contacting corresponding authors; (2) estimating standard deviations (SDs) from reported ranges using the formula: (maximum−minimum)/4; [115] (3) imputing SDs where necessary using established methods [115]. For studies reporting multiple treatment arms unrelated to the predefined blood management interventions of interest (TXA use and autologous predonation), the respective arms were combined into a single pooled cohort to avoid double‐counting and permit inclusion in the overall arm‐based synthesis.

### Quality assessment

Risk of bias was assessed independently by both reviewers using the ROBINS‐I tool for nonrandomised studies [102] and the RoB 2 tool for randomised controlled trials [103]. Disagreements were resolved through consensus. The certainty of evidence for each outcome was evaluated using the GRADE approach [37]. Potential small‐study effects were assessed visually using funnel plots.

### Statistical analysis

All statistical analyses were performed using R (R Foundation for Statistical Computing), employing the meta and metafor packages. Given that many included studies did not provide direct comparative analyses, an arm‐based meta‐analytic framework was applied, treating individual treatment arms as the unit of analysis rather than pairwise comparisons. For continuous outcomes, pooled mean estimates with 95% confidence intervals (CIs) were calculated using random‐effects models with restricted maximum likelihood estimation. For dichotomous outcomes, pooled proportions with 95% CIs were derived after appropriate transformation. Comparisons between TXA and non‐TXA groups, as well as between predonation and no‐predonation groups, were conducted using subgroup difference tests. To account for the inclusion of multiple treatment arms from the same study, a multilevel meta‐analytic model was implemented, thereby adjusting for within‐study dependency of effect sizes [52, 76]. Between‐study heterogeneity was quantified using the Higgins *I* ^2^‐statistic, with thresholds of <25%, 25%–75% and >75% indicating low, moderate and high heterogeneity, respectively [41]. Given the anticipated heterogeneity and the limited number of studies for certain outcomes, the Hartung–Knapp adjustment was applied to provide more robust CI estimates [39, 88]. A two‐sided *p*‐value < 0.05 was considered statistically significant. A predefined sensitivity analysis was additionally performed, including only studies with directly reported standard deviations, to evaluate the robustness of the primary findings and the potential influence of SD imputation.

## RESULTS

### Search results

**Figure 1:**
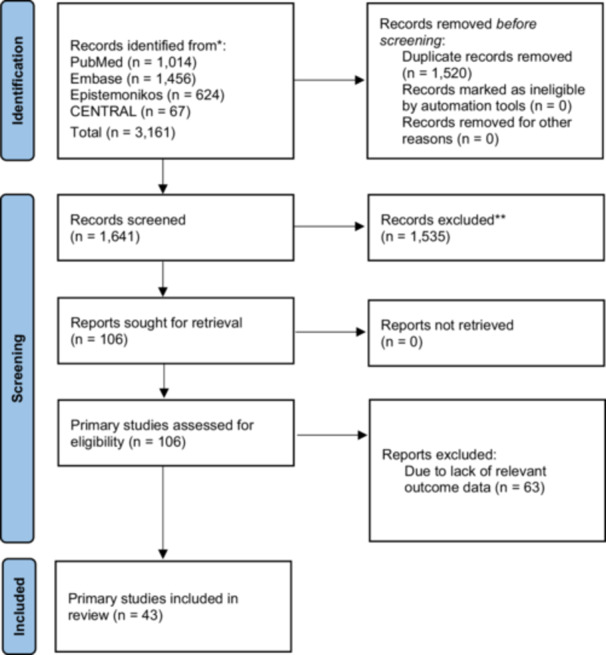
PRISMA 2020 flow diagram illustrating study selection process. CENTRAL, Cochrane Central Register of Controlled Trials; PRISMA, preferred reporting items for systematic reviews and meta‐analyses.

A total of 1014 records were identified through PubMed, 1456 through Embase, 624 through Epistemonikos and 67 through the CENTRAL. After removal of 1520 duplicates, 1641 records remained for title and abstract screening. Screening was performed independently by two reviewers with excellent interreviewer agreement (*κ* = 0.98). A total of 106 full‐text articles [3, 4, 5, 6, 7, 8, 9, 10, 11, 12, 13, 14, 15, 16, 17, 18, 19, 20, 21, 22, 23, 24, 25, 26, 28, 29, 30, 31, 34, 35, 36, 38, 40, 42, 43, 44, 45, 47, 48, 49, 50, 51, 53, 70, 71, 72, 73, 74, 75, 77, 78, 79, 81, 82, 83, 84, 85, 86, 91, 92, 93, 94, 95, 96, 97, 98, 99, 100, 101, 104, 105, 106, 107, 108, 109, 110, 111, 112, 113, 114, 116, 117, 118, 119, 120, 121, 122, 123, 124, 125, 126] were assessed for eligibility, with complete agreement between reviewers (*κ* = 1.0). Of these, 63 studies [3, 6, 7, 8, 10, 11, 12, 14, 17, 19, 20, 21, 22, 23, 24, 25, 26, 28, 29, 30, 31, 34, 35, 36, 40, 42, 43, 44, 45, 48, 50, 51, 54, 56, 57, 58, 59, 68, 69, 72, 73, 74, 75, 77, 78, 79, 80, 81, 86, 91, 92, 93, 94, 104, 105, 106, 107, 110, 113, 116, 117, 119, 120, 121] were excluded due to lack of relevant outcome data. Ultimately, 43 primary studies [4, 5, 9, 13, 15, 16, 18, 38, 47, 49, 53, 55, 60, 61, 62, 63, 64, 65, 66, 67, 70, 71, 82, 83, 84, 85, 95, 96, 97, 98, 99, 100, 101, 108, 109, 111, 112, 114, 118, 123, 124, 125, 126] were included in the final meta‐analysis (Figure 1).

### Study characteristics

A total of 43 studies [4, 5, 9, 13, 15, 16, 18, 38, 47, 49, 53, 55, 60, 61, 62, 63, 64, 65, 66, 67, 70, 71, 82, 83, 84, 85, 95, 96, 97, 98, 99, 100, 101, 108, 109, 111, 112, 114, 118, 123, 124, 125, 126] were included, comprising retrospective cohort studies (*n* = 10) [4, 5, 9, 15, 55, 60, 70, 99, 100, 124] and comparative studies (*n* = 14) [13, 49, 63, 64, 65, 66, 67, 71, 82, 96, 112, 114, 118, 123], case series (*n* = 16) [16, 18, 38, 62, 83, 84, 85, 95, 97, 98, 101, 108, 109, 111, 125, 126] and a limited number of prospective cohorts (*n* = 2) [47, 53] and randomised trials [61]. The majority of studies were retrospective in design (*n* = 38) [4, 5, 9, 13, 15, 16, 18, 38, 49, 55, 60, 62, 63, 64, 66, 70, 71, 82, 83, 84, 85, 95, 96, 97, 98, 99, 100, 101, 108, 109, 111, 112, 114, 118, 123, 124, 125, 126] and classified as level III (*n* = 21) [4, 5, 9, 13, 15, 49, 55, 60, 63, 66, 67, 70, 71, 82, 96, 99, 100, 112, 114, 118, 123] or IV (*n* = 17) [16, 18, 38, 62, 83, 84, 85, 95, 97, 98, 101, 108, 109, 111, 124, 125, 126] evidence, with only few level I–II studies (*n* = 5) [47, 53, 61, 64, 65] identified. The included studies were published between 1999 and 2025 and originated from multiple geographic regions, including North America (*n* = 17) [15, 18, 53, 55, 61, 66, 67, 70, 82, 85, 95, 99, 100, 118, 124, 126], Europe (*n* = 13) [4, 9, 13, 16, 38, 47, 84, 98, 101, 111, 112, 114] and Asia (*n* = 11) [5, 49, 62, 63, 64, 65, 96, 97, 108, 123, 125], with the majority conducted in the United States (*n* = 16) [15, 18, 53, 55, 61, 66, 70, 82, 83, 85, 95, 99, 100, 118, 124, 126], Switzerland (*n* = 4) [4, 60, 98, 101], Germany (*n* = 3) [13, 38, 114] and China (*n* = 7) [62, 63, 64, 65, 96, 108, 125] (Table 1). Information on whether standard deviations were reported or imputed for each included study is provided in Table S2.

**Table 1:** Study characteristics and patient demographics of included studies.

Author	Year	Country	Journal	Study design	Level of evidence	Study period	Follow‐up (years ± SD; range)	Technique	Surgical approach	Concomitant procedures	Patients/hips (*N*)	Female sex (*N*)	Mean age (year ± SD; range)	BMI (kg/m^2 ^± SD; range)	Remarks
Albers CE	2013	Switzerland	Clinical Orthopaedics and Related Research	Retrospective cohort study	3	1984–1987; 1997–2000	11.0 ± 1.0;10.0–14.0	Bernese	Modified Smith‐Petersen	Osteochondroplasty (57%), Labrum procedures (49%), intertrochanteric osteotomy (10%–23%)	147/165	94	29.0	23.0 ± 4.0; 19.0–33.0	NR
Amano T	2014	Japan	Hip International	Retrospective cohort study	3	1989–2007	13.3; 5.0–21.0	Eccentric rotational acetabular osteotomy	NR	NR	108/108	72	35.9	21.8 ± 2.2	NR
Atwal NS	2008	UK	Hip International	Retrospective cohort study	3	1996–2005	NR	Bernese	Modified Stoppa approach	NR	107/122	109	23.6; 18.7–28.6	NR	NR
Bernstein P	2007	Germany	The Open Orthopaedics Journal	Retrospective comparative study	3	NR	1.6; 0.5–2.4	Bernese	Modified Smith‐Petersen	Labrum/FAI: 2; Femoral osteotomy: 4	23/23	15	27.0; 16.0–40.0	NR	NR
Bryan AJ	2016	USA	Orthopaedics	Retrospective cohort	3	2003–2014	1.3–5.8	Bernese	Modified Smith‐Petersen	NR	137/150	115	26.4 ± 8.7	26.0 ± 5.3	NR
								Bernese	Modified Smith‐Petersen	NR	68/75	58	24.8 ± 8.2	26.0 ± 6.3	TXA
								Bernese	Modified Smith‐Petersen	NR	69/75	57	28.0 ± 9.2	26.0 ± 4.3	nonTXA
Burke NG	2011	Ireland	Acta Orthopaedica Belgica	Retrospective case series	4	1998–2005	4.9; 1.3–8.0	Bernese	Modified Smith‐Petersen	NR	79/85	72	22.9; 14.0–41.0	NR	NR
Clohisy JC	2005	USA	JBJS Am	Retrospective case series	4	1994–2001	4.2; 1.9–8.1	Bernese	Modified Smith‐Petersen	6 proximal femoral osteotomies	13/16	1	17.6; 13.0–31.8	NR	NR
Haertlé M	2024	Germany	Bone & Joint Journal	Retrospective case series	4	2022–2023	NR	Bernese	NR	Offset correction: 27, Femoral osteotomy: 3, Implant removal: 3, Surgical dislocation: 1	106/118	90	23.4 ± 7.5	24.9 ± 4.6; 17.3–37.8	NR
Khan OH	2017	UK	Bone & Joint Journal	Prospective cohort study	2	2010–2013	2.8; 1.2–4.5	Bernese	Modified Smith‐Petersen	NR	151/166	136	32.0; 15.0–56.0	NR	NR
Kim HT	2009	South Korea	JBJS Br	Retrospective comparative study	3	2001–2008	NR	Bernese	Mixed (single anterior + dual AP)	NR	23/26	19	19.4; 11.3–38.0	NR	NR
Kraeulter MJ	2018	USA	Journal of Hip Preservation Surgery	Prospective cohort study	2	NR	2.0	Bernese	NR	Staged hip arthroscopy (alle Patienten)	124/145	118	30.1 ± 8.9; 15.0–50.0	23.8 ± 5.1; 16.3–44.9	NR
Lee CB	2013	USA	Hip International	Retrospective cohort study	3	2009–2011	NR	Bernese	Abductor‐sparing	NR	141/169	125	25.6 ± 9.7	25.2 ± 4.8	NR
Lerch TD	2017	Switzerland	Clinical Orthopaedics and Related Research	Retrospective cohort study	3	1984–1987	29.0; 27.0–32.0	Bernese	NR	Concomitant intertrochanteric osteotomy in 16 hips	63/75	58	29.0 ± 12; 13.0–56.0	22.0 ± 3.0; 16.0–28.0	NR
Levack AE	2020	USA	Bone & Joint Journal	RCT	1	2014–2018	0.12	Bernese	Modified Smith‐Petersen	HAS: 23	81/81	78	25.9 ± 7.4; 14.0–46.0	23.5 ± 4.0; 16.3–36.8	NR
								Bernese	Modified Smith‐Petersen	HAS: 13	40/40	38	25.1 ± 7.2; 15.0–42.0	23.9 ± 4.5; 16.9–36.8	TXA
								Bernese	Modified Smith‐Petersen	HAS: 10	41/41	40	26.8 ± 7.7; 14.0–46.0	23.1 ± 3.5; 16.3–31.8	nonTXA
Li C	2022	China	BMC Surgery	Retrospective case series	4	2016–2019	2.93 ± 0.88; 1.5–4.7	Bernese	modified Smith‐Petersen/Bikini + posterolateral assist incision	NR	58/65	52	28.1 ± 8.4; 11.0–49.0	22.0 ± 3.0; 17.4–30.4	NR
Luo D	2016	China	Therapeutics and Clinical Risk Management	Retrospective comparative study	3	2010–2011	NR	Bernese	improved ilioinguinal approach; modified Smith‐Petersen approach; two‐incision Smith‐Petersen approach	Arthroscopy, femoral osteotomy, labrum repair	95/101	81	11.0–45.0	NR	NR
Luo R	2021	China	Journal of Orthopaedic Surgery and Research	Retrospective comparative study	1	2014–2019	1.0	Bernese	modified Smith‐Petersen/ilioinguinal	NR	61/66	39	26.8 ± 8.3; 16.0–45.0	20.4 ± 1.2	NR
Ma S	2022	China	The International Journal of Artificial Organs	Randomised comparative study	2	2016–2020	0.5	Bernese	Modified Smith‐Petersen	NR	22/22	18	28.0; 16.0–36.0	NR	NR
Markhardt BK	2021	USA	Journal of Hip Preservation Surgery	Retrospective comparative study	3	2017–2020	NR	Bernese	Anterior (bikini, rectus sparing)	NR	26/28	24	29.4 ± 8.6	25.8 ± 4.2	NR
Marshall A	2025	Canada	The Journal of Arthroplasty	Retrospective comparative study	3	2015–2021	0.25	Bernese	Smith‐Petersen/bikini	Hip arthroscopy: 27; Osteochondroplasty: 7; Subspine decompression: 18;	94/94	70	28.0; 16.0–46.0	25.8; 18.9–37.1	NR
McLawhorn AS	2016	USA	The Journal of Arthroplasty	Retrospective cohort study	3	2011–2014	NR	Bernese	Modified Smith‐Petersen	HAS: 22	93/93	89	24.2 ± 8.0	22.9 ± 3.4	NR
Peters CL	2006	USA	JBJS	Retrospective case series	4	1997–2003	3.89; 2.5–7.3	Bernese	Modified Smith‐Petersen	Femoral osteotomy *n* = 13; arthrotomy n = 49; osteochondroplasty *n* = 35; labral resection *n* = 11	73/83	55	28.0; 15.0–47.0	28.5; 17.1–33.9	NR
Peters CL	2015	USA	Clinical Orthopaedics and Related Research	Retrospective comparative study	3	2009–2013	NR	Bernese	Rectus takedown + rectus sparing (combined)	NR	75/75	55	24.0	23.5	NR
Pogliacomi F	2005	Italy/Sweden	Acta Orthopaedica	Retrospective case series	4	1994–2001	4.1; 1.5–8.0	Bernese	modified Smith‐Petersen/ilioinguinal	Us femoral osteotomy: 1; capsulotomy: 2;	32/36	31	35.0; 15.0–55.0	NR	NR
Pulido LF	2008	USA	Journal of Surgical Orthopaedic Advances	Retrospective case series	4	1996–2003	NR	Bernese	Modified Smith‐Petersen	NR	107/108	84	30.0; 12.0–49.0	23.6	NR
Sabbag CM	2019	USA	The American Journal of Sports Medicine	Prospectove case series	4	2007–2016	3.0; 1.0–8.0	Bernese	NR	HAS	240/248	207	26.6 ± 9.2; 12.0–53.0	NR	NR
Shang JJ	2020	China	Orthopaedic Surgery	Retrospective comparative study	3	2010–2018	NR	Bernese	NR	Proximal femoral osteotomy	307/307	307	28.7 ± 8.4	21.9 ± 3.1	NR
											74/74	74	28.5 ± 7.6	21.9 ± 3.4	nonTXA
											233/233	233	28.8 ± 8.6	21.9 ± 2.9	TXA
Shon HC	2023	South Korea	Archives of Orthopaedic and Trauma Surgery	Retrospective case series	4	1997–2005	11.5; 8.0–16.0	Bernese	Smith‐Petersen + Kocher‐Langenbeck (dual approach)		49/53	46	39.9; 13.0–62.0	NR	NR
Siebenrock KA	1999	Switzerland	Clinical Orthopaedics and Related Research	Retrospective case series	4	1991–1995	11.3	Bernese	Anterior (Smith‐Petersen/ilioinguinal‐type)	Additional acetabular procedures: 13; femoral (abduction) osteotomy: 7	63/75	NR	29.0; 13.0–56.0	NR	NR
Sierra RJ	2017	USA	Journal of Hip Surgery	Retrospective cohort study	3	1996–2009	10.0; 24.0–236.0	Bernese	Anterior‐based	HAS: 1	268/299	123	31.0; 12.0–56.0	NR	NR
Stambough JB	2014	USA	Clinical Orthopaedics and Related Research	Retrospective multicenter cohort study	3	2008–2012	2.5 ± 1.1; 2.0–5.1	Bernese	Anterior‐based	osteochondroplasty: 29; arthroscopy: 15; labral repair: 6; intertrochanteric osteotomy: 2; labral resection: 3;	78/78	70	19.9 ± 6.0; 9.0–35.4	24.0 ± 4.3; 11.7–43.0	NR
Steppacher SD	2008	Switzerland	Clinical Orthopaedics and Related Research	Retrospective case series	4	1984–2003	20.4 ± 1.1; 19.0–23.0	Bernese	Modified Smith‐Petersen	intertrochanteric osteotomy: 16	63/75	58	29.3 ± 11.6; 13.0–56.0	22.1 ± 3.1; 15.8–28.2	NR
Tang Y	2022	China	International Orthopaedics	Retrospective case series	4	2019–2021	2.0; 1.28–2.32	Modifizierte Bernese	Combined posterior + anterior mini‐incisions	NR	34/34	32	38.7; 25.0–54.0	21.4 ± 0.7	NR
Thawrani D	2010	USA	JBJS Am	Retrospective case series	4	NR	2.0	Bernese	Modified Smith‐Petersen (abductor‐sparing)	NR	76/83	62	15.6 ± 2.4; 11.0–21.0	23.9 ± 4.9	NR
Troelsen A (1)	2008	Denmark	Acta Orthopaedica	Retrospective comparative study	3	1998–2007	5.0; 1.0–9.2	Bernese	Ilioinguinal; minimally invasive (transsartorial)	NR	211/263	174	33.0	NR	NR
Troelsen A (2)	2008	Denmark	JBJS Am	Retrospective case series	4	2003–2005	4.3; 2.0–4.3	Bernese	Minimally invasive transsartorial	NR	91/94	76	37.2	NR	NR
van der Merwe M	2019	New Zealand	Journal of Hip Preservation Surgery	Retrospective comparative study	3	2016–2018	NR	Bernese	Modified Smith‐Petersen	No	58/58	44	24.7; 17.6–30.2	24.6; 22.1–28.3	NR
								Bernese	Modified Smith‐Petersen	No	40/40	33	24.7; 17.6–29.4	25.8; 23.4–28.3	Cell saver
								Bernese	Modified Smith‐Petersen	No	18/18	11	23.8; 17.9–30.2	23.5; 22.1–27.7	No cell saver
Wassilew GI	2015	Germany	The Bone & Joint Journal	Retrospective comparative study	3	NR	NR	Bernese	NR	No	96/96	84	29.6 ± 8.7	23.9 ± 4.4	NR
								Bernese	NR	No	48/48	42	27.4 ± 7.0	24.2 ± 4.7	TXA
								Bernese	NR	No	48/48	42	31.7 ± 10.1	23.5 ± 4.0	noTXA
Wingerter SA	2015	USA	Clinical Orthopaedics and Related Research	Retrospective comparative study	3	2011–2013	NR	Bernese	NR	HAS: 34	100/100	85	27.5; 14.0–49.0	23.5; 17.0–33.0	NR
								Bernese	NR	HAS: 23	50/50	44	27.0; 17.0–47.0	23	TXA
								Bernese	NR	HAS: 11	50/50	41	28.0; 13.0–49.0	24	noTXA
Yilmaz M	2022	Turkey	European Review for Medical and Pharmacological Sciences	Retrospective comparative study	3	2001–2015	7.53 ± 3.14; 6.0–15.0	Bernese	Modified Smith‐Petersen	NR	43/43	31	32.2 ± 8.4; 19.0–45.0	26.0 ± 2.6; 18.9–29.4	NR
Zaltz I	2014	USA	JBJS	Prospective case series	4	2007–2009	1.2; 0.7–2.9	Ganz	Smith‐Petersen	Femoral head–neck osteochondroplasty: 118; hip arthroscopy: 42;	205/205	143	25.4; 11.0–54.0	25.3; 11.7–46.6	NR
Zhu J	2013	China	International Orthopaedics	Retrospective case series	4	2001–2009	5.1; 2.0–10.0	Bernese	Modified Smith‐Petersen	osteochondroplasty: 11; varus osteotomy: 4 hips; labrum debridement: 3 hips;	36/41	34	39.5; 35.0–54.0	21.4; 18.2–26.6	NR
Ziran N	2018	USA	Journal of the American Academy of Orthopaedic Surgeons	Retrospective case series	4	1987–2014	11.2; 2.0–27.0	Bernese	Modified Smith‐Petersen	NR	258/302	215	32.8; 13.0–63.0	NR	NR

*Note*: Summary of included studies reporting study design, sample size, patient demographics (age, sex, BMI), surgical technique, concomitant procedures and follow‐up duration. Studies are presented with corresponding references [21, 22, 23, 24, 25, 26, 27, 28, 29, 30, 31, 32, 33, 34, 35, 36, 37, 38, 39, 40, 41, 42, 43, 44, 45, 46, 47, 48, 49, 50, 51, 52, 53, 54, 55, 56, 57, 58, 59, 60, 61, 62, 63]. Variability in surgical approach, patient characteristics and follow‐up reflects heterogeneity across cohorts.

Abbreviations: BMI, body mass index; HAS, hip arthroscopy; NR, not reported; RCT, randomized controlled trial; SD, standard deviation; TXA, tranexamic acid.

### Study population

Across all included studies [4, 5, 9, 13, 15, 16, 18, 38, 47, 49, 53, 55, 60, 61, 62, 63, 64, 65, 66, 67, 70, 71, 82, 83, 84, 85, 95, 96, 97, 98, 99, 100, 101, 108, 109, 111, 112, 114, 118, 123, 124, 125, 126], a total of 4315 patients and 4674 hips were analysed. Most studies reported a higher number of hips than patients, reflecting bilateral procedures in a subset of cases. The proportion of female patients was consistently high across studies, typically ranging between 60% and 90%, indicating a predominance of female patients undergoing PAO (Table 1).

### Patient characteristics

The mean age of patients ranged from approximately 15–40 years, with most studies reporting mean ages in the mid‐20s to early 30 s [4, 5, 15, 16, 55, 60, 62, 66, 67, 70, 71, 96, 97, 99, 100, 108, 114, 123, 124, 125, 126]. Standard deviations, where reported, indicated moderate variability within study populations [4, 15, 55, 60, 62, 66, 67, 70, 71, 96, 97, 108, 114, 123, 126]. Body mass index (BMI) was generally reported in the range of 21–26 kg/m^2^ [4, 5, 15, 55, 60, 62, 66, 67, 70, 71, 96, 108, 114, 123, 126], with relatively low dispersion, suggesting that most cohorts consisted of nonobese patients (Table 1).

### Surgical technique

The Bernese PAO was the predominant surgical technique used across all studies (*n* = 43) [4, 5, 9, 13, 15, 16, 18, 38, 47, 49, 53, 55, 60, 61, 62, 63, 64, 65, 66, 67, 70, 71, 82, 83, 84, 85, 95, 96, 97, 98, 99, 100, 101, 108, 109, 111, 112, 114, 118, 123, 124, 125, 126]. The most commonly reported surgical approach was the modified Smith‐Petersen approach (*n* = 20) [4, 9, 13, 15, 16, 47, 55, 61, 62, 70, 71, 82, 85, 98, 101, 108, 111, 114, 123, 125, 126], although variations such as bikini incisions [62, 66, 67], abductor‐sparing approaches [55, 109] and combined anterior–posterior approaches [49, 97] were also described (Table 1).

### Concomitant procedures

Concomitant procedures were frequently reported and included femoral osteochondroplasty [4, 18, 82, 95, 99, 108, 124], labral repair or debridement [4, 18, 49, 82, 95, 109], hip arthroscopy [95, 99, 124] and intertrochanteric or femoral osteotomy [4, 60, 82, 108]. The frequency and type of concomitant procedures varied substantially across studies, reflecting heterogeneity in surgical strategy and underlying patient pathology (Table 1).

### Follow‐up

Follow‐up duration varied widely between studies. Reported mean follow‐up ranged from short‐term (<1 year) [61, 65, 67] to long‐term (>20 years) [60, 101], with several studies reporting follow‐up periods exceeding 10 years [4, 5, 60, 97, 98, 99, 101, 126] (Table 1).

### Quality and publication bias assessment

A total of 42 nonrandomised studies were assessed using ROBINS‐I. Overall risk of bias was serious in 26 studies [4, 9, 13, 15, 38, 49, 55, 63, 64, 66, 67, 70, 71, 82, 85, 96, 99, 100, 108, 109, 112, 114, 118, 124, 125, 126] and critical in 13 studies [16, 18, 47, 53, 60, 62, 83, 84, 95, 97, 98, 101, 111], while only three studies showed moderate risk of bias [5, 65, 123]. Confounding was the dominant source of bias, with most studies rated as serious or critical. Other domains were largely rated as low to moderate risk. Only one randomised controlled trial was identified [61], which showed an overall low risk of bias (Table 2). Visual inspection of funnel plots suggested potential asymmetry for several outcomes; however, due to the limited number of included studies per analysis, formal assessment of publication bias was not considered reliable. Therefore, no definitive conclusions regarding the presence or absence of publication bias could be drawn (Figures S1–S23).

**Table 2:** Risk‐of‐bias assessment using ROBINS‐I and RoB 2.

Study/non‐RCTs	Confounding	Selection	Intervention classification	Deviations	Missing data	Outcome measurement	Reporting	Overall
Albers 2013	Serious	Moderate	Moderate	Low	Low	Low	Moderate	Serious
Amano 2014	Moderate	Moderate	Low	Low	Low	Low	Moderate	Moderate
Atwal 2008	Serious	Moderate	Low	Low	Low	Moderate	Moderate	Serious
Bernstein 2007	Serious	Moderate	Low	Low	Moderate	Moderate	Moderate	Serious
Bryan 2016	Serious	Moderate	Low	Low	Low	Moderate	Moderate	Serious
Burke 2011	Critical	Serious	Low	Low	Low	Moderate	Moderate	Critical
Clohisy 2005	Critical	Serious	Low	Low	Low	Moderate	Moderate	Critical
Haertlé 2024	Serious	Low	Low	Low	Low	Moderate	Low	Serious
Khan 2017	Critical	Moderate	Low	Low	Moderate	Moderate	Moderate	Critical
Kim 2009	Serious	Moderate	Low	Low	Low	Moderate	Moderate	Serious
Kraeutler 2018	Critical	Moderate	Low	Low	Low	Moderate	Moderate	Critical
Lee 2013	Serious	Low	Low	Low	Low	Moderate	Low	Serious
Lerch 2017	Critical	Serious	Low	Moderate	Moderate	Moderate	Moderate	Critical
Li 2022	Critical	Serious	Low	Low	Moderate	Moderate	Moderate	Critical
Luo D 2016	Serious	Moderate	Low	Low	Moderate	Moderate	Moderate	Serious
Luo R 2021	Serious	Moderate	Low	Low	Low	Moderate	Moderate	Serious
Ma S 2022	Moderate	Low	Low	Low	Low	Moderate	Moderate	Moderate
Markhardt BK 2021	Serious	Moderate	Low	Low	Low	Moderate	Moderate	Serious
Marshall A 2025	Serious	Moderate	Low	Low	Low	Moderate	Moderate	Serious
McLawhorn AS 2016	Serious	Moderate	Low	Low	Low	Moderate	Moderate	Serious
Peters CL 2015	Serious	Moderate	Low	Low	Low	Moderate	Moderate	Serious
Peters CL 2006	Critical	Serious	Low	Moderate	Moderate	Moderate	Moderate	Critical
Pogliacomi 2005	Critical	Serious	Low	Moderate	Moderate	Moderate	Moderate	Critical
Pulido LF 2008	Serious	Moderate	Low	Low	Low	Moderate	Moderate	Serious
Sabbag CM 2019	Critical	Moderate	Low	Low	Low	Moderate	Moderate	Critical
Shang JJ 2020	Serious	Moderate	Low	Moderate	Low	Moderate	Moderate	Serious
Shon HC 2023	Critical	Serious	Low	Low	Moderate	Moderate	Moderate	Critical
Siebenrock KA 1999	Critical	Serious	Low	Moderate	Moderate	Moderate	Moderate	Critical
Sierra RJ 2017	Serious	Moderate	Low	Low	Moderate	Moderate	Moderate	Serious
Stambough JB 2014	Serious	Moderate	Low	Low	Moderate	Low	Moderate	Serious
Steppacher SD 2008	Critical	Serious	Low	Low	Moderate	Moderate	Moderate	Critical
Tang Y 2022	Serious	Moderate	Low	Low	Low	Moderate	Moderate	Serious
Thawrani D 2010	Serious	Moderate	Low	Moderate	Low	Moderate	Moderate	Serious
Troelsen A et al. 2008 (1)	Serious	Moderate	Low	Moderate	Moderate	Moderate	Moderate	Serious
Troelsen A et al. 2008 (2)	Critical	Serious	Low	Low	Moderate	Moderate	Moderate	Critical
van der Merwe M et al. 2019	Serious	Moderate	Low	Low	Low	Moderate	Moderate	Serious
Wassilew GI et al. 2015	Serious	Moderate	Low	Low	Low	Moderate	Moderate	Serious
Wingerter SA et al. 2015	Serious	Moderate	Low	Low	Low	Moderate	Moderate	Serious
Yilmaz M et al. 2022	Moderate	Moderate	Low	Low	Low	Moderate	Moderate	Moderate
Zaltz I et al. 2014	Serious	Moderate	Low	Low	Moderate	Low	Low	Serious
Zhu J et al. 2013	Serious	Moderate	Low	Low	Moderate	Moderate	Moderate	Serious
Ziran N et al. 2019	Serious	Serious	Low	Low	Serious	Moderate	Moderate	Serious

Study/RCT	Randomisation	Deviations	Missing data	Outcome measurement	Reporting	Overall
Levack 2020	Low	Low	Low	Low	Some concerns	Low

*Note*: Risk‐of‐bias assessment for included studies using the ROBINS‐I tool for nonrandomised studies and the RoB 2 tool for randomised trials. Studies are categorised as low, moderate, serious or critical risk of bias across domains, with confounding identified as the predominant source of bias.

Abbreviations: RCT, randomized controlled trial; RoB 2, Risk of Bias 2 (tool for randomised trials); ROBINS‐I, rsk of bias in nonrandomised studies of interventions.

Certainty of evidence was downgraded primarily due to risk of bias (nonrandomised studies), substantial heterogeneity and imprecision. The overall certainty of evidence was low to very low across most outcomes according to the GRADE framework (Tables 3 and 4). For TXA, evidence was rated as low for transfusion rate and haemoglobin‐related outcomes, but very low for blood loss parameters due to serious risk of bias and extreme heterogeneity (Table 3). For autologous predonation, the certainty of evidence was consistently very low across outcomes, primarily due to limited sample sizes, high heterogeneity and risk of bias (Table 4).

**Table 3:** Summary of findings (GRADE): Tranexamic acid (TXA) versus no TXA in periacetabular osteotomy.

Outcome	No intervention (non‐TXA/no predonation)	Intervention effect	Relative effect (95% CI)	No. of studies (*k*)	Certainty of evidence (GRADE)	Comments
TXA versus no TXA						
Estimated blood loss	944 mL	↓ −185.6 mL	MD: −185.6 (−341.1 to −30.1)	21	⊕◯◯◯ Very low	High heterogeneity (*I* ^2^ > 98%)
Intraoperative blood loss	1139 mL	↓ −299.4 mL	MD: −299.4 (−392.2 to −206.6)	27	⊕◯◯◯ Very low	Consistent direction, but very high heterogeneity
Units transfused	2.06 units	↓ −0.67 units	MD: −0.67 (−0.89 to −0.45)	12	⊕◯◯◯ Very low	Sparse TXA data (*k* = 3)
Transfusion rate	33.0%	↓ to 6.2%	OR: 0.14 (0.056–0.338)	21	⊕⊕◯◯ Low	Clinically relevant reduction
Preoperative haemoglobin	131.8 g/L	No difference	MD: +1.5 (−0.4 to 3.4)	14	⊕⊕◯◯ Low	No effect
Postoperative haemoglobin	98.2 g/L	No difference	MD: +2.5 (−1.2 to 6.1)	11	⊕⊕◯◯ Low	No effect
Haemoglobin drop	31.3 g/L	No difference	MD: −0.1 (−4.2 to 3.9)	14	⊕⊕◯◯ Low	No effect
Complications	8.5%	No difference	OR: 0.86 (0.32–2.35)	33	⊕◯◯◯ Very low	Imprecise
Reoperations	3.9%	No difference	OR: 0.42 (0.041–4.39)	14	⊕◯◯◯ Very low	Very wide CI
THA conversion	5.4%	No difference	OR: 0.16 (0.015–1.67)	21	⊕◯◯◯ Very low	Sparse data

*Note*: Summary of effect estimates derived from arm‐based multilevel random‐effects meta‐analysis. Continuous outcomes are reported as mean differences (MD) and dichotomous outcomes as odds ratios (OR), each with 95% confidence intervals (CI). Certainty of evidence was assessed using the GRADE framework and was downgraded primarily due to risk of bias, substantial heterogeneity and imprecision.

Abbreviation: GRADE, grading of recommendations assessment, development and evaluation.

**Table 4:** Summary of findings (GRADE): Autologous predonation versus no predonation in periacetabular osteotomy.

Outcome	No predonation	Predonation effect	Relative effect (95% CI)	No. of studies (*k*)	Certainty (GRADE)	Comments
Autologous predonation versus no predonation						
Estimated blood loss	723 mL	No difference	MD: +170.8 (−157.0 to 498.5)	5	⊕◯◯◯ Very low	Very small sample
Intraoperative blood loss	771 mL	No difference	MD: +72.5 (−188.8 to 333.7)	8	⊕◯◯◯ Very low	High heterogeneity
Units transfused	1.1 units	No difference	MD ~ 0	4	⊕◯◯◯ Very low	Sparse data
Transfusion rate	7.0%	No difference	OR: 3.16 (0.64–15.53)	9	⊕◯◯◯ Very low	Very imprecise
Postoperative haemoglobin	102.5 g/L	↓ −5.5 g/L	MD: −5.5 (−9.0 to −1.9)	7	⊕⊕◯◯ Low	Only significant finding
Complications	2.7%	No difference	OR: 1.37 (0.15–12.29)	4	⊕◯◯◯ Very low	Very wide CI
THA conversion	—	Not estimable	—	—	⊕◯◯◯ Very low	Insufficient data

*Note*: Summary of effect estimates derived from arm‐based multilevel random‐effects meta‐analysis. Continuous outcomes are presented as mean differences (MD) and dichotomous outcomes as odds ratios (OR) with 95% confidence intervals (CI). Certainty of evidence was evaluated using the GRADE framework and was downgraded mainly due to risk of bias, small sample sizes, substantial heterogeneity and imprecision.

Abbreviations: GRADE, grading of recommendations assessment, development and evaluation; THA, total hip arthroplasty.

### TXA versus non‐TXA

#### Continuous outcomes

**Figure 2:**
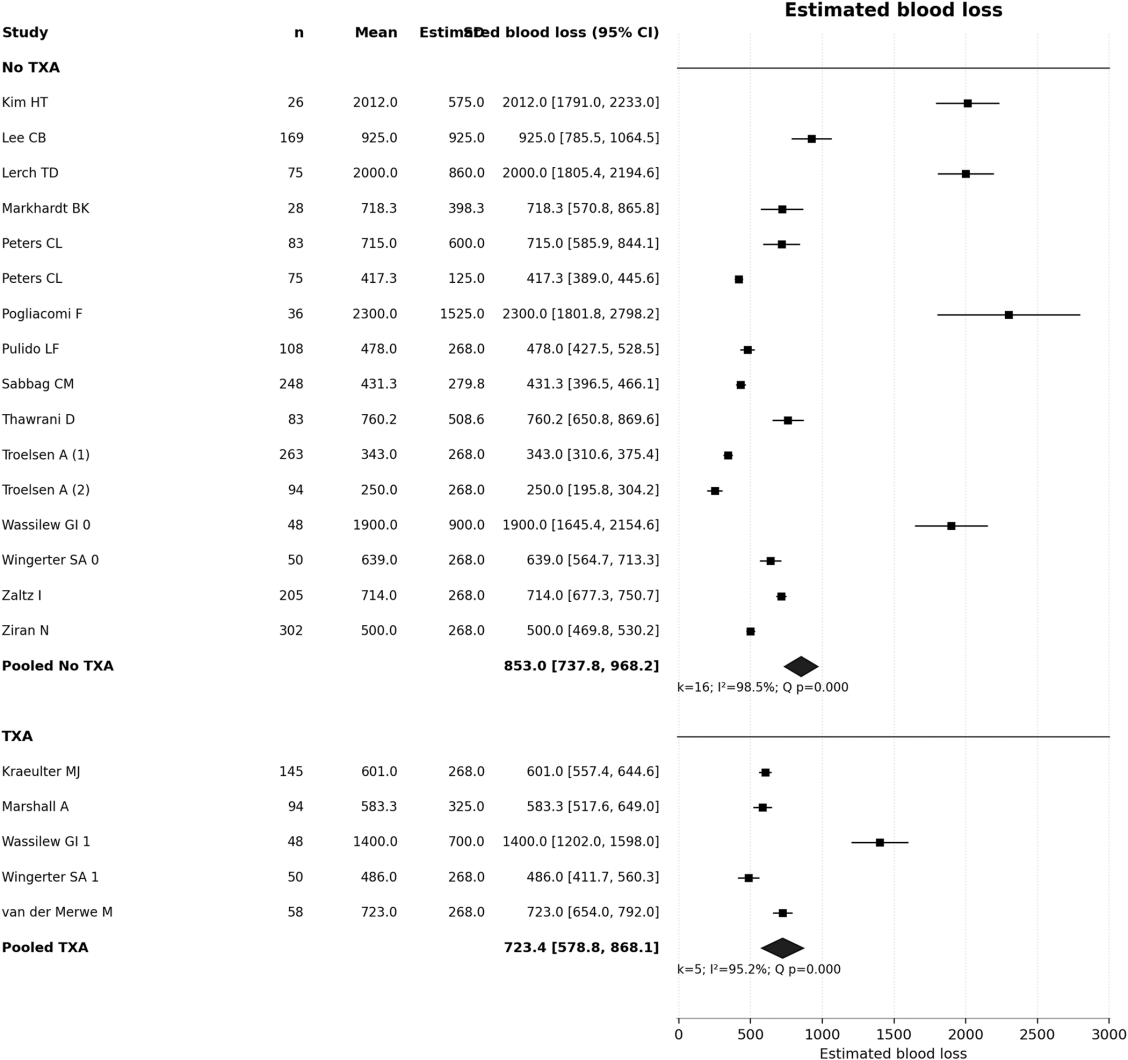
Forest plot: Estimated blood loss. Forest plot of the arm‐based multilevel random‐effects meta‐analysis comparing estimated blood loss between TXA and non‐TXA groups. TXA use was associated with significantly lower estimated blood loss. Effect sizes are presented as mean differences with 95% confidence intervals. Identical SD values across different studies reflect the predefined SD imputation procedure described in the Methods. SD, standard deviation; TXA, tranexamic acid.

**Figure 3:**
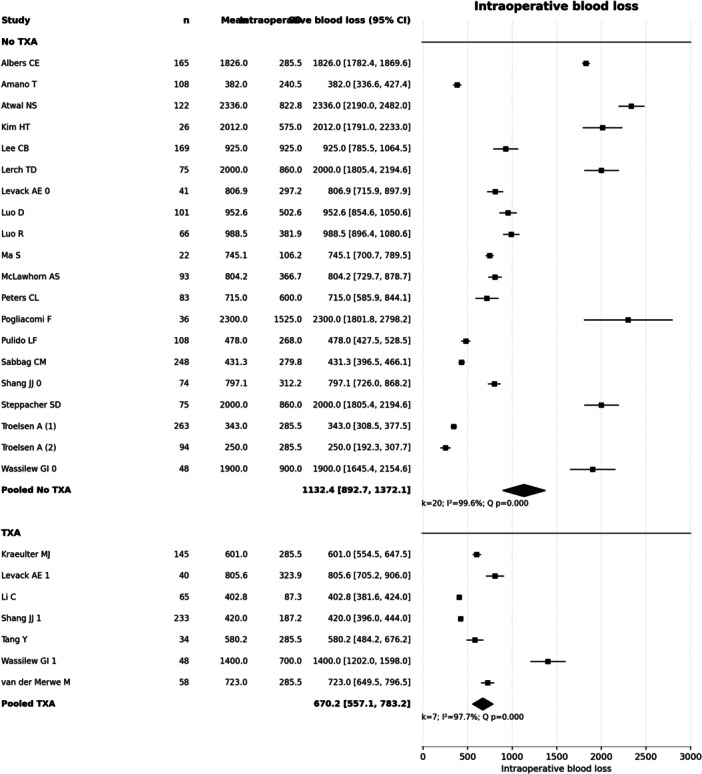
Forest plot: Intraoperative blood loss. Forest plot of the arm‐based multilevel random‐effects meta‐analysis comparing intraoperative blood loss between TXA and non‐TXA groups. TXA use was associated with significantly reduced intraoperative blood loss. Effect sizes are presented as mean differences with 95% confidence intervals. Identical SD values across different studies reflect the predefined SD imputation procedure described in the Methods. SD, standard deviation; TXA, tranexamic acid.

In the arm‐based multilevel meta‐analysis, TXA use was associated with significantly lower estimated blood loss (Figure 2) (mean difference −185.6 mL, 95% CI: −341.1 to −30.1; *p* = 0.022; *I* ^2^ = 98.2%) and intraoperative blood loss (Figure 3) (mean difference −299.4 mL, 95% CI: −392.2 to −206.6; *p* < 0.001; *I* ^2^ = 98.6%) compared with non‐TXA arms (Table 5).

**Table 5:** Arm‐based multilevel meta‐analysis of TXA and autologous predonation.

Outcome	TXA versus no‐TXA (effect)	*p*‐value	Predonation versus no (effect)	*p*‐value	Interpretation
Estimated blood loss	−185.6 mL (−341.1 to −30.1)	0.022	+170.8 mL (−157.0 to 498.5)	0.307	TXA associated with lower blood loss; no association observed for predonation
Intraoperative blood loss	−299.4 mL (−392.2 to −206.6)	<0.001	+72.5 mL (−188.8 to 333.7)	0.587	Consistent association in favour of TXA
Preop Hb	+1.5 g/L (−0.4 to 3.4)	0.105	−2.1 g/L (−7.5 to 3.2)	0.436	No significant association
Postop Hb	+2.5 g/L (−1.2 to 6.1)	0.167	−5.5 g/L (−9.0 to −1.9)	0.003	Predonation associated with lower postoperative haemoglobin
Hb drop	−0.1 g/L (−4.2–3.9)	0.945	+4.7 g/L (−1.7 to 11.0)	0.148	No consistent association
Units transfused	−0.67 units (−0.89 to −0.45)	<0.001	0.0 (−1.1 to 1.1)	0.965	TXA associated with lower transfusion rates
Transfusion rate	OR: 0.14 (0.056–0.338)	<0.001	OR: 3.16 (0.64–15.53)	0.155	Strong association in favour of TXA
Operation time	−5.2 min (−11.3 to 1.0)	0.096	+8.4 min (−36.0 to 52.7)	0.712	Not clinically relevant
Length of stay	−0.48 days (−1.07 to 0.11)	0.103	−1.7 days (−3.6 to 0.2)	0.075	Trend observed, but findings were inconsistent
Complications	OR: 0.86 (0.32–2.35)	0.767	OR: 1.37 (0.15–12.29)	0.776	No significant association
Reoperations	OR: 0.42 (0.04–4.39)	0.439	–	–	Insufficient data
THA conversion	OR: 0.16 (0.015–1.67)	0.118	–	–	Not statistically significant

*Note*: Results of the arm‐based multilevel random‐effects meta‐analysis comparing TXA versus non‐TXA and autologous predonation versus no predonation. Continuous outcomes are presented as mean differences with 95% confidence intervals (CIs), and dichotomous outcomes as odds ratios (ORs) with 95% CIs. Heterogeneity is reported using the *I* ^2^‐statistic. TXA was associated with reduced blood loss and transfusion outcomes, whereas no clinically relevant benefit was observed for autologous predonation.

Abbreviations: THA, total hip arthroplasty; TXA, tranexamic acid.

**Figure 4:**
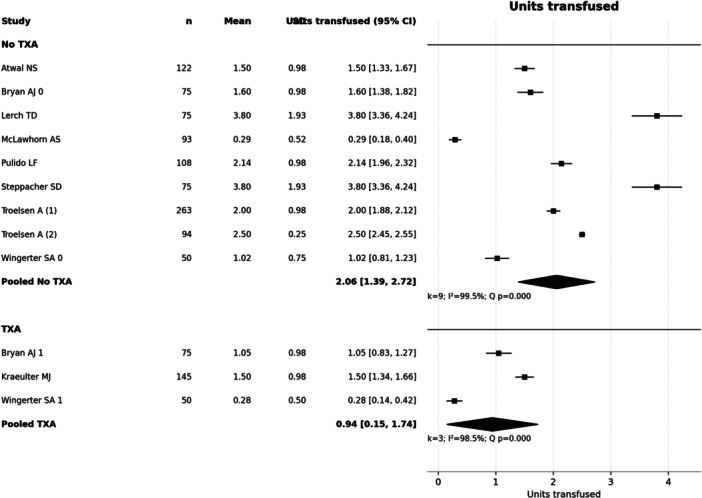
Forest plot: Units transfused. Forest plot of the arm‐based multilevel random‐effects meta‐analysis comparing the number of transfused units per patient between TXA and non‐TXA groups. TXA use was associated with significantly fewer transfused units. Effect sizes are presented as mean differences with 95% confidence intervals. Identical SD values across different studies reflect the predefined SD imputation procedure described in the Methods. SD, standard deviation; TXA, tranexamic acid.

No significant differences were observed for preoperative haemoglobin (+1.5 g/L, 95% CI: −0.4 to 3.4; *p* = 0.105; *I* ^2^ = 93.1%), postoperative haemoglobin (+2.5 g/L, 95% CI: −1.2 to 6.1; *p* = 0.167; *I* ^2^ = 81.6%) or haemoglobin drop (−0.1 g/L, 95% CI: −4.2 to 3.9; *p* = 0.945; *I* ^2^ = 96.9%) (Table 5). TXA use was associated with significantly fewer transfused units per patient (Figure 4) (mean difference −0.67 units, 95% CI: −0.89 to −0.45; *p* < 0.001; *I* ^2^ = 98.8%) (Table 5). No significant differences were found for operation time (−5.2 min, 95% CI: −11.3 to 1.0; *p* = 0.096; *I* ^2^ = 99.3%) or length of stay (−0.48 days, 95% CI: −1.07 to 0.11; *p* = 0.103; *I* ^2^ = 99.4%) (Table 5).

#### Binary outcomes

**Figure 5:**
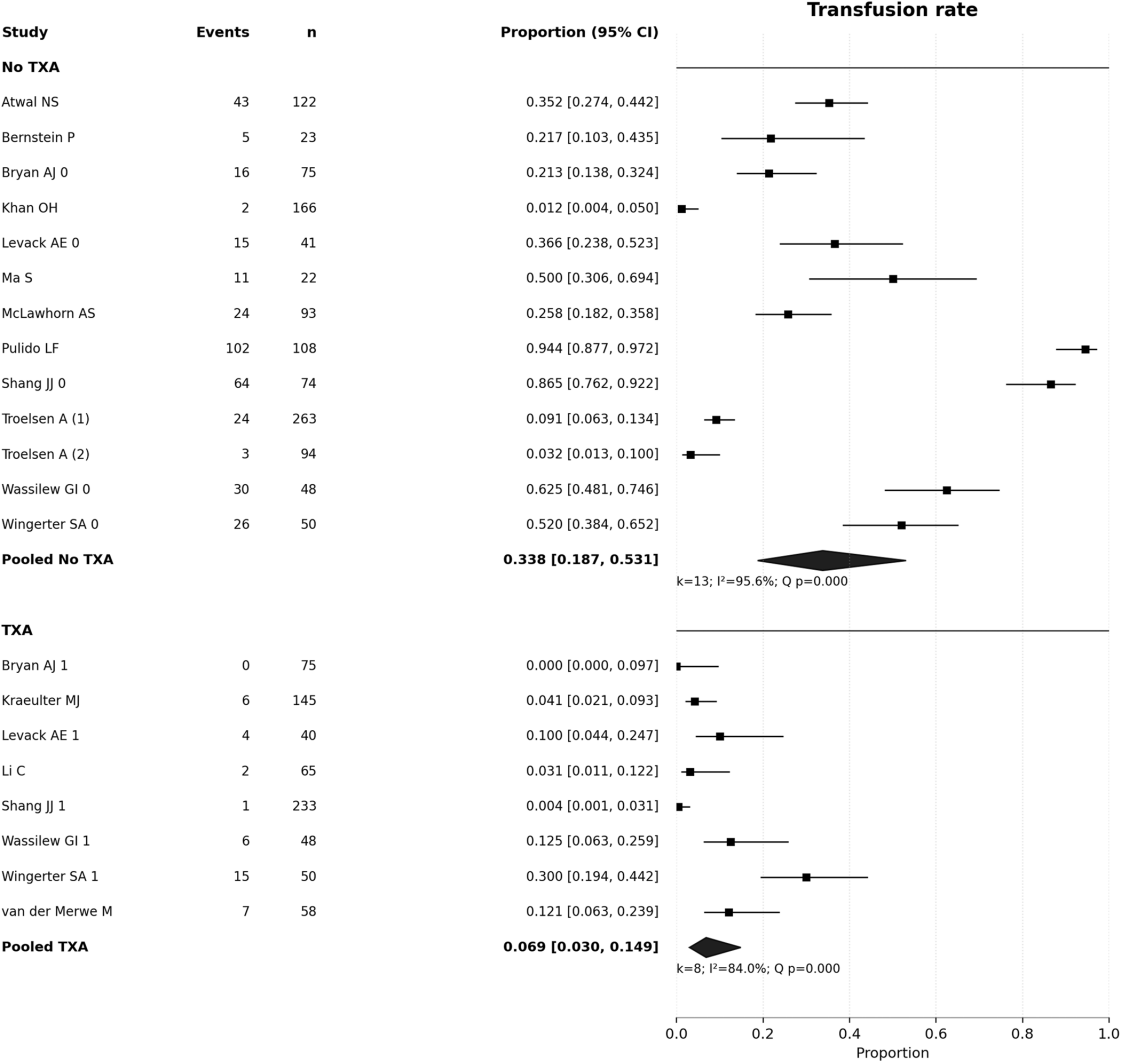
Forest plot: Transfusion rate. Forest plot of the arm‐based multilevel random‐effects meta‐analysis comparing transfusion rates between TXA and non‐TXA groups. TXA use was associated with a significantly lower transfusion rate. Effect sizes are presented as odds ratios with 95% confidence intervals. Note: Identical SD values across different studies reflect the predefined SD imputation procedure described in the Methods. SD, standard deviation; TXA, tranexamic acid.

TXA use was associated with a significantly lower transfusion rate (Figure 5) (OR: 0.14, 95% CI: 0.056–0.338; *p* < 0.001; *I* ^2^ = 89.6%) (Table 5). No significant differences were observed for complications (OR: 0.86, 95% CI: 0.32–2.35; *p* = 0.767; *I* ^2^ = 70.1%), reoperations (OR: 0.42, 95% CI: 0.041–4.39; *p* = 0.439; *I* ^2^ = 48.0%) or THA conversion (OR: 0.16, 95% CI: 0.015–1.67; *p* = 0.118; *I* ^2^ = 66.6%) (Table 5).

Forest plots for all additional TXA outcomes not shown in the main figures are available in the Supplementary Material (Figures S24–S31).

### Autologous predonation versus no predonation

#### Continuous outcomes

No significant differences were observed between predonation and no‐predonation arms for estimated blood loss (+170.8 mL, 95% CI: −157.0 to 498.5; *p* = 0.307; *I* ^2^ = 98.2%), intraoperative blood loss (+72.5 mL, 95% CI: −188.8 to 333.7; *p* = 0.587; *I* ^2^ = 98.7%), preoperative haemoglobin (−2.1 g/L, 95% CI: −7.5 to 3.2; *p* = 0.436; *I* ^2^ = 94.8%), haemoglobin drop (+4.7 g/L, 95% CI: −1.7 to 11.0; *p* = 0.148; *I* ^2^ = 91.7%), units transfused (*p* = 0.965; *I* ^2^ = 99.0%), operation time (*p* = 0.712; *I* ^2^ = 98.3%) or length of stay (*p* = 0.075; *I* ^2^ = 98.2%) (Table 5). Predonation was associated with significantly lower postoperative haemoglobin (mean difference −5.5 g/L, 95% CI: −9.0 to −1.9; *p* = 0.003; *I* ^2^ = 85.7%) (Table 5).

#### Binary outcomes

No significant differences were observed for transfusion rate (OR: 3.16, 95% CI: 0.64–15.53; *p* = 0.155; *I* ^2^ = 91.2%) or complications (OR: 1.37, 95% CI: 0.15–12.29; *p* = 0.776; *I* ^2^ = 65.0%) (Table 5). Data on THA conversion were insufficient for pooled analysis.

All forest plots for autologous predonation outcomes are presented in the Supplementary Material (Figures S32–S42). The sensitivity analysis restricted to studies with directly reported standard deviations yielded results consistent with the primary analyses, with no statistically significant differences observed.

## DISCUSSION

### Principal findings

The present arm‐based multilevel meta‐analysis demonstrates that TXA is associated with a clinically meaningful reduction in perioperative blood loss and transfusion requirements in PAO. In contrast, autologous predonation did not confer any measurable benefit and was even associated with lower postoperative haemoglobin levels.

The most robust findings were observed for intraoperative blood loss, estimated blood loss, transfusion rate and number of transfused units, all consistently favouring TXA. These effects were not only statistically significant but also clinically relevant, indicating that TXA may represent a useful component of perioperative blood management in PAO.

### Interpretation of TXA effects

The observed reduction in blood loss and transfusion requirements with TXA is consistent with its well‐established antifibrinolytic mechanism and aligns with findings from arthroplasty and trauma surgery. Given the extensive surgical exposure and cancellous bone bleeding associated with PAO, inhibition of fibrinolysis may be particularly relevant beneficial in this setting.

Importantly, the magnitude of effect observed in this analysis is substantial. The observed association with approximately 300 mL lower intraoperative blood loss and a lower transfusion rate may be clinically relevant. However, causal inference cannot be established because of the arm‐based design and the observational nature of most included studies.

Notably, TXA use did not significantly influence postoperative haemoglobin levels or haemoglobin drop. This apparent discrepancy may reflect the multifactorial nature of postoperative haemoglobin dynamics, including perioperative fluid shifts, dilutional effects and timing of measurement, rather than a lack of efficacy of TXA.

### Interpretation of predonation findings

In contrast to TXA, autologous predonation did not reduce blood loss or transfusion requirements. Across all relevant endpoints, no statistically significant benefit was observed.

Most notably, predonation was associated with significantly lower postoperative haemoglobin levels. This finding likely reflects preoperative depletion of red cell mass due to blood donation rather than a true perioperative disadvantage. Given the small number of available study arms and the very low certainty of evidence, this finding should be interpreted cautiously. In other words, predonation shifts anaemia from the postoperative to the preoperative phase without reducing overall blood loss or transfusion needs.

From a physiological perspective, this is plausible: predonation reduces baseline haemoglobin, thereby decreasing the patient's physiological reserve before surgery. In the absence of a compensatory reduction in blood loss or transfusion demand, this may result in lower postoperative haemoglobin values.

Despite the consistently favourable direction of effect for TXA, the certainty of evidence was limited by the predominance of nonrandomised studies and substantial heterogeneity. Therefore, the results should be interpreted as indicative rather than definitive. In contrast, the evidence regarding autologous predonation was uniformly of very low certainty, further limiting confidence in its clinical utility.

### Related literature

The present findings should be interpreted in the context of the existing systematic review literature on PAO. Previous meta‐analyses have consistently demonstrated that PAO is an effective joint‐preserving procedure with favourable survivorship and functional outcomes, albeit at the cost of considerable perioperative morbidity and complication rates [107]. In parallel, a growing body of systematic reviews has addressed specific aspects of PAO, including surgical technique, computer‐assisted modalities and patient selection, all of which underline the substantial heterogeneity in operative strategies and patient populations [77, 79]. This heterogeneity is likely to directly influence perioperative blood loss and transfusion requirements, yet these outcomes have not been comprehensively integrated into the broader PAO evidence base.

With regard to blood management, earlier meta‐analyses have suggested that antifibrinolytic agents are effective in reducing perioperative blood loss, haemoglobin decline and transfusion rates in PAO without increasing complication rates [113]. More recent analyses focusing specifically on TXA have confirmed a reduction in total blood loss and, in part, transfusion requirements [48, 122]. More recently, pooled benchmark estimates derived from the overall PAO literature demonstrated that PAO is associated with substantial perioperative blood loss and a clinically relevant transfusion burden, highlighting the need for effective perioperative blood management strategies. In that analysis, the pooled transfusion rate was 16.2%, with a mean intraoperative blood loss approaching 1 L and a mean estimated blood loss exceeding 800 mL. Furthermore, operative time was identified as a significant moderator of blood loss outcomes, whereas age and preoperative haemoglobin influenced transfusion‐related parameters [87]. However, these studies were limited by small sample sizes, inclusion of heterogeneous procedures such as high tibial osteotomy and a reliance on conventional pairwise comparisons, thereby restricting the ability to account for between‐study variability and to include single‐arm data.

Taken together, the current literature supports the potential role of TXA in PAO but remains methodologically limited and incomplete with respect to comprehensive blood management strategies. In particular, the role of alternative approaches such as autologous predonation has not been systematically evaluated, and comparative associations across heterogeneous study populations remains unclear.

### Comparison between strategies

The available evidence demonstrated a consistent association between TXA use and improved blood‐related outcomes, whereas no such association was observed for autologous predonation. These findings suggest that TXA may represent a useful component of perioperative blood management in PAO, although definitive recommendations are limited by the substantial heterogeneity and low certainty of evidence. Importantly, these findings reflect a broader paradigm shift in perioperative medicine, where predonation has largely been abandoned in favour of targeted pharmacological and intraoperative blood conservation strategies.

### Heterogeneity and robustness

A high degree of heterogeneity was observed across all outcomes (*I* ^2^ up to >98%). The very high heterogeneity observed across most analyses indicates that the pooled estimates should be interpreted as summary measures of highly diverse study populations and treatment protocols rather than precise effect estimates. This is expected in PAO research due to: (1) variability in surgical technique; (2) differences in perioperative protocols; (3) inconsistent use of adjunct blood management strategies; (4) heterogeneity in patient populations. Although the direction of effect was generally consistent across analyses, the substantial heterogeneity and predominance of nonrandomised studies reduce confidence in the precision of the pooled estimates. In contrast, the predonation analysis was limited by small sample sizes and sparse reporting, which further reduces confidence in its effectiveness.

### Clinical implications

These findings suggest that TXA may represent a useful component of perioperative blood management in PAO. However, given the substantial heterogeneity and low certainty of evidence, definitive recommendations cannot be made on the basis of the current literature. The present findings should also be interpreted in the context of recently established benchmark estimates for blood loss after PAO. Given the substantial transfusion burden reported across the overall PAO literature, the observed association between TXA use and lower blood loss and transfusion rates may be clinically relevant despite the limitations inherent to the available evidence [87].

### Limitations

Several limitations must be acknowledged: (1) The arm‐based design does not allow direct causal inference and is susceptible to confounding by indication; however, it enables inclusion of a broader evidence base and reflects real‐world heterogeneity; (2) Reporting of blood‐related outcomes was inconsistent across studies; (3) The predonation subgroup was small and underpowered; (4) High statistical heterogeneity limits the precision of the pooled estimates. Furthermore, the included studies spanned more than two decades, during which TXA administration protocols, perioperative management, surgical techniques and transfusion practices evolved substantially. As TXA‐containing study arms were predominantly derived from more recent studies, part of the observed treatment effect may reflect temporal changes in clinical practice rather than the isolated effect of TXA; (5) Multiple outcomes were evaluated without adjustment for multiplicity, which may increase the risk of type I error and should be considered when interpreting statistically significant findings; (6) Analyses were performed at the hip level because most primary studies reported outcomes per hip. Consequently, bilateral procedures could not be adjusted for separately, which may have introduced a degree of nonindependence into the pooled estimates.

## CONCLUSION

TXA use was associated with reduced blood loss and transfusion requirements after PAO, whereas autologous predonation was not associated with measurable clinical benefit. Given the substantial heterogeneity and low certainty of evidence, these findings should be interpreted cautiously.

## AUTHOR CONTRIBUTIONS

Nikolai Ramadanov and Jonathan Lettner performed the literature search, the data extraction and the risk of bias assessment. Nikolai Ramadanov conducted the statistical calculations. Nikolai Ramadanov created all figures and tables. Nikolai Ramadanov wrote the manuscript. Roland Becker, Robert Prill, Marko Ostojic and Sufian S. Ahmad supervised the work.

## CONFLICT OF INTEREST STATEMENT

The authors declare no conflicts of interest.

## ETHICS STATEMENT

The authors have nothing to report.

## Supporting information


**Supplementary Figure 1. Funnel plot: TXA ‐ Estimated blood loss.** Funnel plot of studies reporting estimated blood loss. Visual inspection suggests moderate asymmetry, with a relative absence of small studies reporting higher blood loss, indicating potential small‐study effects.

**Supplementary Figure 2. Funnel plot: TXA ‐ Intraoperative blood loss**. Funnel plot of studies reporting intraoperative blood loss. The distribution appears asymmetrical, with clustering of smaller studies toward lower blood loss values, suggesting potential small‐study effects or reporting bias.

**Supplementary Figure 3. Funnel plot: TXA ‐ Units transfused.** Funnel plot of studies reporting units transfused per patient. Mild asymmetry is observed, with some dispersion among smaller studies, indicating possible small‐study effects.

**Supplementary Figure 4. Funnel plot: TXA ‐ Transfusion rate.** Funnel plot of studies reporting transfusion rate. The distribution appears relatively symmetrical, with no clear evidence of substantial publication bias.

**Supplementary Figure 5. Funnel plot: TXA ‐ Preoperative hemoglobin.** Funnel plot of studies reporting preoperative hemoglobin levels. The plot shows asymmetry, with clustering toward higher hemoglobin values, suggesting potential selection or reporting bias.

**Supplementary Figure 6. Funnel plot: TXA ‐ Postoperative hemoglobin.** Funnel plot of studies reporting postoperative hemoglobin levels. Moderate asymmetry is present, indicating potential small‐study effects.

**Supplementary Figure 7. Funnel plot: TXA ‐ Hemoglobin drop.** Funnel plot of studies reporting hemoglobin drop. The distribution appears moderately symmetrical, with some dispersion among smaller studies; minor small‐study effects cannot be excluded.

**Supplementary Figure 8. Funnel plot: TXA ‐ Operation time.** Funnel plot of studies reporting operation time. The plot demonstrates asymmetry, with smaller studies tending toward shorter operative times, suggesting possible small‐study effects.

**Supplementary Figure 9. Funnel plot: TXA ‐ Length of stay.** Funnel plot of studies reporting length of stay. Mild asymmetry is observed, with no strong indication of substantial publication bias.

**Supplementary Figure 10. Funnel plot: TXA – Complications.** Funnel plot of studies reporting complication rates. The distribution is asymmetrical, with clustering of small studies at lower complication rates, suggesting potential reporting bias.

**Supplementary Figure 11. Funnel plot: TXA – Reoperations.** Funnel plot of studies reporting reoperation rates. The distribution appears relatively symmetrical, with no clear evidence of major publication bias.

**Supplementary Figure 12. Funnel plot: TXA ‐ THA conversion.** Funnel plot of studies reporting conversion to total hip arthroplasty. Moderate asymmetry is present, indicating possible small‐study effects.

**Supplementary Figure 13. Funnel plot: Autologous predonation ‐ Estimated blood loss.** Funnel plot of studies reporting estimated blood loss. The very small number of studies and visible asymmetry preclude reliable assessment of publication bias.

**Supplementary Figure 14. Funnel plot: Autologous predonation ‐ Intraoperative blood loss.** Funnel plot of studies reporting intraoperative blood loss. Due to the limited number of studies, no meaningful conclusions regarding symmetry or publication bias can be drawn.

**Supplementary Figure 15. Funnel plot: Autologous predonation ‐ Units transfused.** Funnel plot of studies reporting units transfused per patient. The sparse distribution and small sample size limit interpretability; publication bias cannot be assessed.

**Supplementary Figure 16. Funnel plot: Autologous predonation ‐ Transfusion rate.** Funnel plot of studies reporting transfusion rate. The low number of included studies and uneven distribution prevent reliable interpretation of small‐study effects.

**Supplementary Figure 17. Funnel plot: Autologous predonation ‐ Preoperative hemoglobin.** Funnel plot of studies reporting preoperative hemoglobin levels. Interpretation is limited by the small number of studies and apparent asymmetry.

**Supplementary Figure 18. Funnel plot: Autologous predonation ‐ Postoperative hemoglobin.** Funnel plot of studies reporting postoperative hemoglobin levels. The limited dataset precludes meaningful assessment of publication bias.

**Supplementary Figure 19. Funnel plot: Autologous predonation ‐ Hemoglobin drop.** Funnel plot of studies reporting hemoglobin drop. Due to the small number of studies, conclusions regarding symmetry or small‐study effects are not reliable.

**Supplementary Figure 20. Funnel plot: Autologous predonation ‐ Operation time.** Funnel plot of studies reporting operation time. The small number of data points limits interpretability and prevents meaningful conclusions regarding publication bias.

**Supplementary Figure 21. Funnel plot: Autologous predonation ‐ Length of stay.** Funnel plot of studies reporting length of stay. Interpretation is limited due to the very small number of included studies.

**Supplementary Figure 22. Funnel plot: Autologous predonation – Complications.** Funnel plot of studies reporting complications. The sparse data do not allow reliable assessment of funnel plot symmetry.

**Supplementary Figure 23. Funnel plot: Autologous predonation ‐ THA conversion.** Funnel plot of studies reporting conversion to total hip arthroplasty. With only minimal data available, no conclusions regarding publication bias can be drawn.

**Supplementary Figure 24. Forest plot: TXA Preoperative hemoglobin.** Forest plot of the arm‐based multilevel random‐effects meta‐analysis comparing preoperative hemoglobin levels between TXA and non‐TXA groups. No statistically significant difference was observed. Effect sizes are presented as mean differences with 95% confidence intervals.

**Supplementary Figure 25. Forest plot: TXA ‐ Postoperative hemoglobin.** Forest plot of the arm‐based multilevel random‐effects meta‐analysis comparing postoperative hemoglobin levels between TXA and non‐TXA groups. No statistically significant difference was observed. Effect sizes are presented as mean differences with 95% confidence intervals.

**Supplementary Figure 26. Forest plot: TXA ‐ Hemoglobin drop.** Forest plot of the arm‐based multilevel random‐effects meta‐analysis comparing hemoglobin decrease between TXA and non‐TXA groups. No statistically significant difference was observed. Effect sizes are presented as mean differences with 95% confidence intervals.

**Supplementary Figure 27. Forest plot: TXA ‐ Operation time.** Forest plot of the arm‐based multilevel random‐effects meta‐analysis comparing operation time between TXA and non‐TXA groups. No statistically significant difference was observed. Effect sizes are presented as mean differences with 95% confidence intervals.

**Supplementary Figure 28. Forest plot: TXA ‐ Length of stay.** Forest plot of the arm‐based multilevel random‐effects meta‐analysis comparing length of hospital stay between TXA and non‐TXA groups. No statistically significant difference was observed. Effect sizes are presented as mean differences with 95% confidence intervals.

**Supplementary Figure 29. Forest plot: TXA ‐ Complications.** Forest plot of the arm‐based multilevel random‐effects meta‐analysis comparing complication rates between TXA and non‐TXA groups. No statistically significant difference was observed. Effect sizes are presented as odds ratios with 95% confidence intervals.

**Supplementary Figure 30. Forest plot: TXA ‐ Reoperations.** Forest plot of the arm‐based multilevel random‐effects meta‐analysis comparing reoperation rates between TXA and non‐TXA groups. No statistically significant difference was observed. Effect sizes are presented as odds ratios with 95% confidence intervals.

**Supplementary Figure 31. Forest plot: TXA ‐ THA conversion.** Forest plot of the arm‐based multilevel random‐effects meta‐analysis comparing conversion to total hip arthroplasty (THA) between TXA and non‐TXA groups. No statistically significant difference was observed. Effect sizes are presented as odds ratios with 95% confidence intervals.

**Supplementary Figure 32. Forest plot: Autologous predonation – estimated blood loss.** Forest plot of the arm‐based multilevel random‐effects meta‐analysis comparing estimated blood loss between predonation and no‐predonation groups. No statistically significant difference was observed. Effect sizes are presented as mean differences with 95% confidence intervals.

**Supplementary Figure 33. Forest plot: Autologous predonation – intraoperative blood loss.** Forest plot of the arm‐based multilevel random‐effects meta‐analysis comparing intraoperative blood loss between predonation and no‐predonation groups. No statistically significant difference was observed. Effect sizes are presented as mean differences with 95% confidence intervals.

**Supplementary Figure 34. Forest plot: Autologous predonation – units transfused.** Forest plot of the arm‐based multilevel random‐effects meta‐analysis comparing the number of transfused units per patient between predonation and no‐predonation groups. No statistically significant difference was observed. Effect sizes are presented as mean differences with 95% confidence intervals.

**Supplementary Figure 35. Forest plot: Autologous predonation – transfusion rate.** Forest plot of the arm‐based multilevel random‐effects meta‐analysis comparing transfusion rates between predonation and no‐predonation groups. No statistically significant difference was observed. Effect sizes are presented as odds ratios with 95% confidence intervals.

**Supplementary Figure 36. Forest plot: Autologous predonation – preoperative hemoglobin.** Forest plot of the arm‐based multilevel random‐effects meta‐analysis comparing preoperative hemoglobin levels between predonation and no‐predonation groups. No statistically significant difference was observed. Effect sizes are presented as mean differences with 95% confidence intervals.

**Supplementary Figure 37. Forest plot: Autologous predonation – postoperative hemoglobin.** Forest plot of the arm‐based multilevel random‐effects meta‐analysis comparing postoperative hemoglobin levels between predonation and no‐predonation groups. Predonation was associated with significantly lower postoperative hemoglobin. Effect sizes are presented as mean differences with 95% confidence intervals.

**Supplementary Figure 38. Forest plot: Autologous predonation – hemoglobin drop.** Forest plot of the arm‐based multilevel random‐effects meta‐analysis comparing hemoglobin decrease between predonation and no‐predonation groups. No statistically significant difference was observed. Effect sizes are presented as mean differences with 95% confidence intervals.

**Supplementary Figure 39. Forest plot: Autologous predonation – operation time.** Forest plot of the arm‐based multilevel random‐effects meta‐analysis comparing operation time between predonation and no‐predonation groups. No statistically significant difference was observed. Effect sizes are presented as mean differences with 95% confidence intervals.

**Supplementary Figure 40. Forest plot: Autologous predonation – length of stay.** Forest plot of the arm‐based multilevel random‐effects meta‐analysis comparing length of hospital stay between predonation and no‐predonation groups. No statistically significant difference was observed. Effect sizes are presented as mean differences with 95% confidence intervals.

**Supplementary Figure 41. Forest plot: Autologous predonation – complications.** Forest plot of the arm‐based multilevel random‐effects meta‐analysis comparing complication rates between predonation and no‐predonation groups. No statistically significant difference was observed. Effect sizes are presented as odds ratios with 95% confidence intervals.

**Supplementary Figure 42. Forest plot: Autologous predonation – THA conversion.** Forest plot of the arm‐based multilevel random‐effects meta‐analysis comparing conversion to total hip arthroplasty (THA) between predonation and no‐predonation groups. Data were insufficient for robust pooled analysis.

Supplementary Table 1: PRISMA checklist.

**Supplementary Table 2: Availability of standard deviation (SD) data for each included study.** The table indicates whether SDs were directly reported or imputed according to the predefined imputation strategy described in the Methods.

## Data Availability

Available from the corresponding author upon reasonable request.
