# Supplementary figures and images for: Association of tranexamic acid use and autologous predonation with blood loss and transfusion outcomes after periacetabular osteotomy: An arm‐based multilevel meta analysis
Source: J Exp Orthop. 2026 Jul 28;13(3):e70867. doi: 10.1002/jeo2.70867 (PMC13410948; doi:10.1002/jeo2.70867)

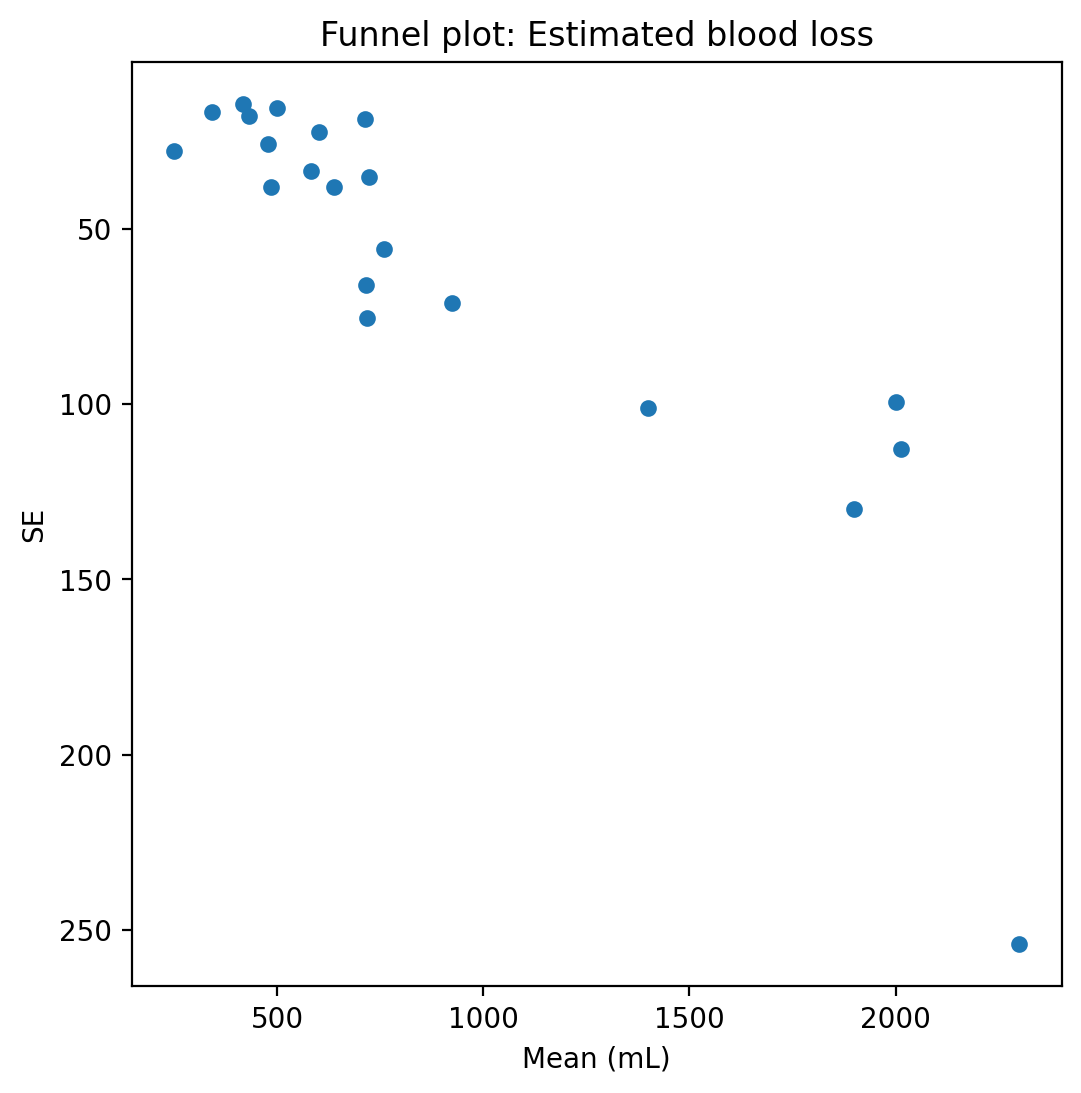

Supplement: Supplementary file 1 — Supplementary Figure 1. Funnel plot: TXA ‐ Estimated blood loss. Funnel plot of studies reporting estimated blood loss. Visual inspection suggests moderate asymmetry, with a relative absence of small studies reporting higher blood loss, indicating potential small‐study effects. [file JEO2-13-e70867-s001.png]

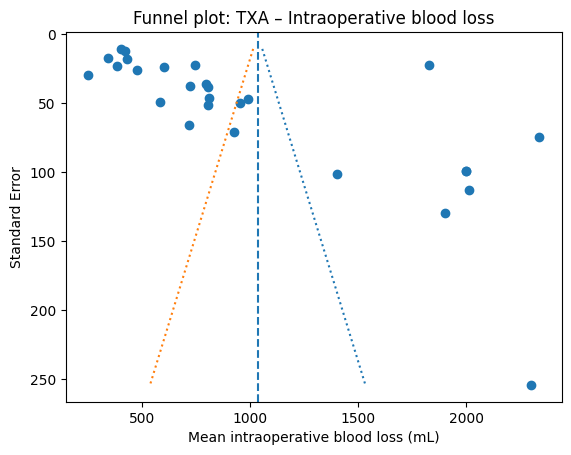

Supplement: Supplementary file 2 — Supplementary Figure 2. Funnel plot: TXA ‐ Intraoperative blood loss. Funnel plot of studies reporting intraoperative blood loss. The distribution appears asymmetrical, with clustering of smaller studies toward lower blood loss values, suggesting potential small‐study effects or reporting bias. [file JEO2-13-e70867-s004.png]

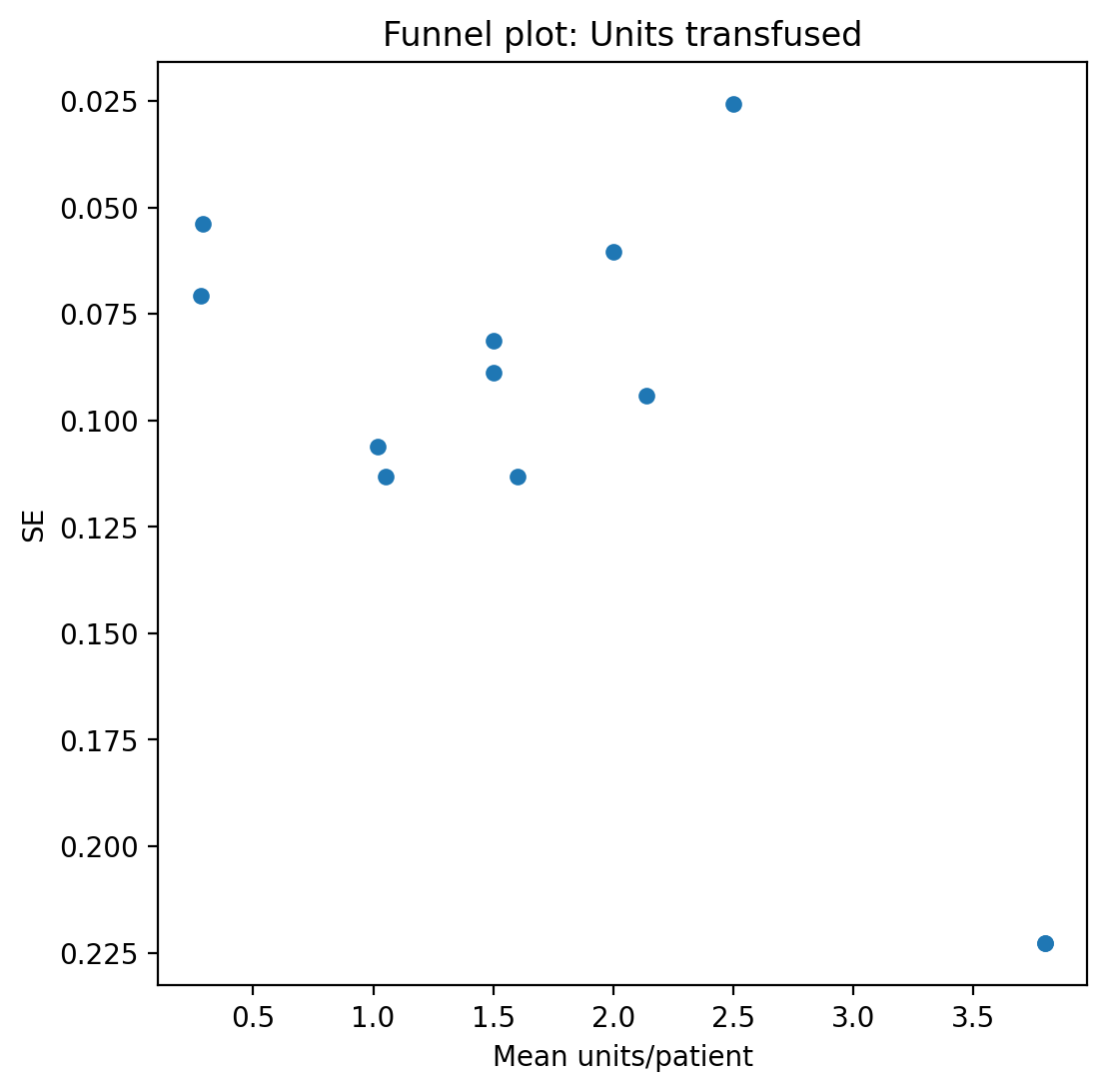

Supplement: Supplementary file 3 — Supplementary Figure 3. Funnel plot: TXA ‐ Units transfused. Funnel plot of studies reporting units transfused per patient. Mild asymmetry is observed, with some dispersion among smaller studies, indicating possible small‐study effects. [file JEO2-13-e70867-s027.png]

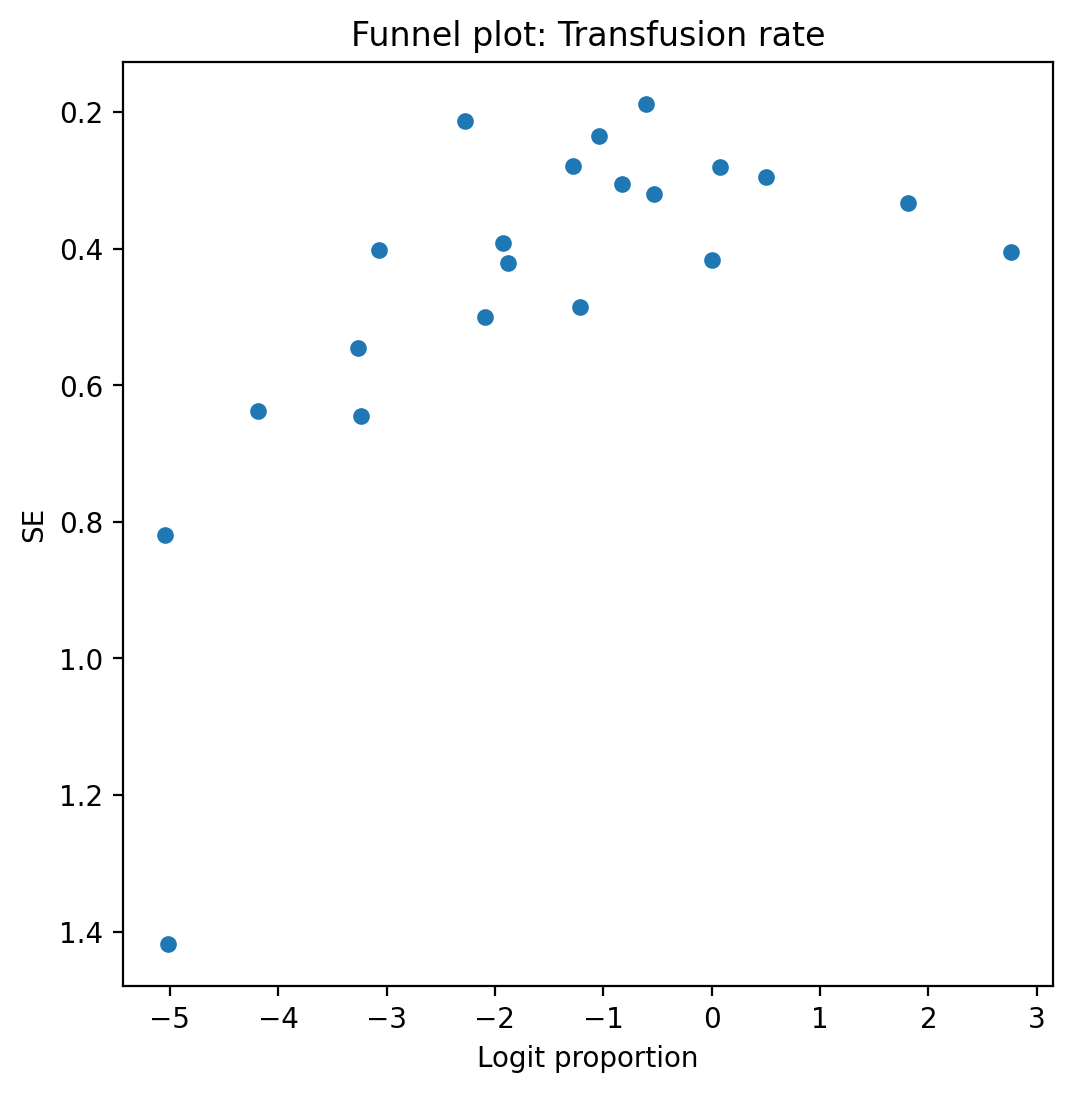

Supplement: Supplementary file 4 — Supplementary Figure 4. Funnel plot: TXA ‐ Transfusion rate. Funnel plot of studies reporting transfusion rate. The distribution appears relatively symmetrical, with no clear evidence of substantial publication bias. [file JEO2-13-e70867-s010.png]

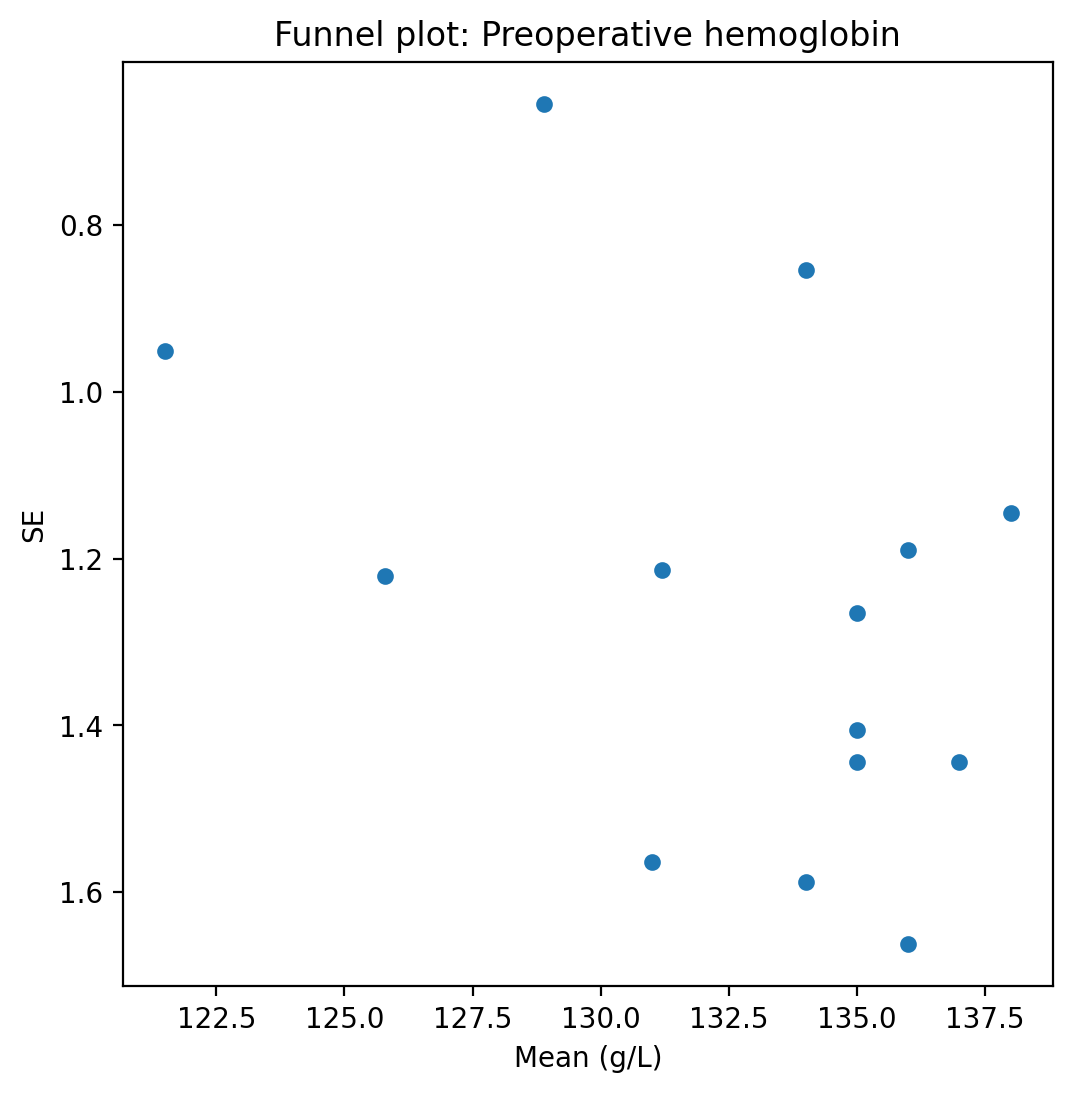

Supplement: Supplementary file 5 — Supplementary Figure 5. Funnel plot: TXA ‐ Preoperative hemoglobin. Funnel plot of studies reporting preoperative hemoglobin levels. The plot shows asymmetry, with clustering toward higher hemoglobin values, suggesting potential selection or reporting bias. [file JEO2-13-e70867-s022.png]

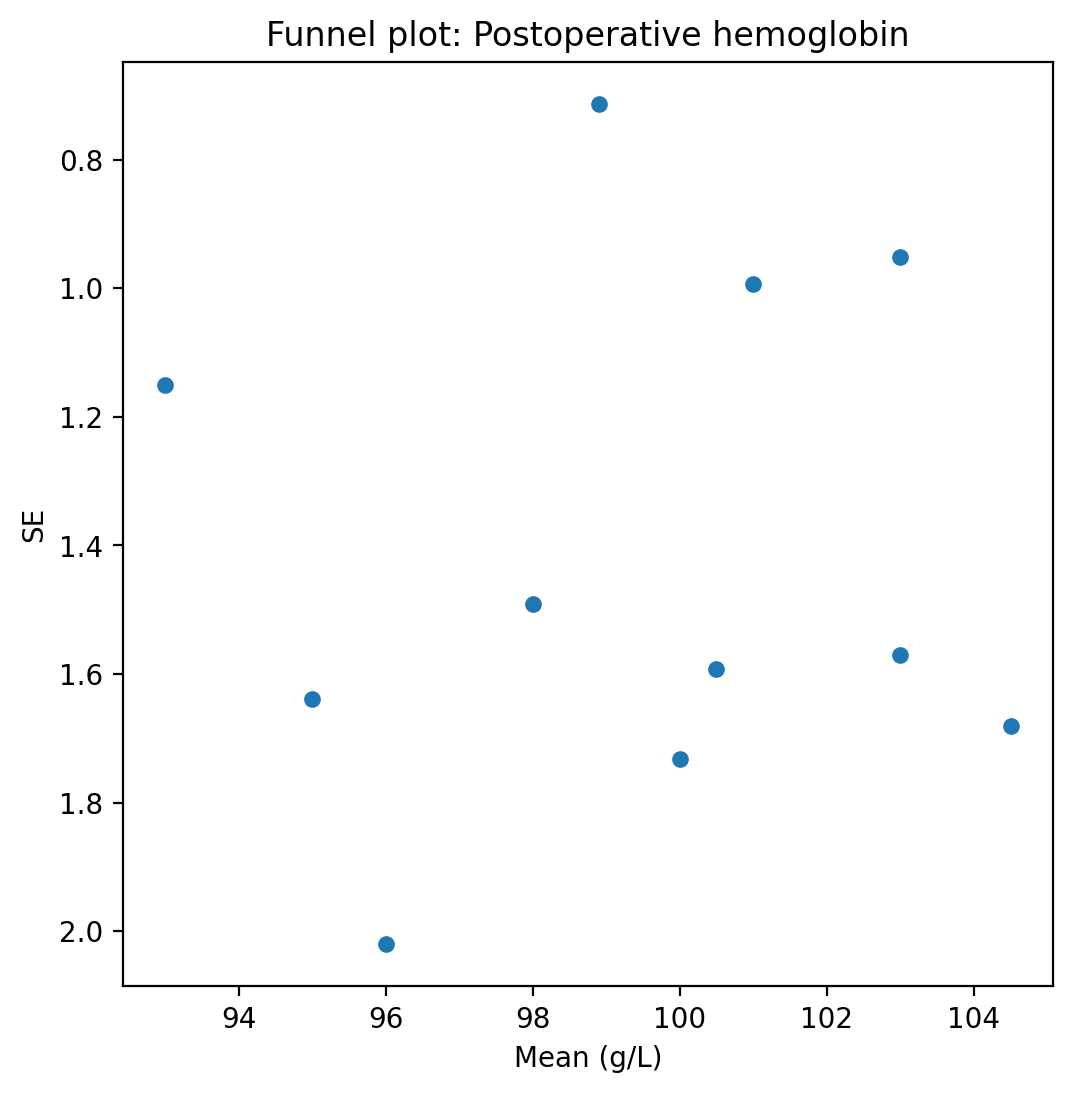

Supplement: Supplementary file 6 — Supplementary Figure 6. Funnel plot: TXA ‐ Postoperative hemoglobin. Funnel plot of studies reporting postoperative hemoglobin levels. Moderate asymmetry is present, indicating potential small‐study effects. [file JEO2-13-e70867-s025.png]

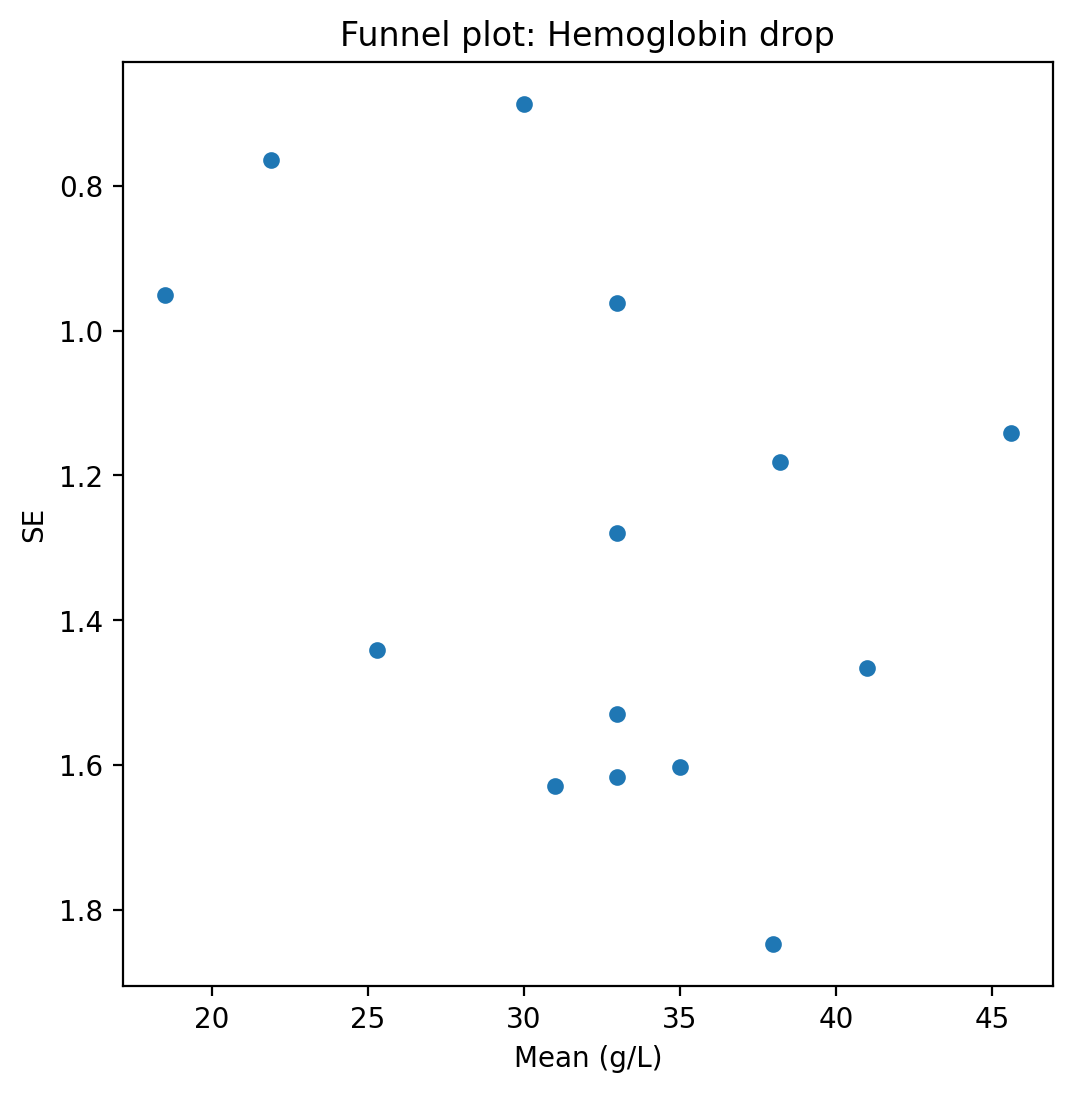

Supplement: Supplementary file 7 — Supplementary Figure 7. Funnel plot: TXA ‐ Hemoglobin drop. Funnel plot of studies reporting hemoglobin drop. The distribution appears moderately symmetrical, with some dispersion among smaller studies; minor small‐study effects cannot be excluded. [file JEO2-13-e70867-s007.png]

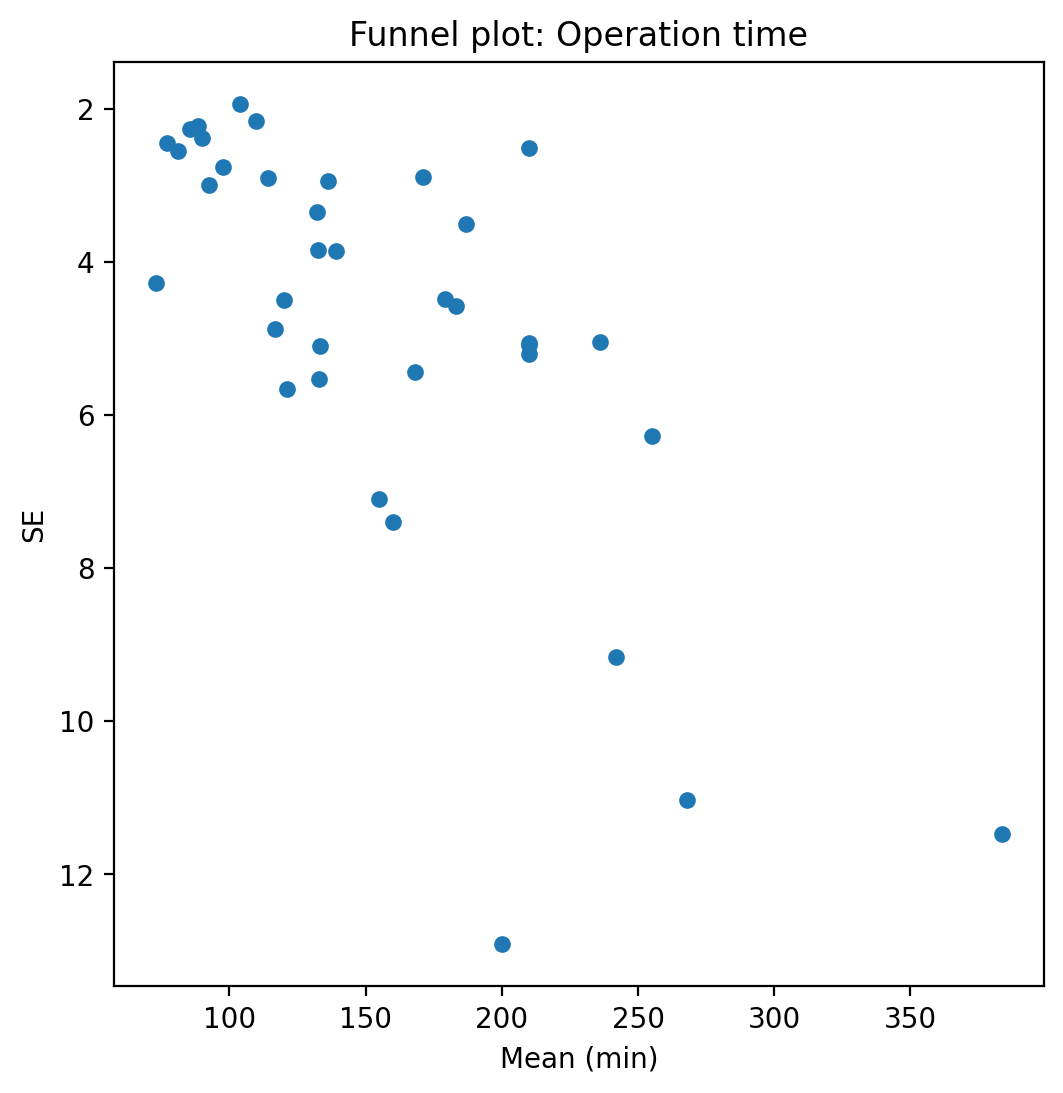

Supplement: Supplementary file 8 — Supplementary Figure 8. Funnel plot: TXA ‐ Operation time. Funnel plot of studies reporting operation time. The plot demonstrates asymmetry, with smaller studies tending toward shorter operative times, suggesting possible small‐study effects. [file JEO2-13-e70867-s016.png]

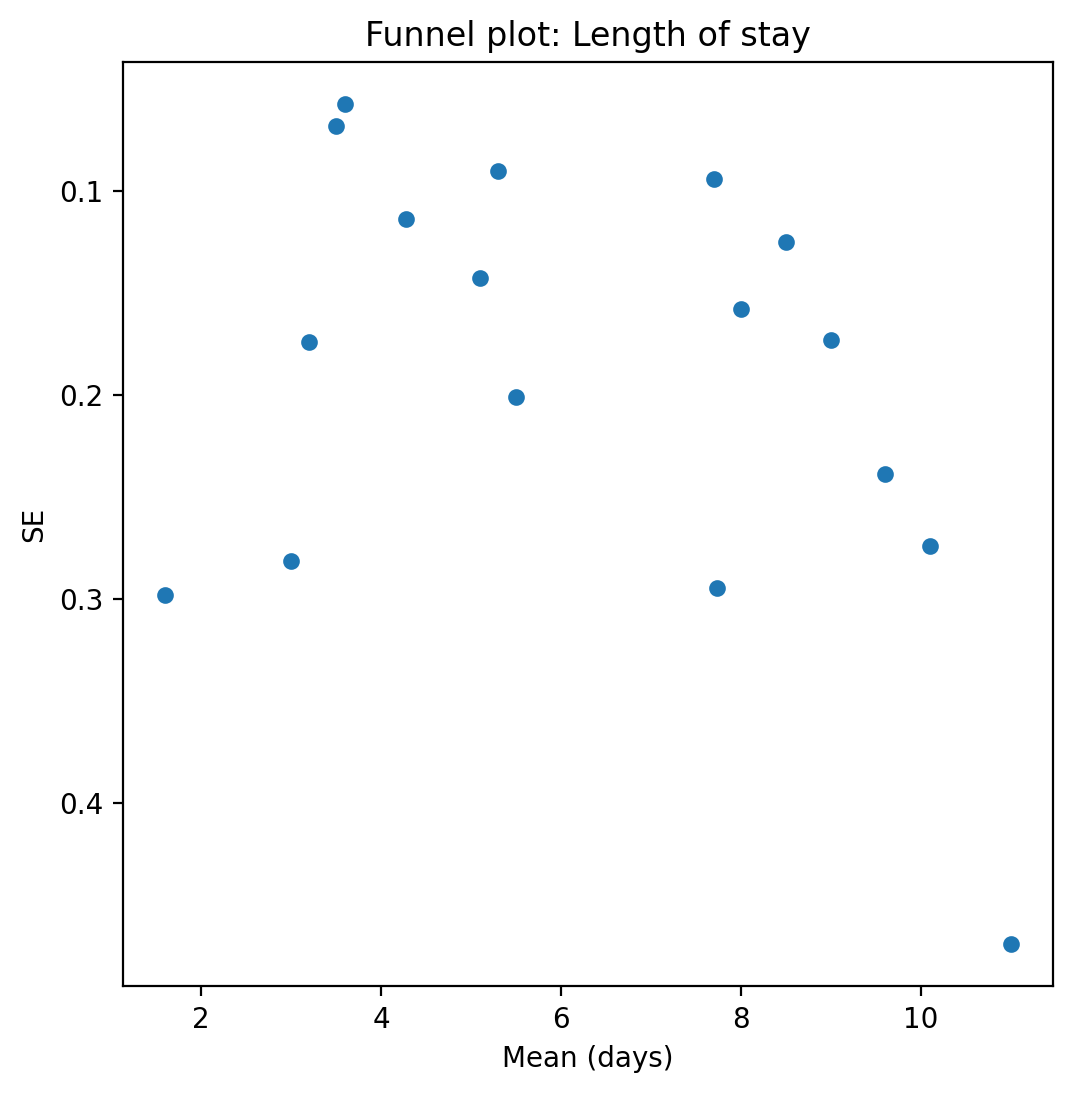

Supplement: Supplementary file 9 — Supplementary Figure 9. Funnel plot: TXA ‐ Length of stay. Funnel plot of studies reporting length of stay. Mild asymmetry is observed, with no strong indication of substantial publication bias. [file JEO2-13-e70867-s036.png]

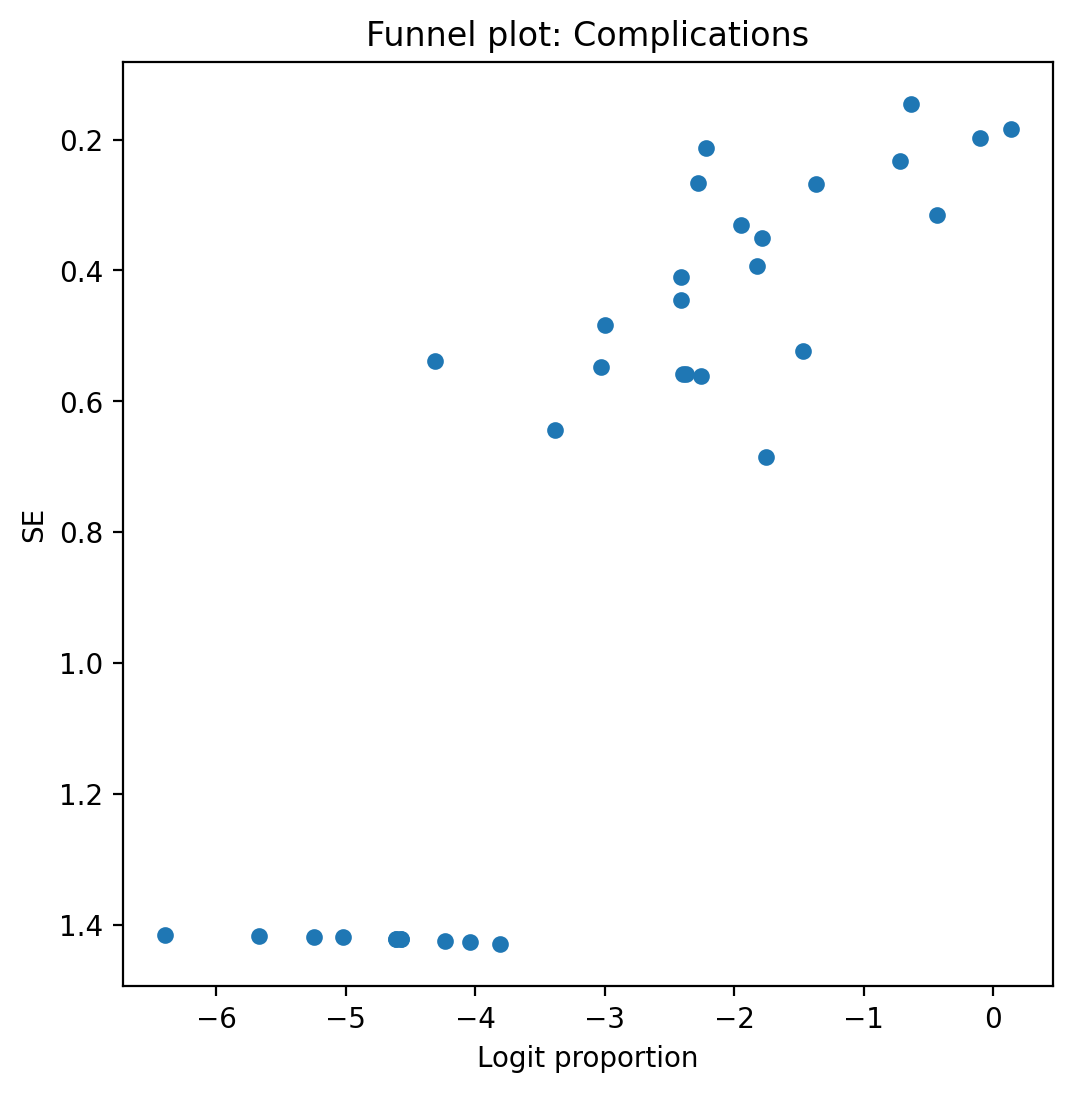

Supplement: Supplementary file 10 — Supplementary Figure 10. Funnel plot: TXA – Complications. Funnel plot of studies reporting complication rates. The distribution is asymmetrical, with clustering of small studies at lower complication rates, suggesting potential reporting bias. [file JEO2-13-e70867-s038.png]

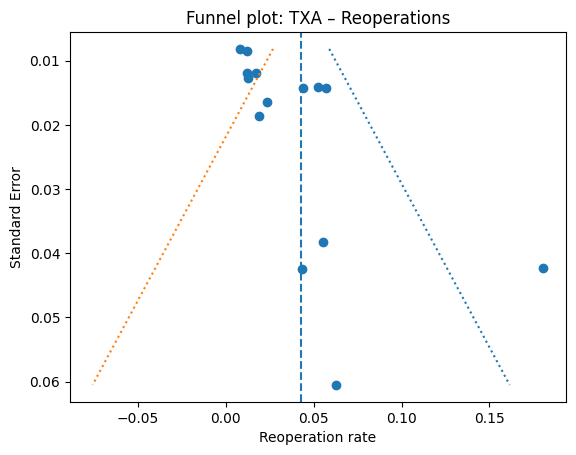

Supplement: Supplementary file 11 — Supplementary Figure 11. Funnel plot: TXA – Reoperations. Funnel plot of studies reporting reoperation rates. The distribution appears relatively symmetrical, with no clear evidence of major publication bias. [file JEO2-13-e70867-s012.png]

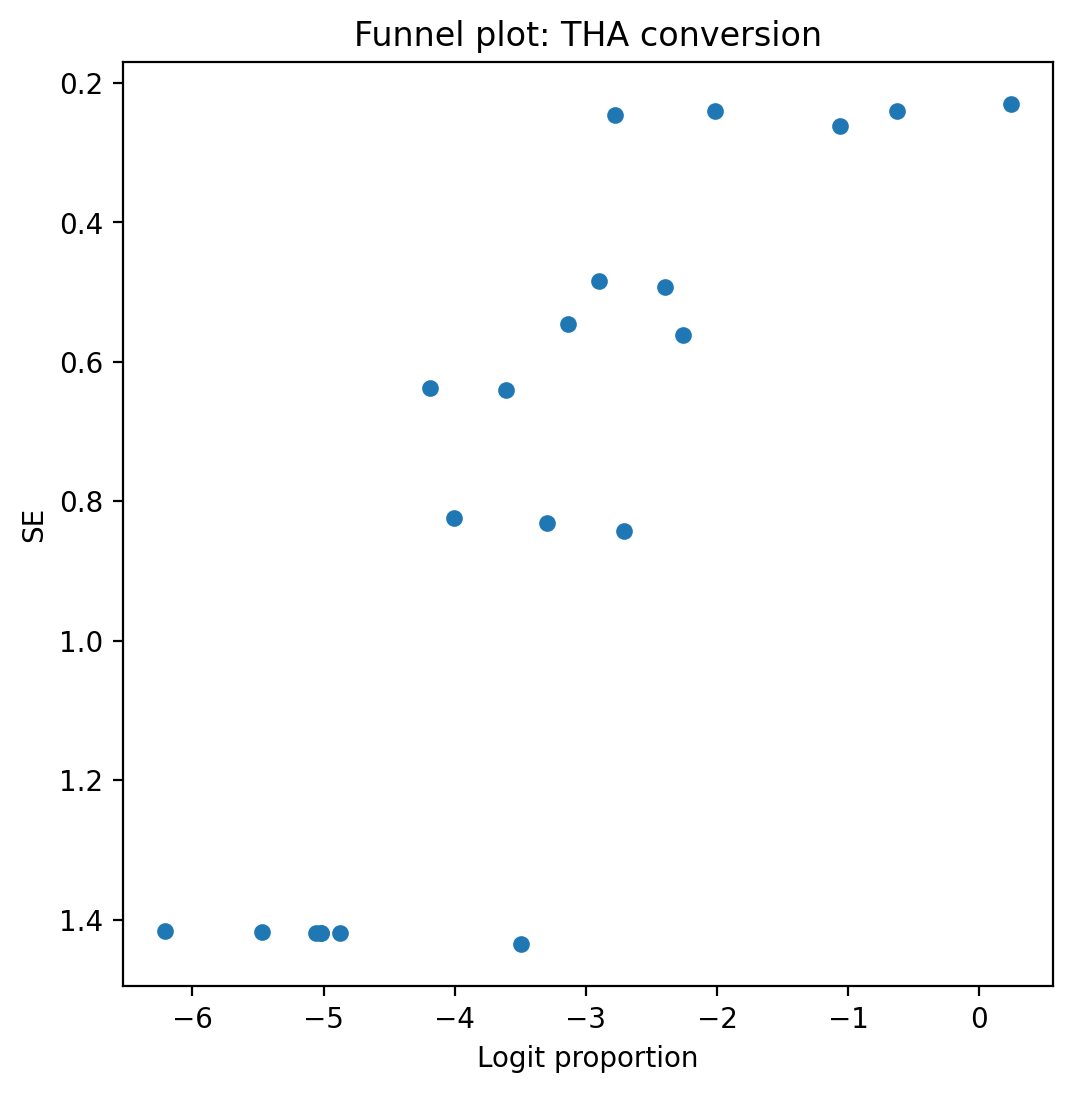

Supplement: Supplementary file 12 — Supplementary Figure 12. Funnel plot: TXA ‐ THA conversion. Funnel plot of studies reporting conversion to total hip arthroplasty. Moderate asymmetry is present, indicating possible small‐study effects. [file JEO2-13-e70867-s026.png]

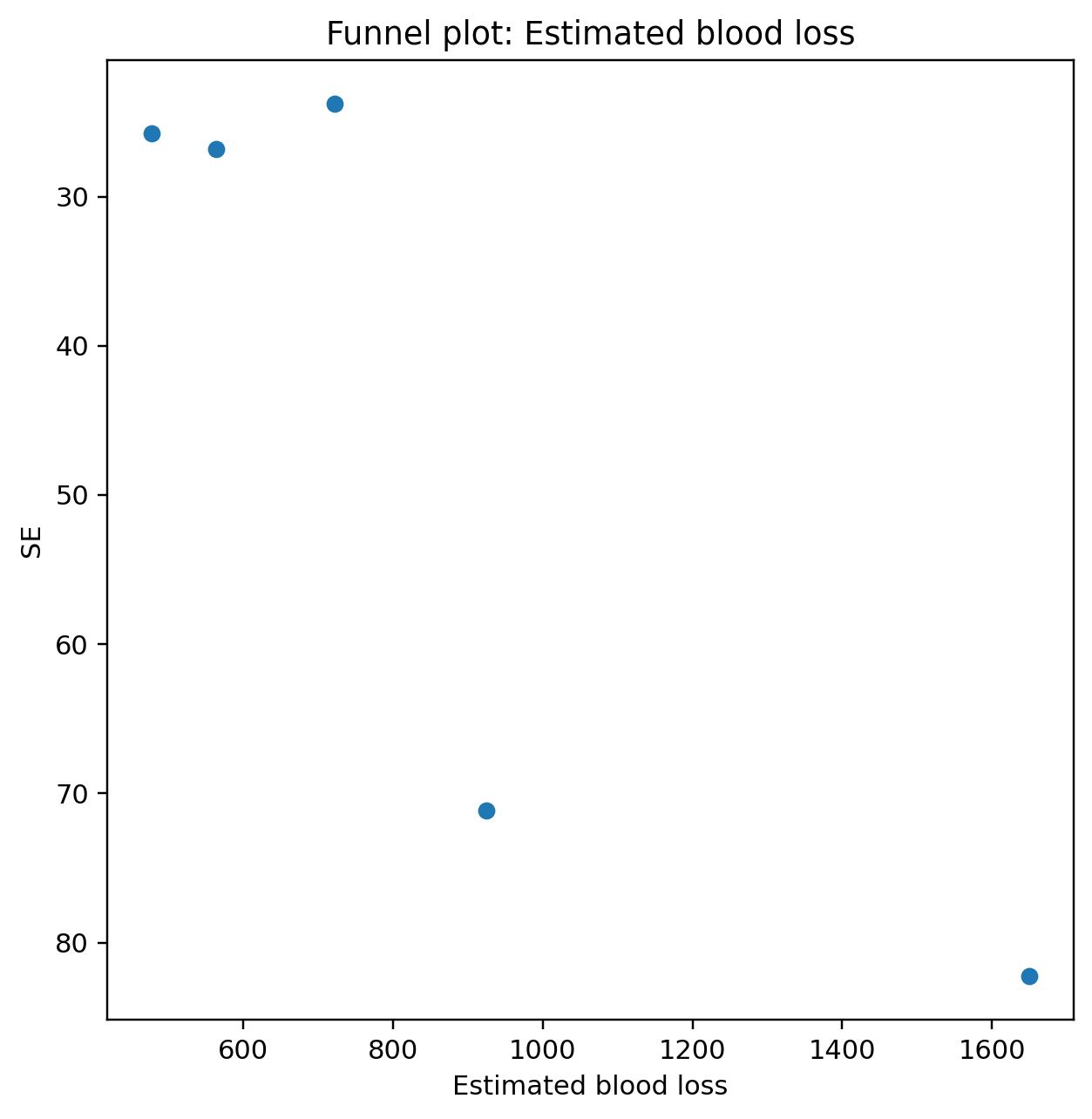

Supplement: Supplementary file 13 — Supplementary Figure 13. Funnel plot: Autologous predonation ‐ Estimated blood loss. Funnel plot of studies reporting estimated blood loss. The very small number of studies and visible asymmetry preclude reliable assessment of publication bias. [file JEO2-13-e70867-s019.png]

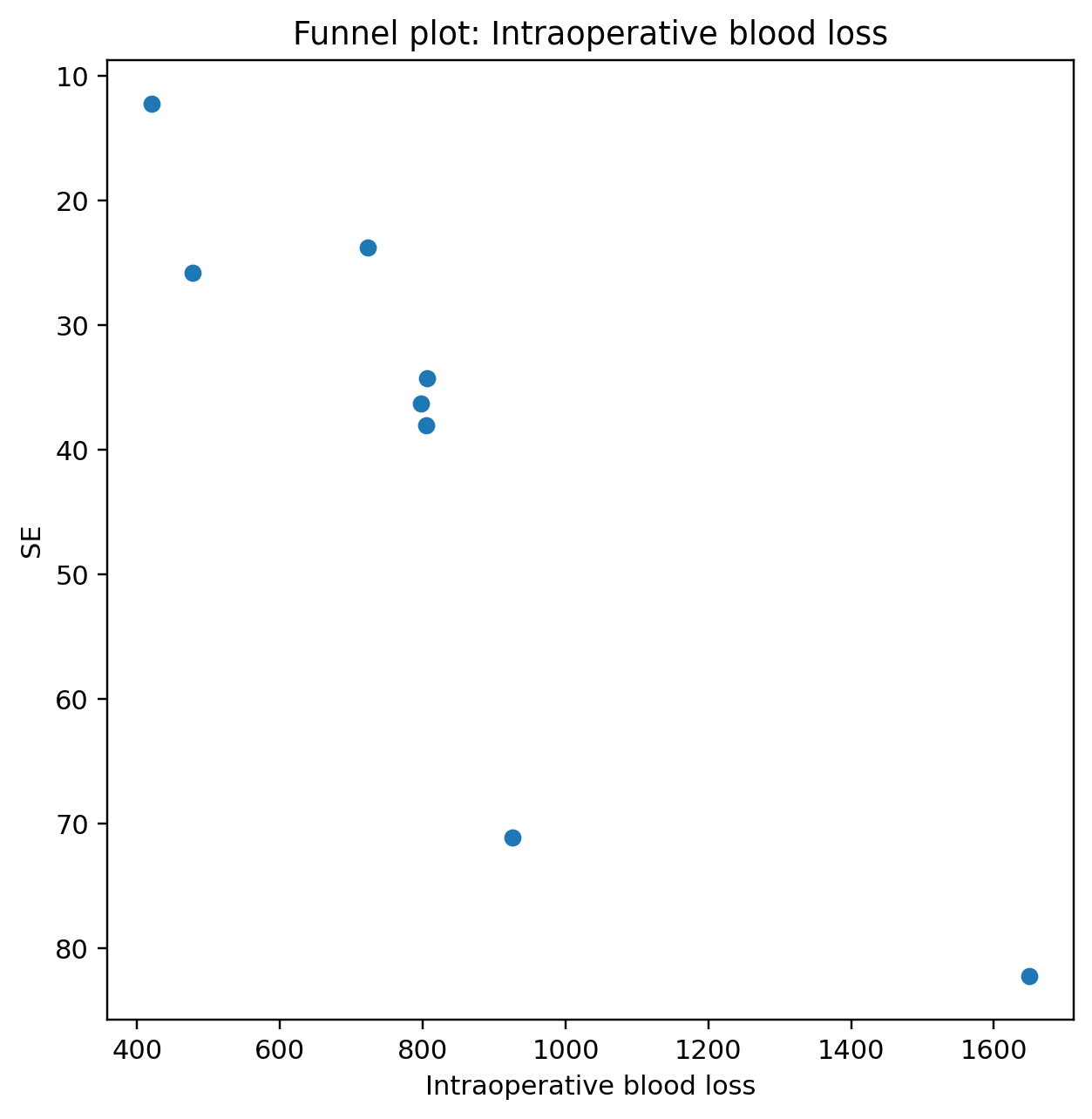

Supplement: Supplementary file 14 — Supplementary Figure 14. Funnel plot: Autologous predonation ‐ Intraoperative blood loss. Funnel plot of studies reporting intraoperative blood loss. Due to the limited number of studies, no meaningful conclusions regarding symmetry or publication bias can be drawn. [file JEO2-13-e70867-s006.png]

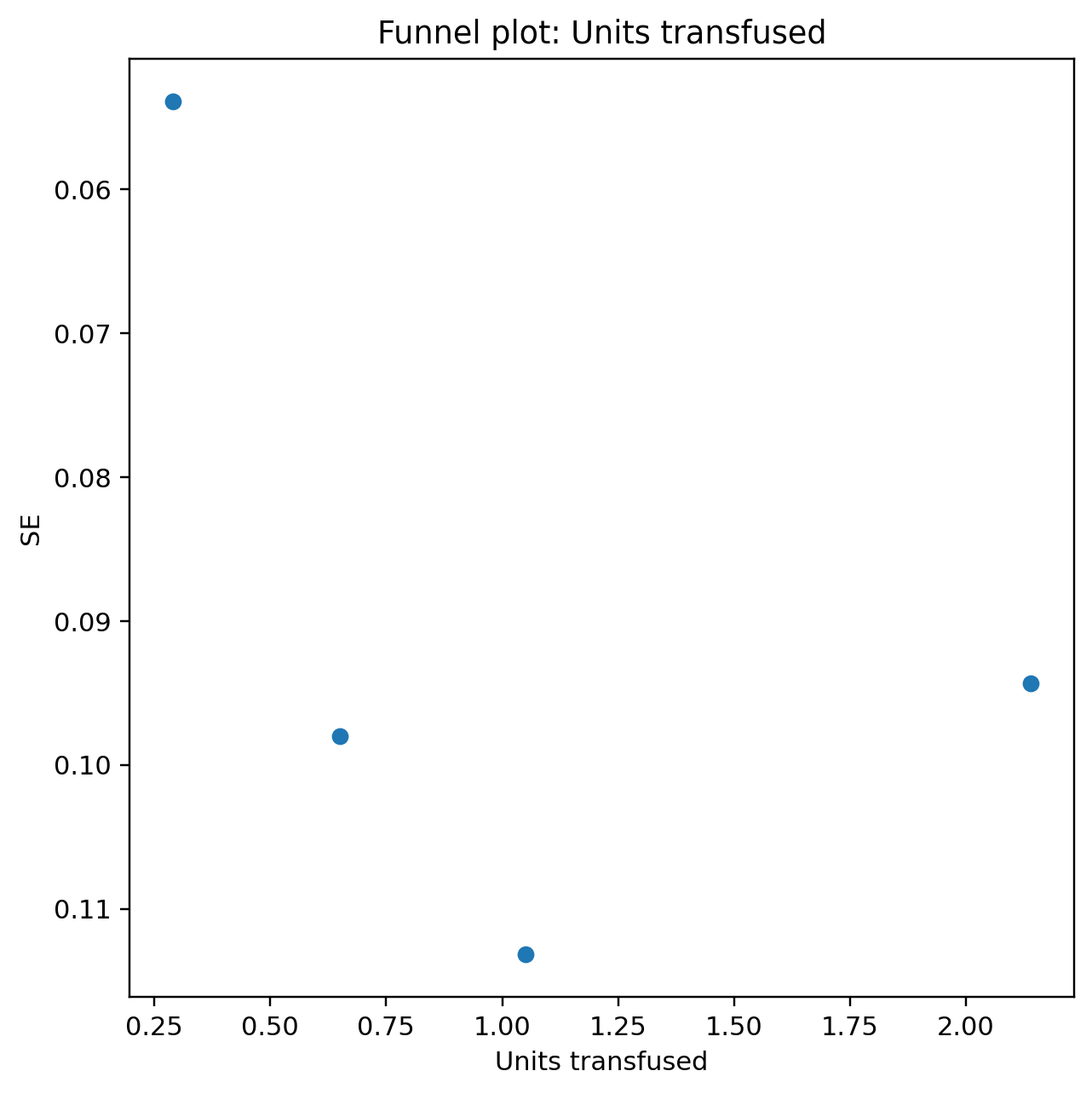

Supplement: Supplementary file 15 — Supplementary Figure 15. Funnel plot: Autologous predonation ‐ Units transfused. Funnel plot of studies reporting units transfused per patient. The sparse distribution and small sample size limit interpretability; publication bias cannot be assessed. [file JEO2-13-e70867-s040.png]

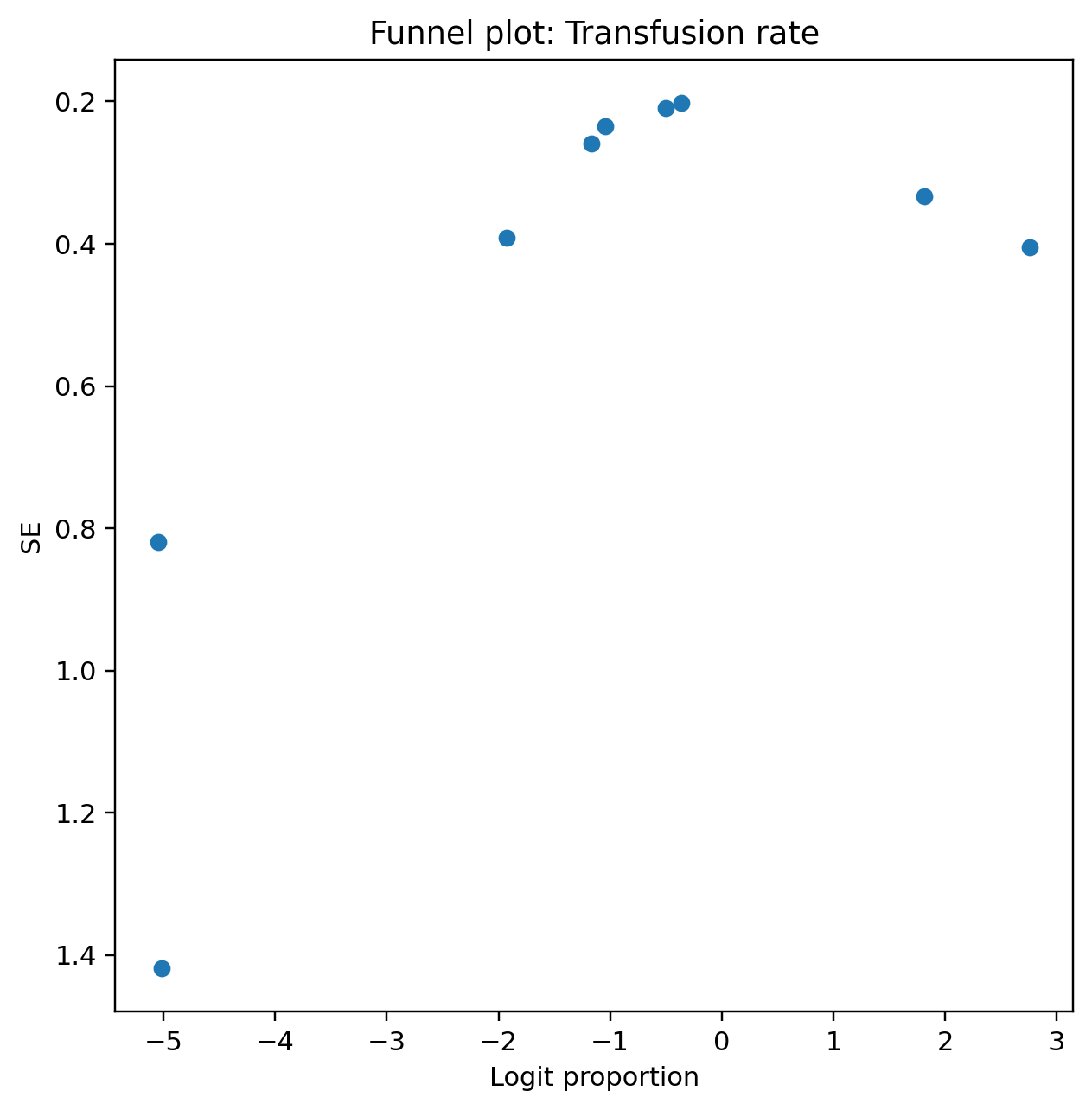

Supplement: Supplementary file 16 — Supplementary Figure 16. Funnel plot: Autologous predonation ‐ Transfusion rate. Funnel plot of studies reporting transfusion rate. The low number of included studies and uneven distribution prevent reliable interpretation of small‐study effects. [file JEO2-13-e70867-s015.png]

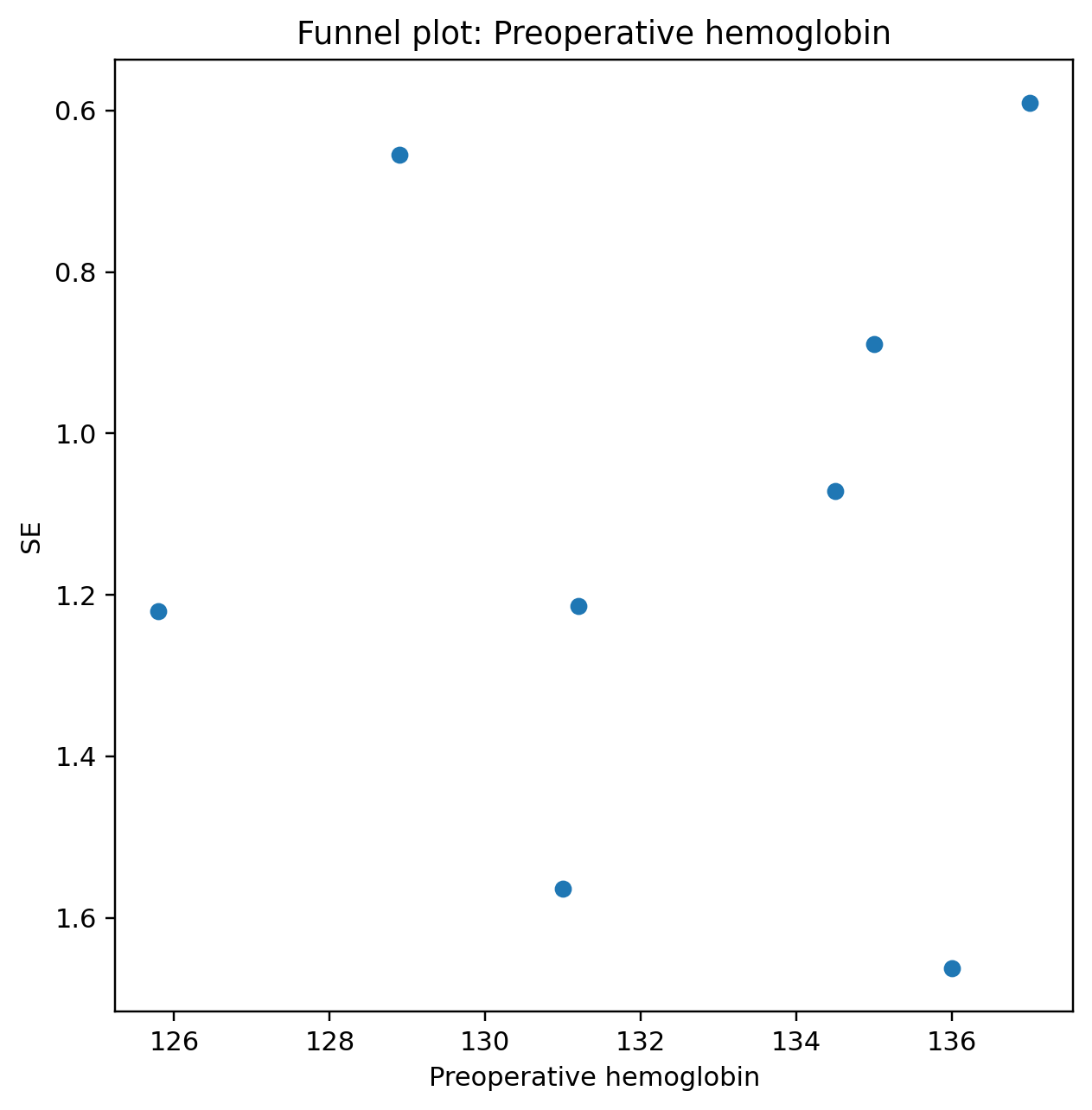

Supplement: Supplementary file 17 — Supplementary Figure 17. Funnel plot: Autologous predonation ‐ Preoperative hemoglobin. Funnel plot of studies reporting preoperative hemoglobin levels. Interpretation is limited by the small number of studies and apparent asymmetry. [file JEO2-13-e70867-s023.png]

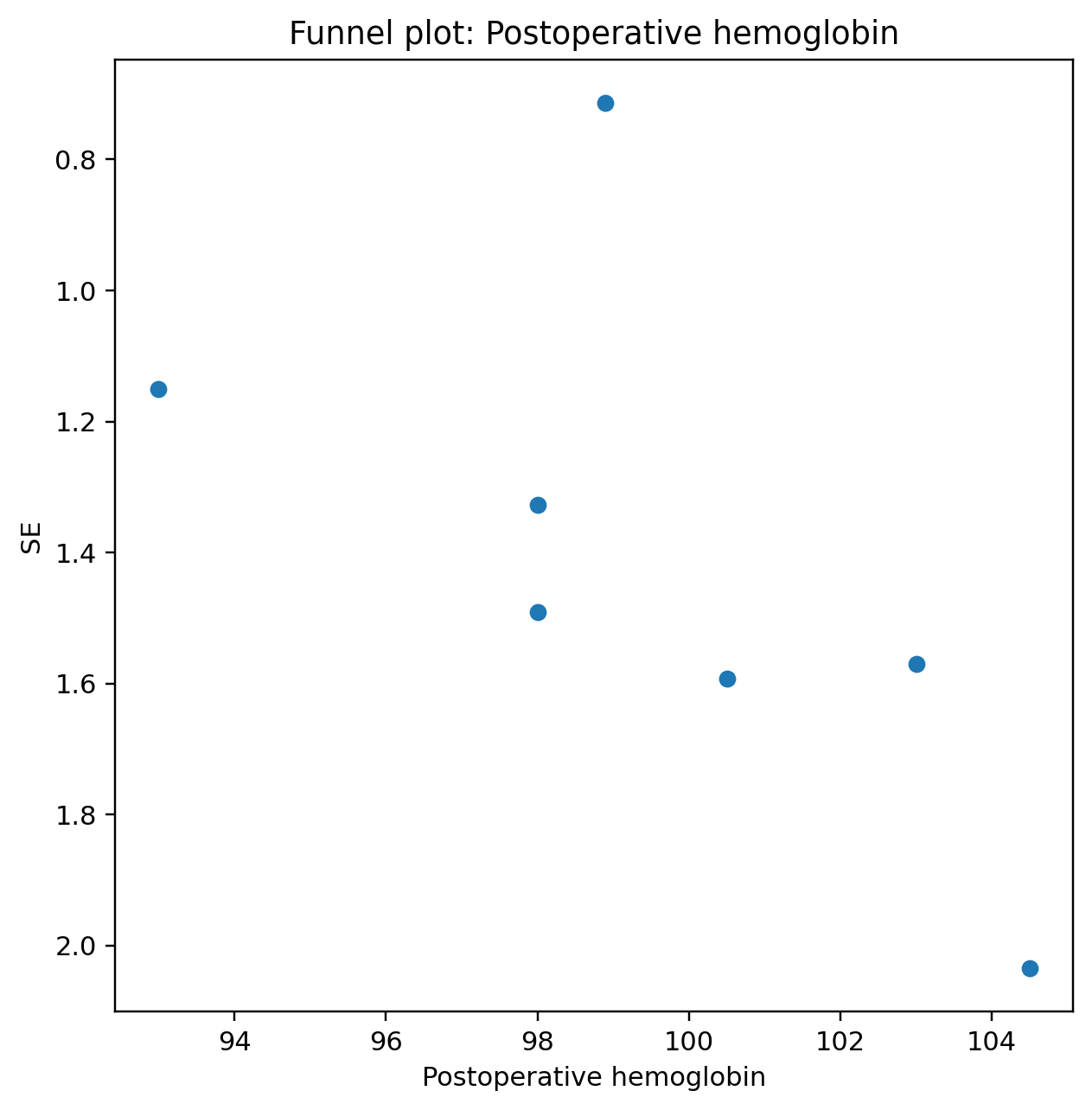

Supplement: Supplementary file 18 — Supplementary Figure 18. Funnel plot: Autologous predonation ‐ Postoperative hemoglobin. Funnel plot of studies reporting postoperative hemoglobin levels. The limited dataset precludes meaningful assessment of publication bias. [file JEO2-13-e70867-s041.png]

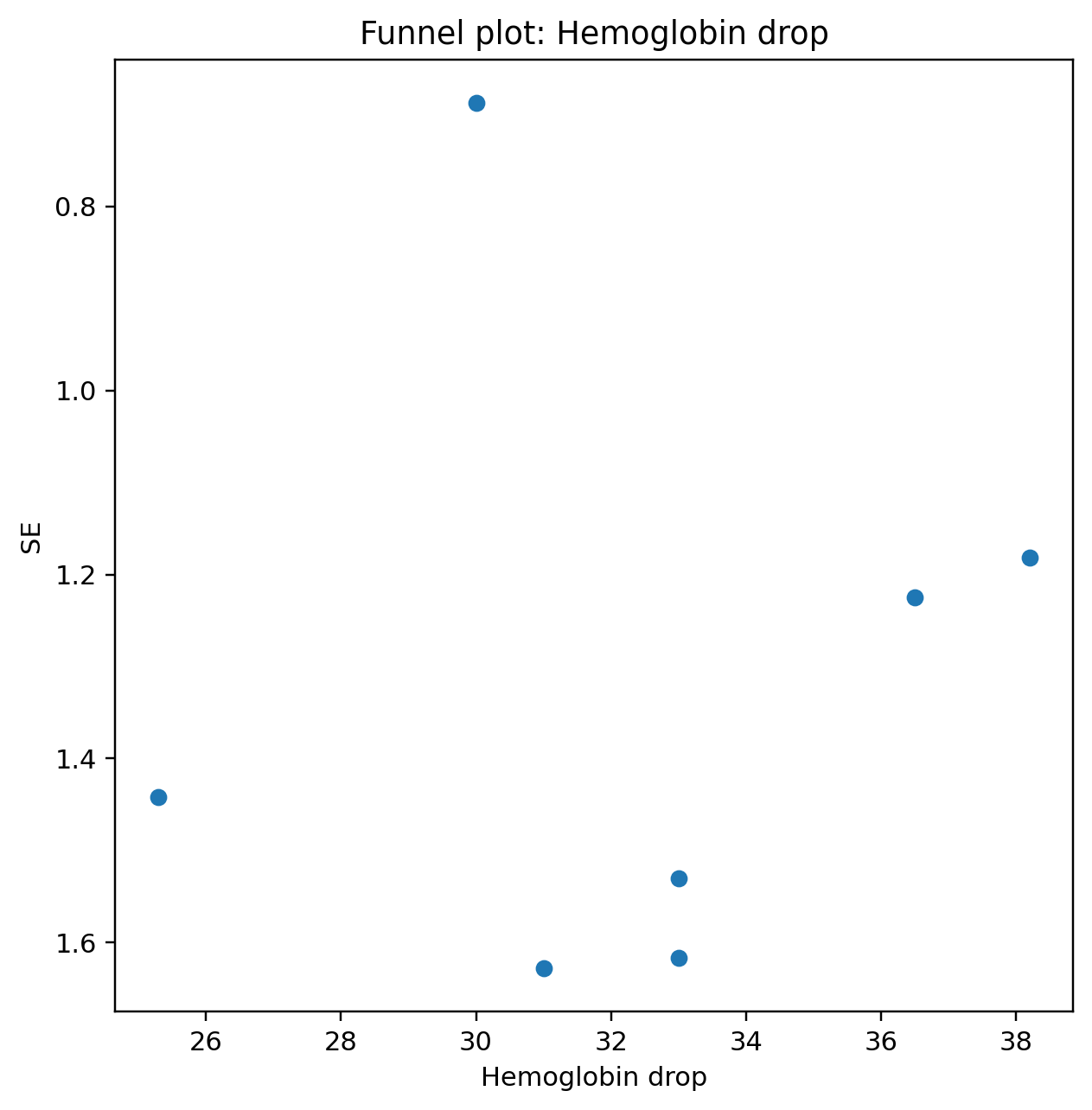

Supplement: Supplementary file 19 — Supplementary Figure 19. Funnel plot: Autologous predonation ‐ Hemoglobin drop. Funnel plot of studies reporting hemoglobin drop. Due to the small number of studies, conclusions regarding symmetry or small‐study effects are not reliable. [file JEO2-13-e70867-s009.png]

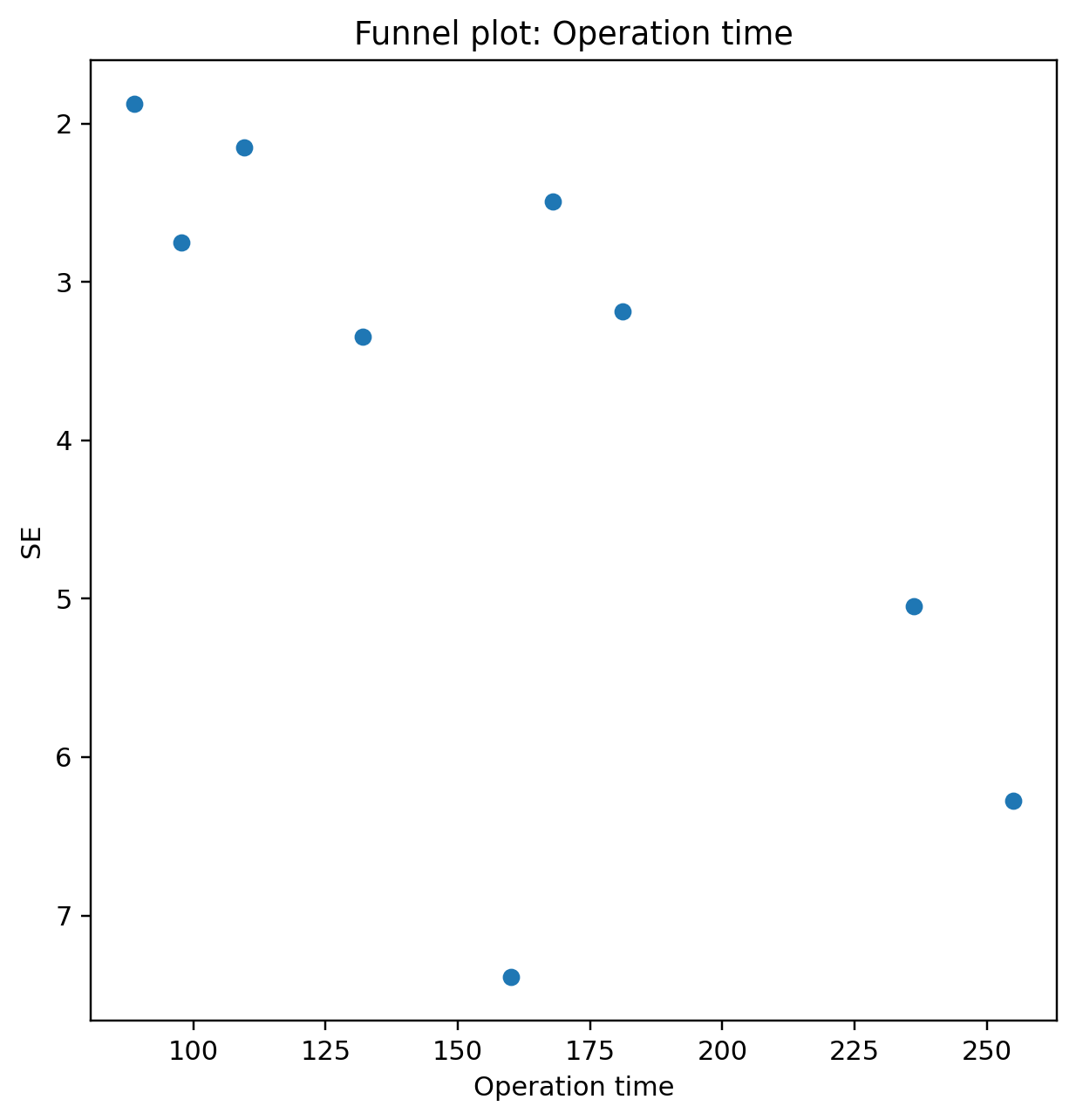

Supplement: Supplementary file 20 — Supplementary Figure 20. Funnel plot: Autologous predonation ‐ Operation time. Funnel plot of studies reporting operation time. The small number of data points limits interpretability and prevents meaningful conclusions regarding publication bias. [file JEO2-13-e70867-s043.png]

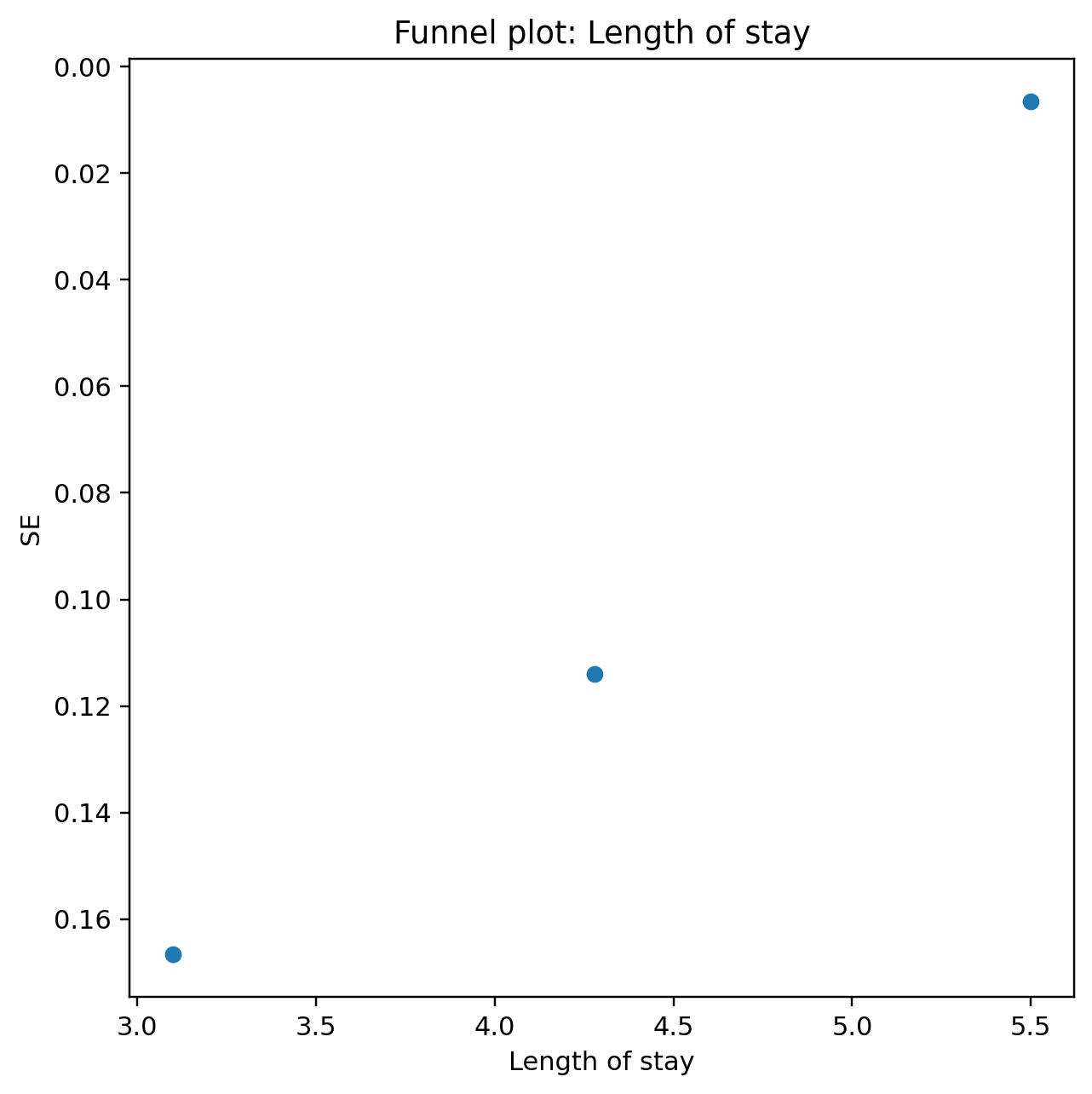

Supplement: Supplementary file 21 — Supplementary Figure 21. Funnel plot: Autologous predonation ‐ Length of stay. Funnel plot of studies reporting length of stay. Interpretation is limited due to the very small number of included studies. [file JEO2-13-e70867-s008.png]

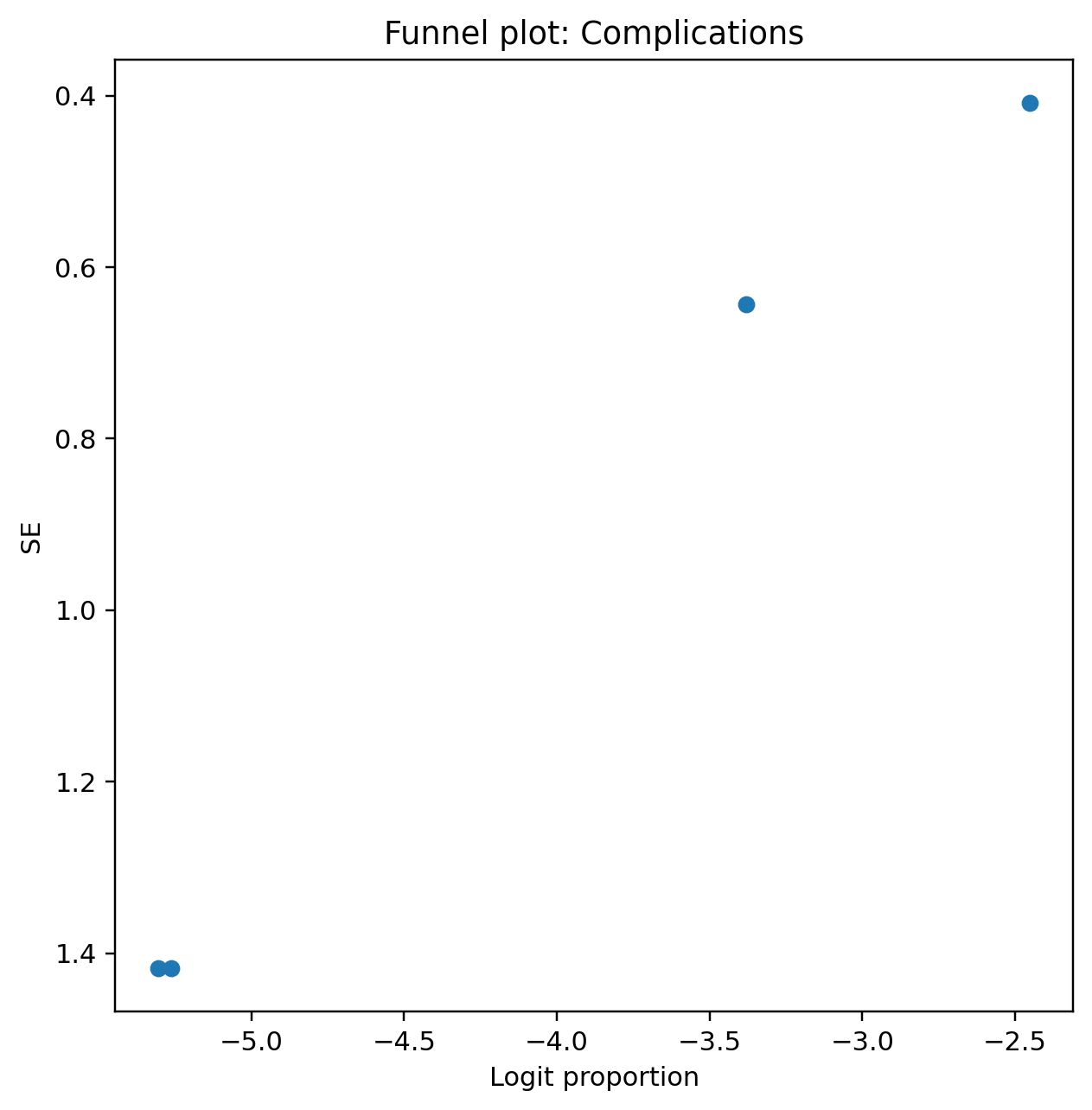

Supplement: Supplementary file 22 — Supplementary Figure 22. Funnel plot: Autologous predonation – Complications. Funnel plot of studies reporting complications. The sparse data do not allow reliable assessment of funnel plot symmetry. [file JEO2-13-e70867-s021.png]

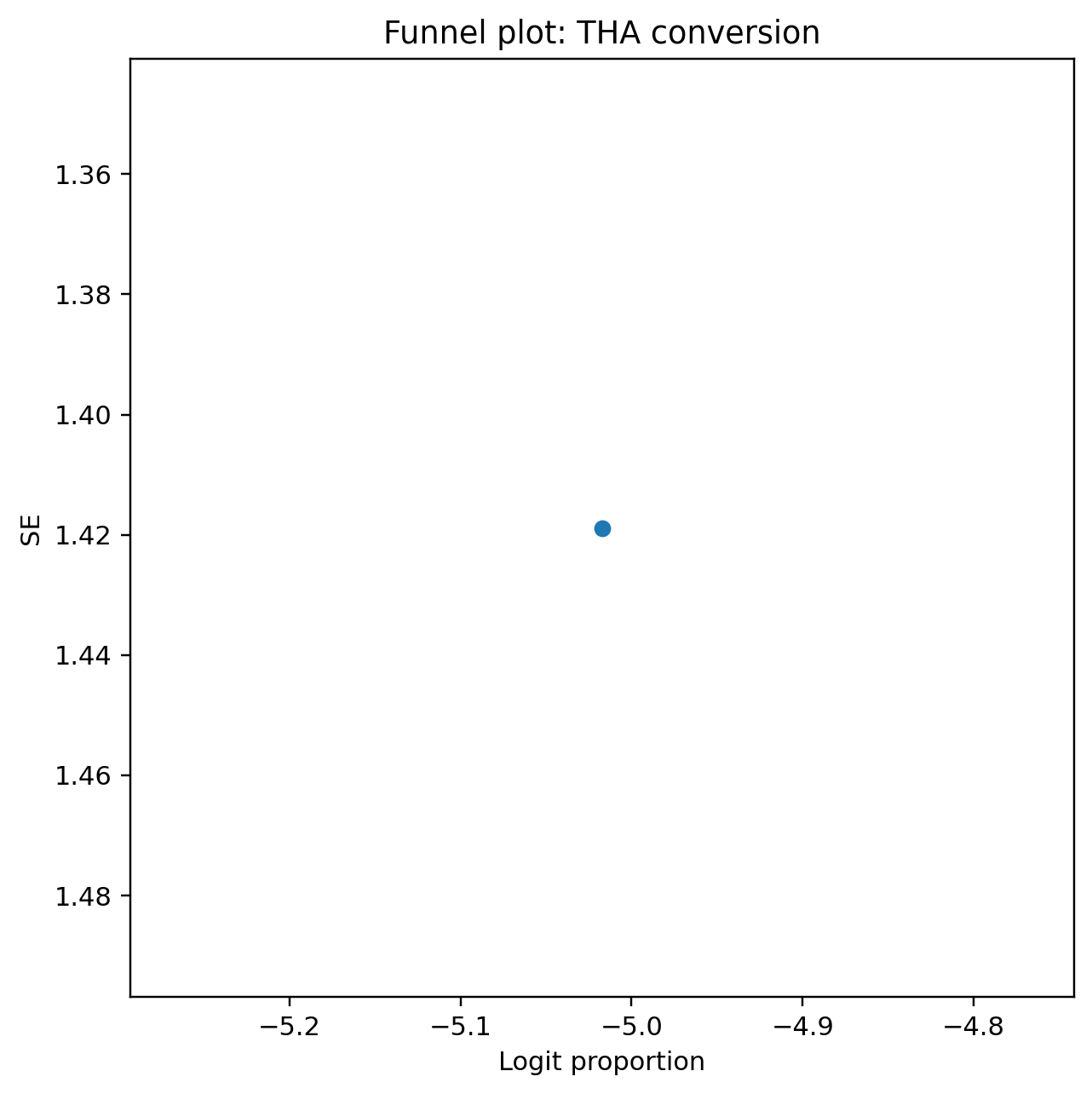

Supplement: Supplementary file 23 — Supplementary Figure 23. Funnel plot: Autologous predonation ‐ THA conversion. Funnel plot of studies reporting conversion to total hip arthroplasty. With only minimal data available, no conclusions regarding publication bias can be drawn. [file JEO2-13-e70867-s014.png]

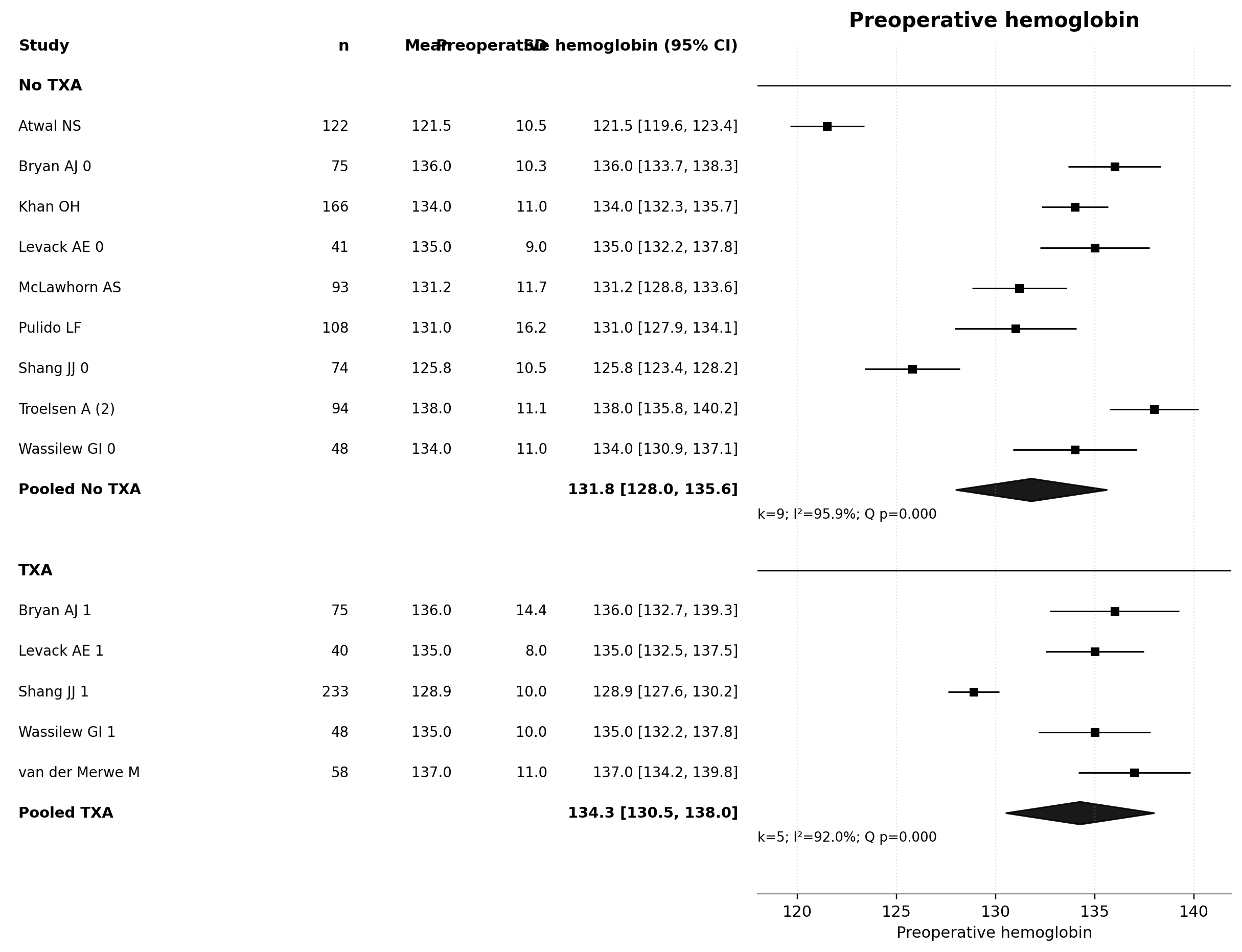

Supplement: Supplementary file 24 — Supplementary Figure 24. Forest plot: TXA Preoperative hemoglobin. Forest plot of the arm‐based multilevel random‐effects meta‐analysis comparing preoperative hemoglobin levels between TXA and non‐TXA groups. No statistically significant difference was observed. Effect sizes are presented as mean differences with 95% confidence intervals. [file JEO2-13-e70867-s044.png]

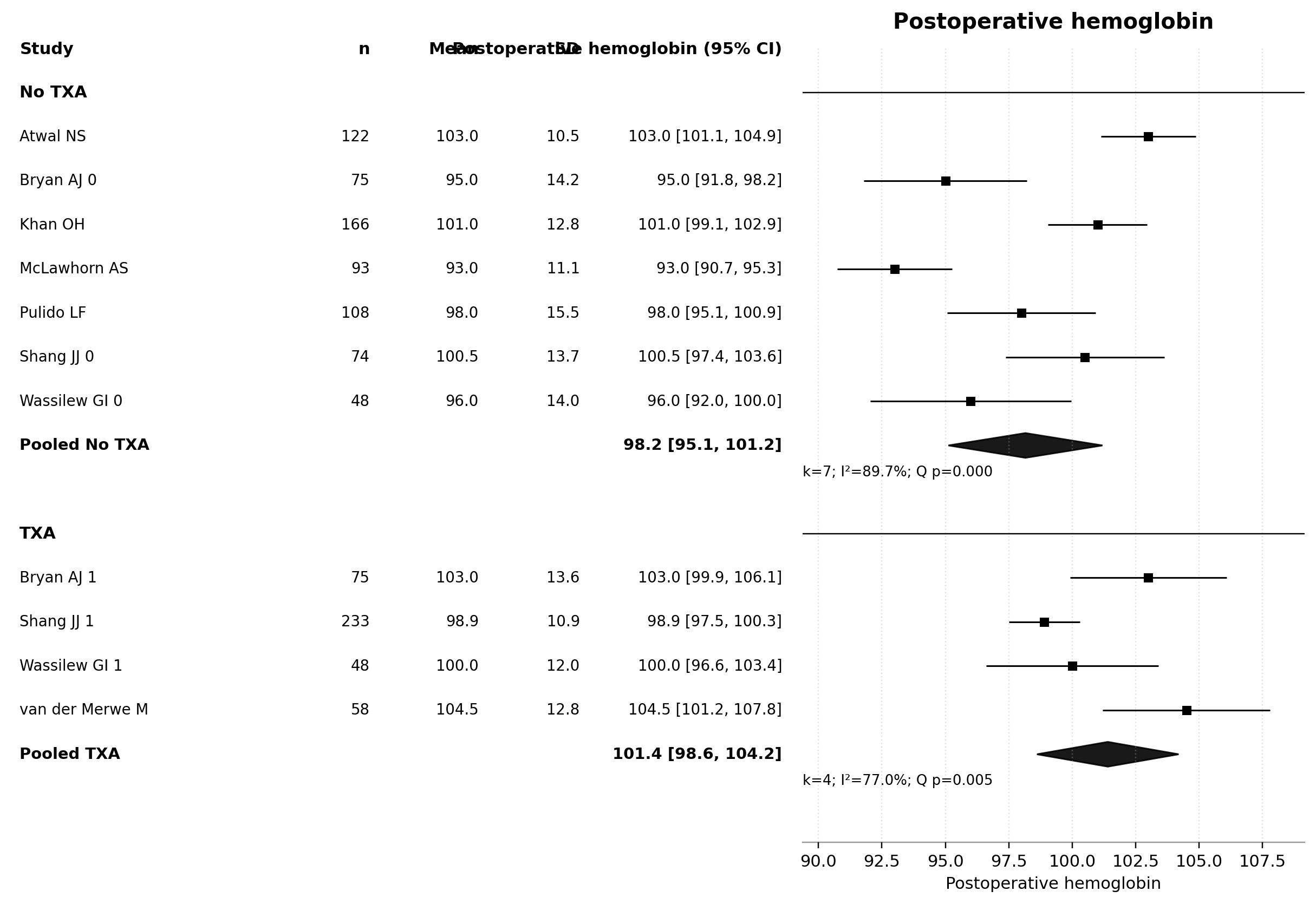

Supplement: Supplementary file 25 — Supplementary Figure 25. Forest plot: TXA ‐ Postoperative hemoglobin. Forest plot of the arm‐based multilevel random‐effects meta‐analysis comparing postoperative hemoglobin levels between TXA and non‐TXA groups. No statistically significant difference was observed. Effect sizes are presented as mean differences with 95% confidence intervals. [file JEO2-13-e70867-s035.png]

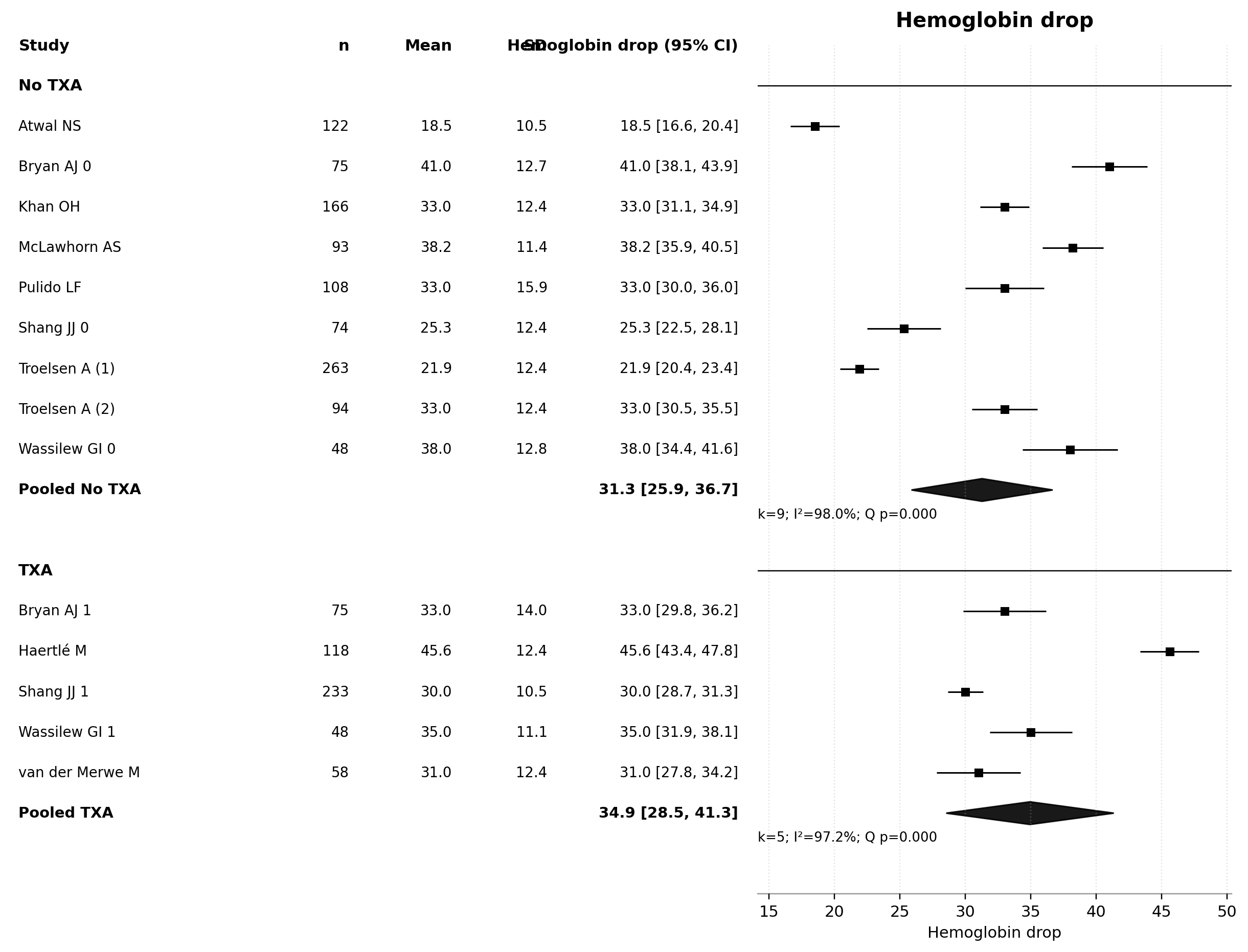

Supplement: Supplementary file 26 — Supplementary Figure 26. Forest plot: TXA ‐ Hemoglobin drop. Forest plot of the arm‐based multilevel random‐effects meta‐analysis comparing hemoglobin decrease between TXA and non‐TXA groups. No statistically significant difference was observed. Effect sizes are presented as mean differences with 95% confidence intervals. [file JEO2-13-e70867-s020.png]

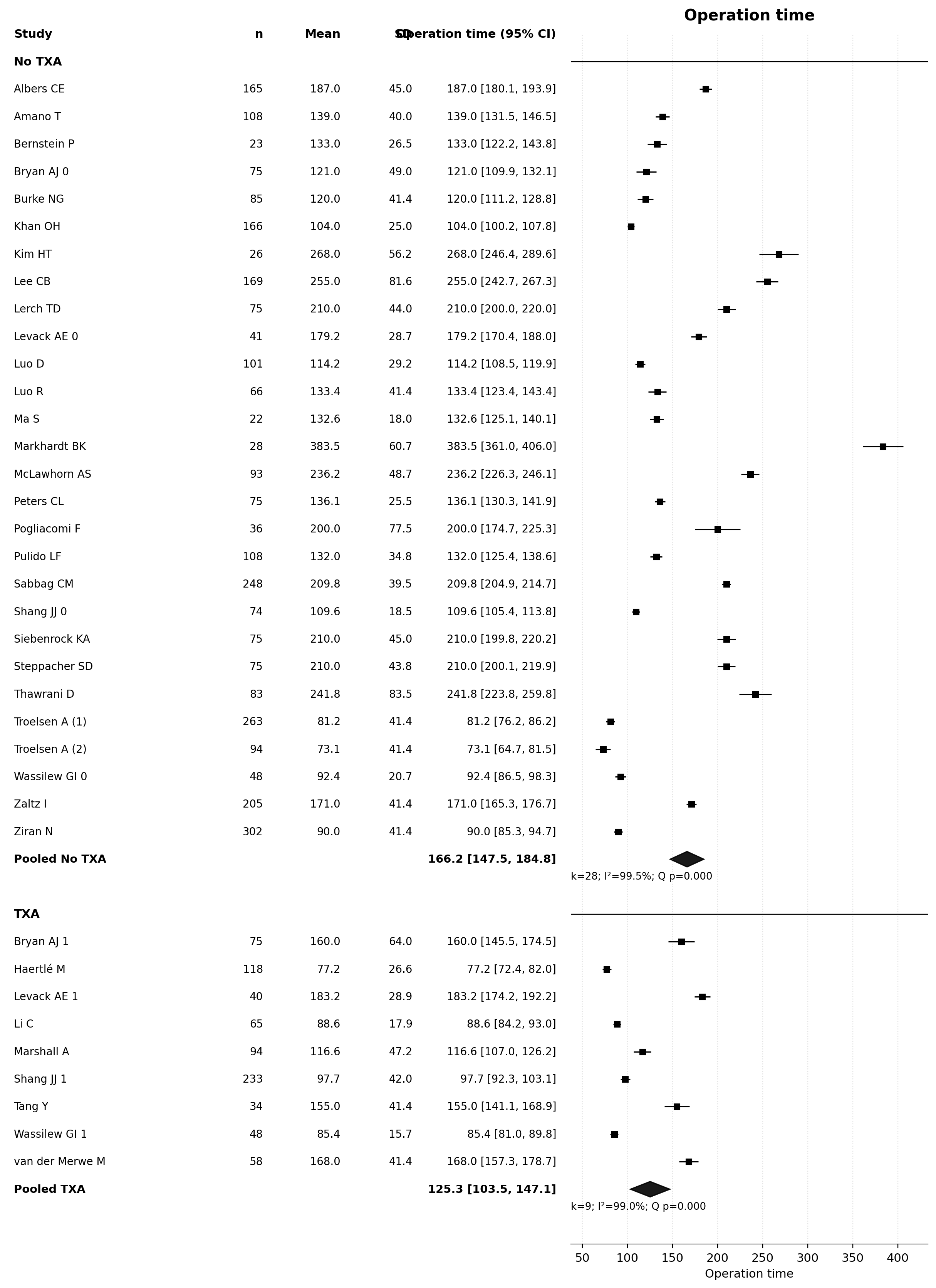

Supplement: Supplementary file 27 — Supplementary Figure 27. Forest plot: TXA ‐ Operation time. Forest plot of the arm‐based multilevel random‐effects meta‐analysis comparing operation time between TXA and non‐TXA groups. No statistically significant difference was observed. Effect sizes are presented as mean differences with 95% confidence intervals. [file JEO2-13-e70867-s028.png]

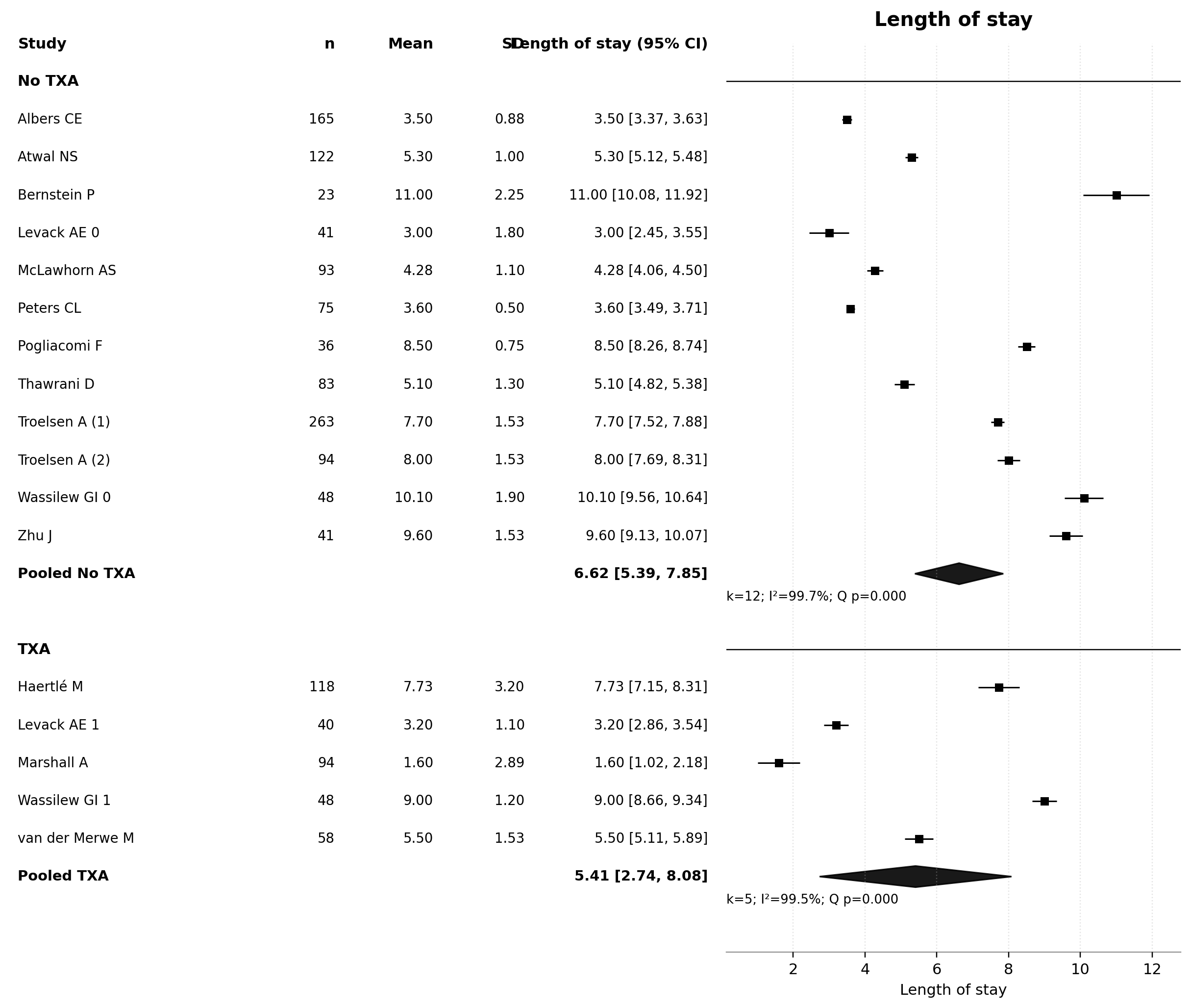

Supplement: Supplementary file 28 — Supplementary Figure 28. Forest plot: TXA ‐ Length of stay. Forest plot of the arm‐based multilevel random‐effects meta‐analysis comparing length of hospital stay between TXA and non‐TXA groups. No statistically significant difference was observed. Effect sizes are presented as mean differences with 95% confidence intervals. [file JEO2-13-e70867-s037.png]

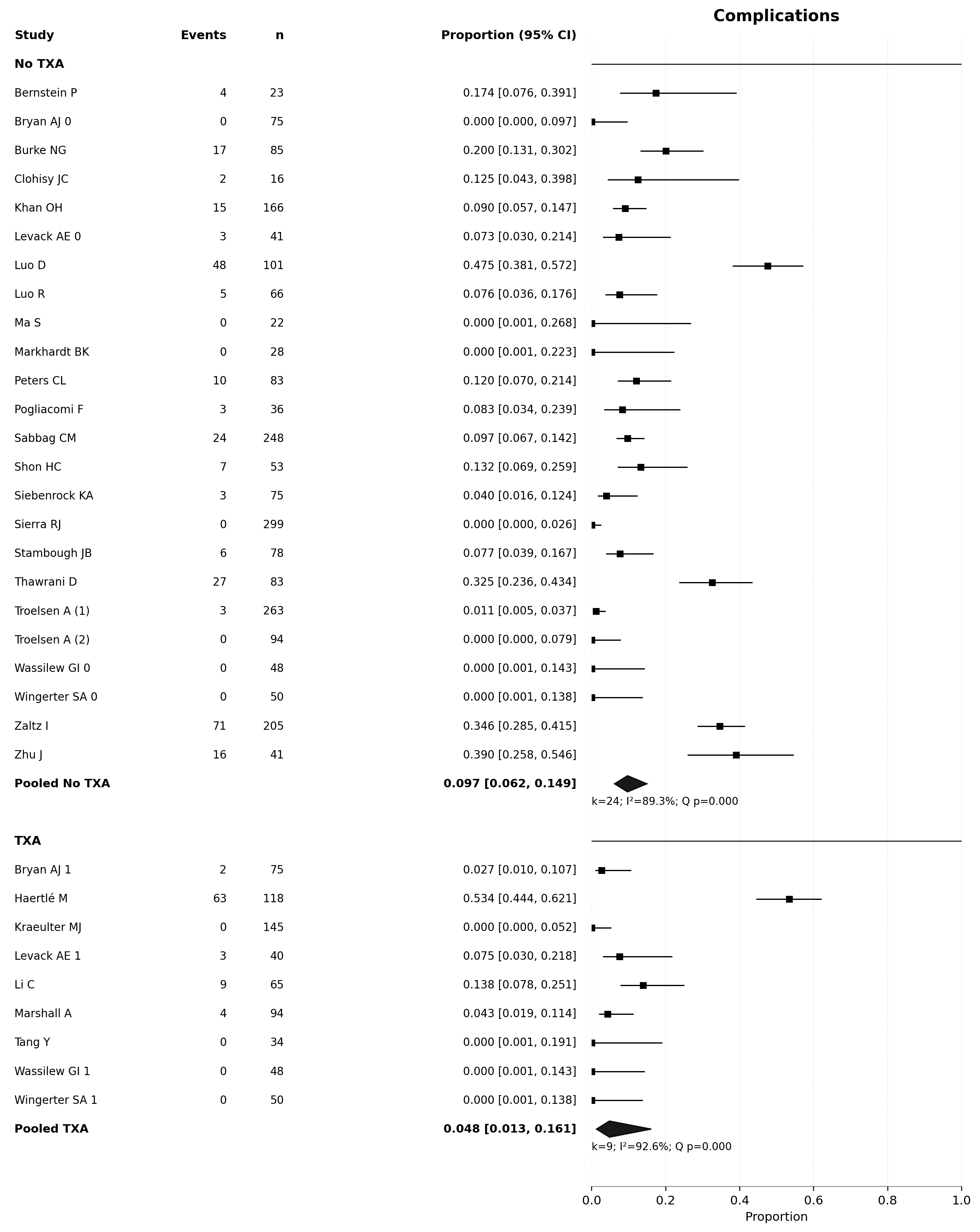

Supplement: Supplementary file 29 — Supplementary Figure 29. Forest plot: TXA ‐ Complications. Forest plot of the arm‐based multilevel random‐effects meta‐analysis comparing complication rates between TXA and non‐TXA groups. No statistically significant difference was observed. Effect sizes are presented as odds ratios with 95% confidence intervals. [file JEO2-13-e70867-s002.png]

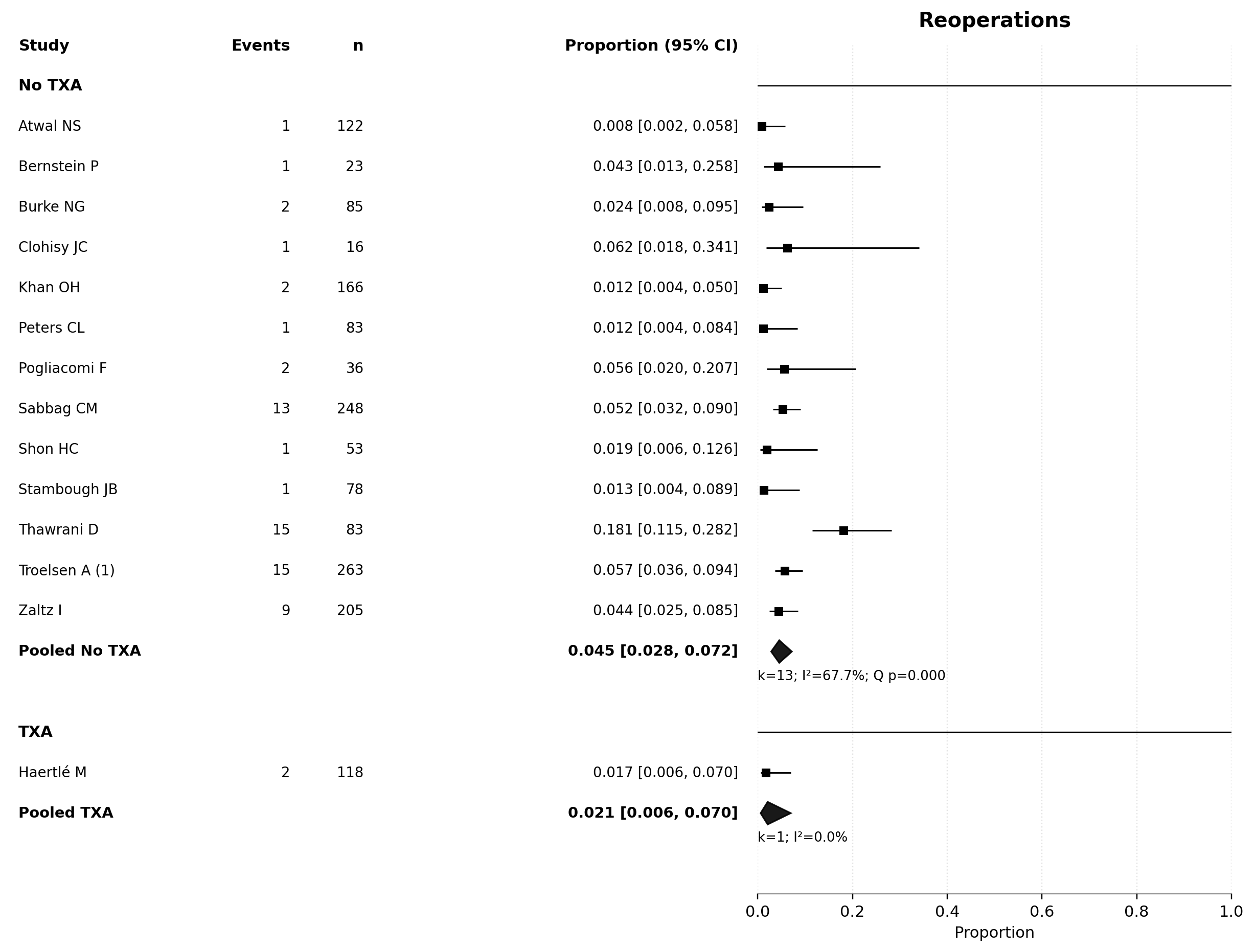

Supplement: Supplementary file 30 — Supplementary Figure 30. Forest plot: TXA ‐ Reoperations. Forest plot of the arm‐based multilevel random‐effects meta‐analysis comparing reoperation rates between TXA and non‐TXA groups. No statistically significant difference was observed. Effect sizes are presented as odds ratios with 95% confidence intervals. [file JEO2-13-e70867-s039.png]

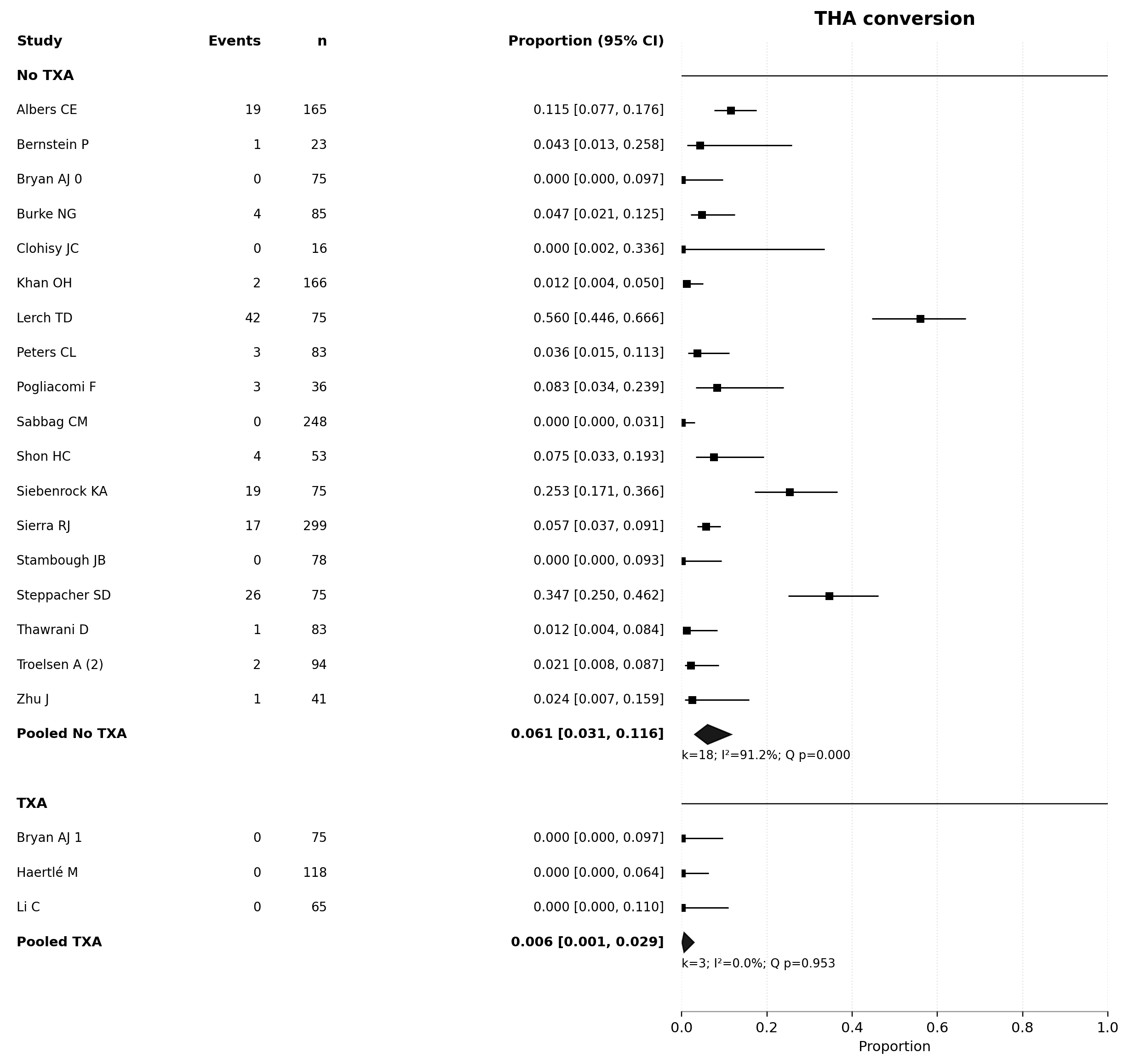

Supplement: Supplementary file 31 — Supplementary Figure 31. Forest plot: TXA ‐ THA conversion. Forest plot of the arm‐based multilevel random‐effects meta‐analysis comparing conversion to total hip arthroplasty (THA) between TXA and non‐TXA groups. No statistically significant difference was observed. Effect sizes are presented as odds ratios with 95% confidence intervals. [file JEO2-13-e70867-s011.png]

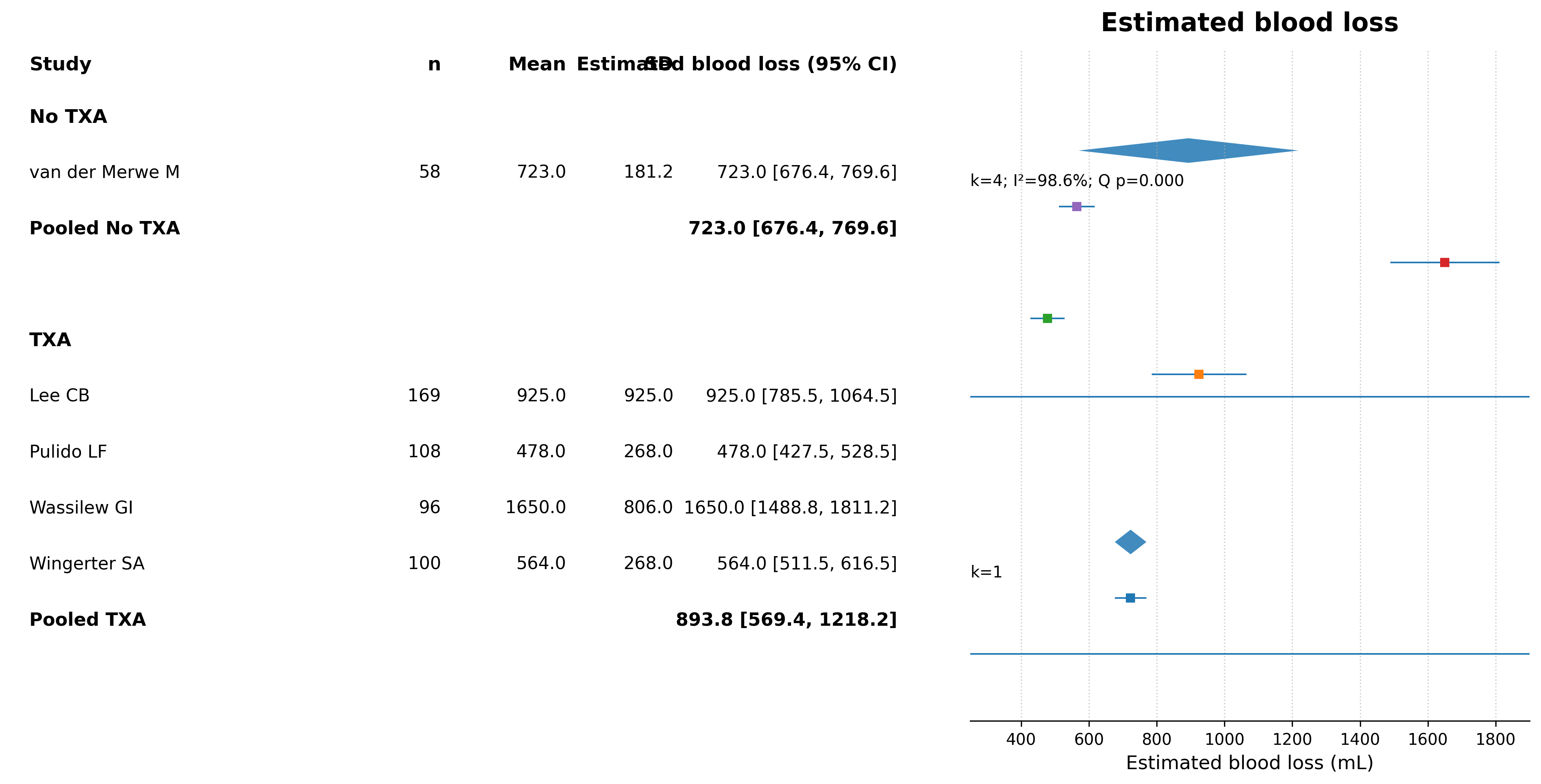

Supplement: Supplementary file 32 — Supplementary Figure 32. Forest plot: Autologous predonation – estimated blood loss. Forest plot of the arm‐based multilevel random‐effects meta‐analysis comparing estimated blood loss between predonation and no‐predonation groups. No statistically significant difference was observed. Effect sizes are presented as mean differences with 95% confidence intervals. [file JEO2-13-e70867-s024.png]

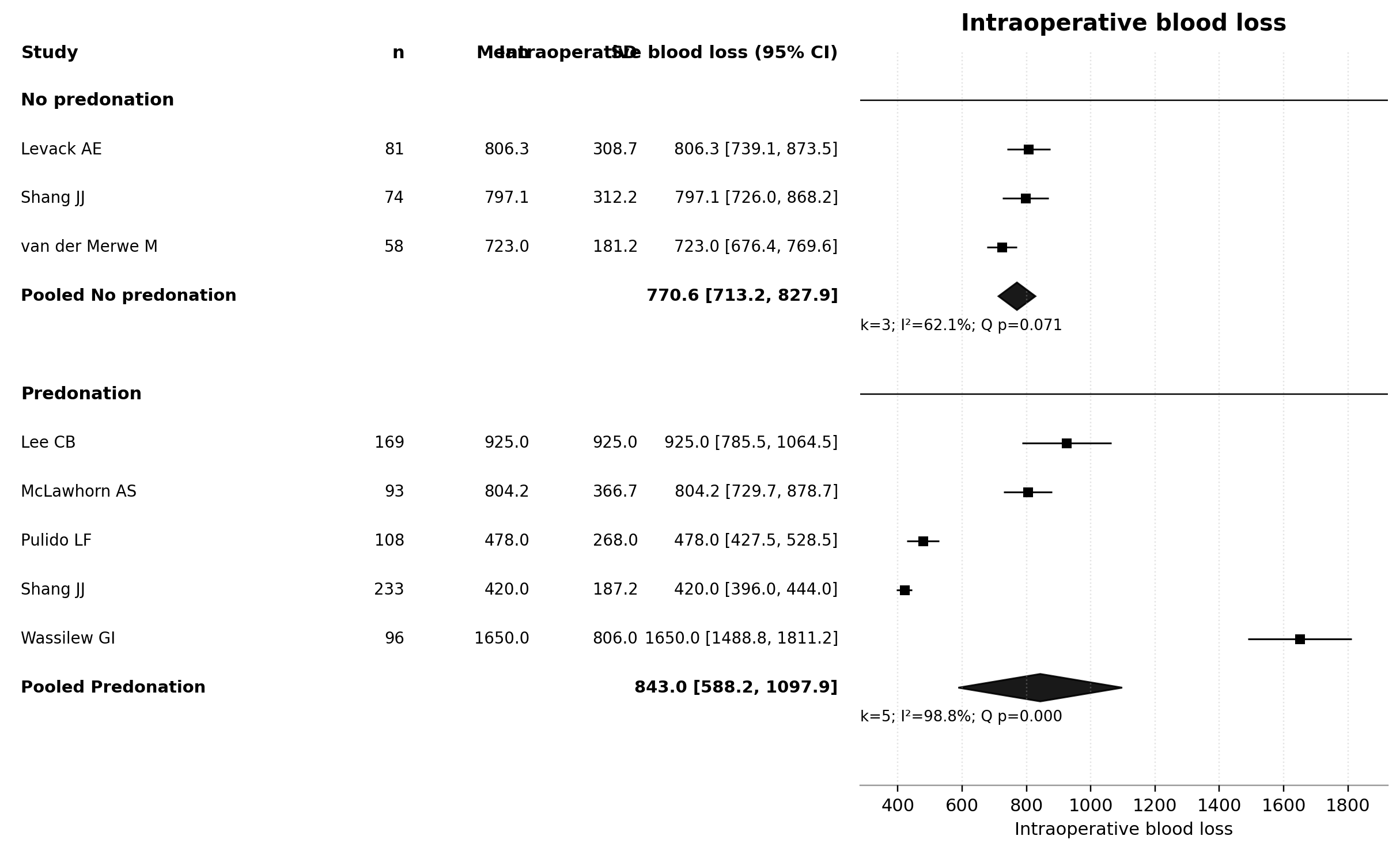

Supplement: Supplementary file 33 — Supplementary Figure 33. Forest plot: Autologous predonation – intraoperative blood loss. Forest plot of the arm‐based multilevel random‐effects meta‐analysis comparing intraoperative blood loss between predonation and no‐predonation groups. No statistically significant difference was observed. Effect sizes are presented as mean differences with 95% confidence intervals. [file JEO2-13-e70867-s013.png]

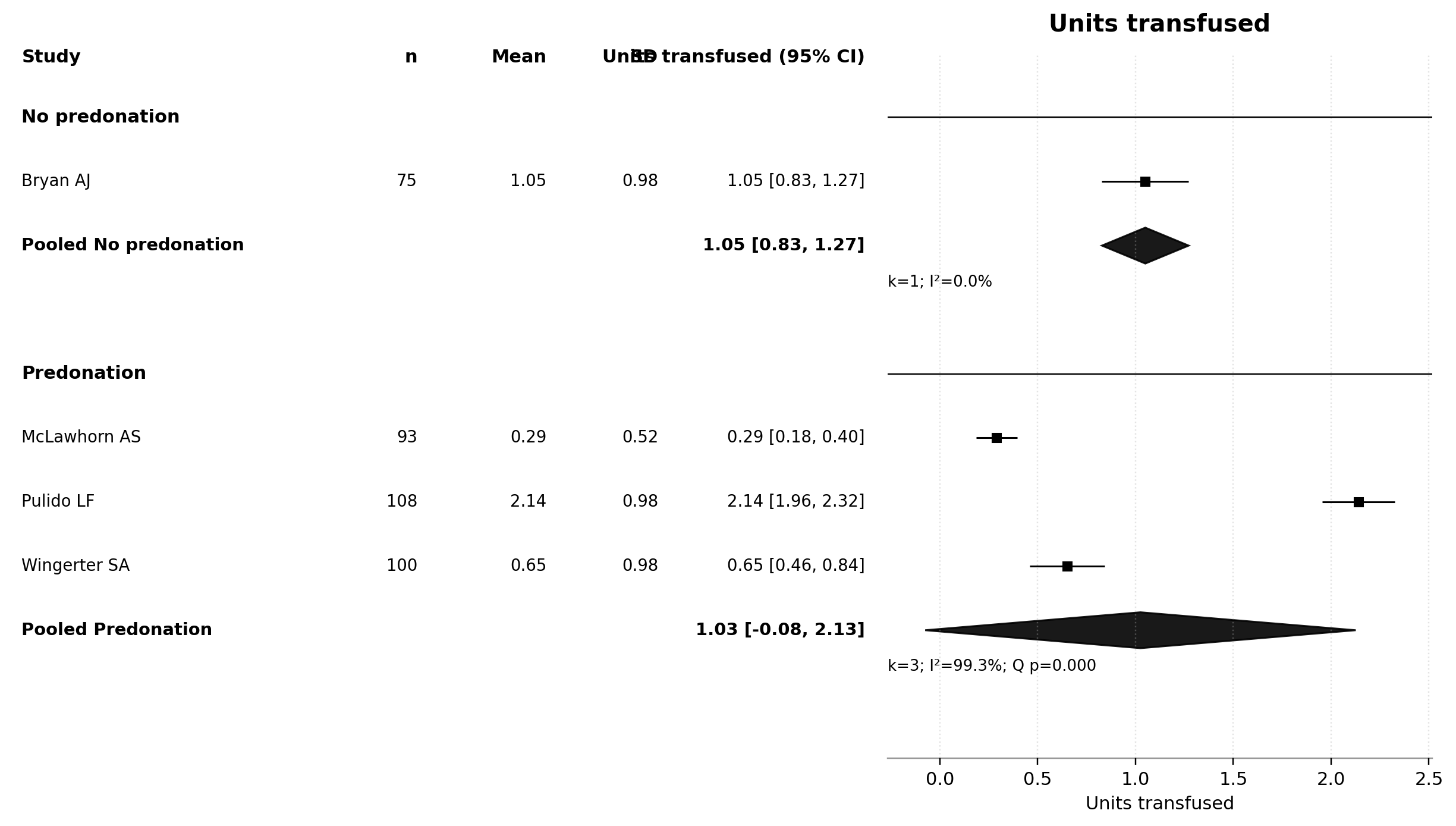

Supplement: Supplementary file 34 — Supplementary Figure 34. Forest plot: Autologous predonation – units transfused. Forest plot of the arm‐based multilevel random‐effects meta‐analysis comparing the number of transfused units per patient between predonation and no‐predonation groups. No statistically significant difference was observed. Effect sizes are presented as mean differences with 95% confidence intervals. [file JEO2-13-e70867-s005.png]

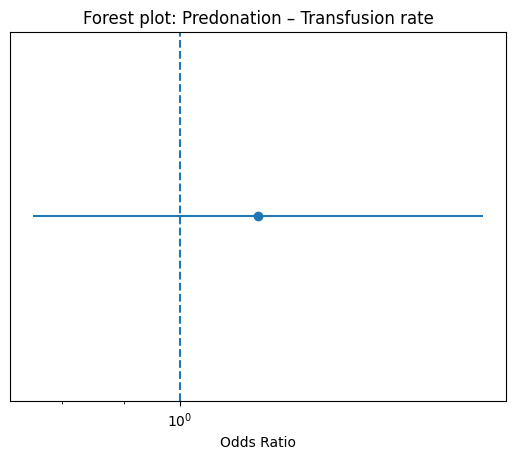

Supplement: Supplementary file 35 — Supplementary Figure 35. Forest plot: Autologous predonation – transfusion rate. Forest plot of the arm‐based multilevel random‐effects meta‐analysis comparing transfusion rates between predonation and no‐predonation groups. No statistically significant difference was observed. Effect sizes are presented as odds ratios with 95% confidence intervals. [file JEO2-13-e70867-s033.png]

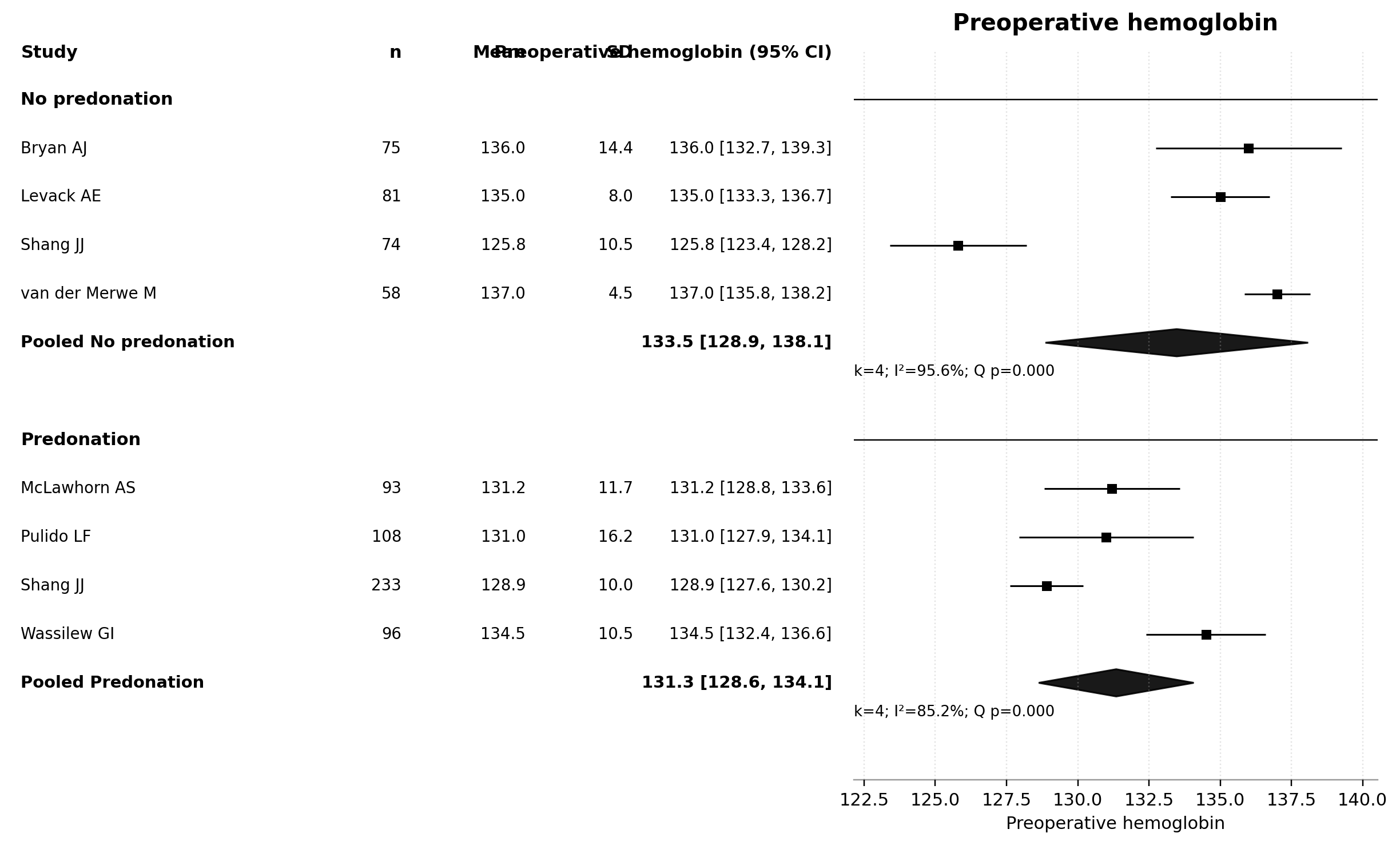

Supplement: Supplementary file 36 — Supplementary Figure 36. Forest plot: Autologous predonation – preoperative hemoglobin. Forest plot of the arm‐based multilevel random‐effects meta‐analysis comparing preoperative hemoglobin levels between predonation and no‐predonation groups. No statistically significant difference was observed. Effect sizes are presented as mean differences with 95% confidence intervals. [file JEO2-13-e70867-s018.png]

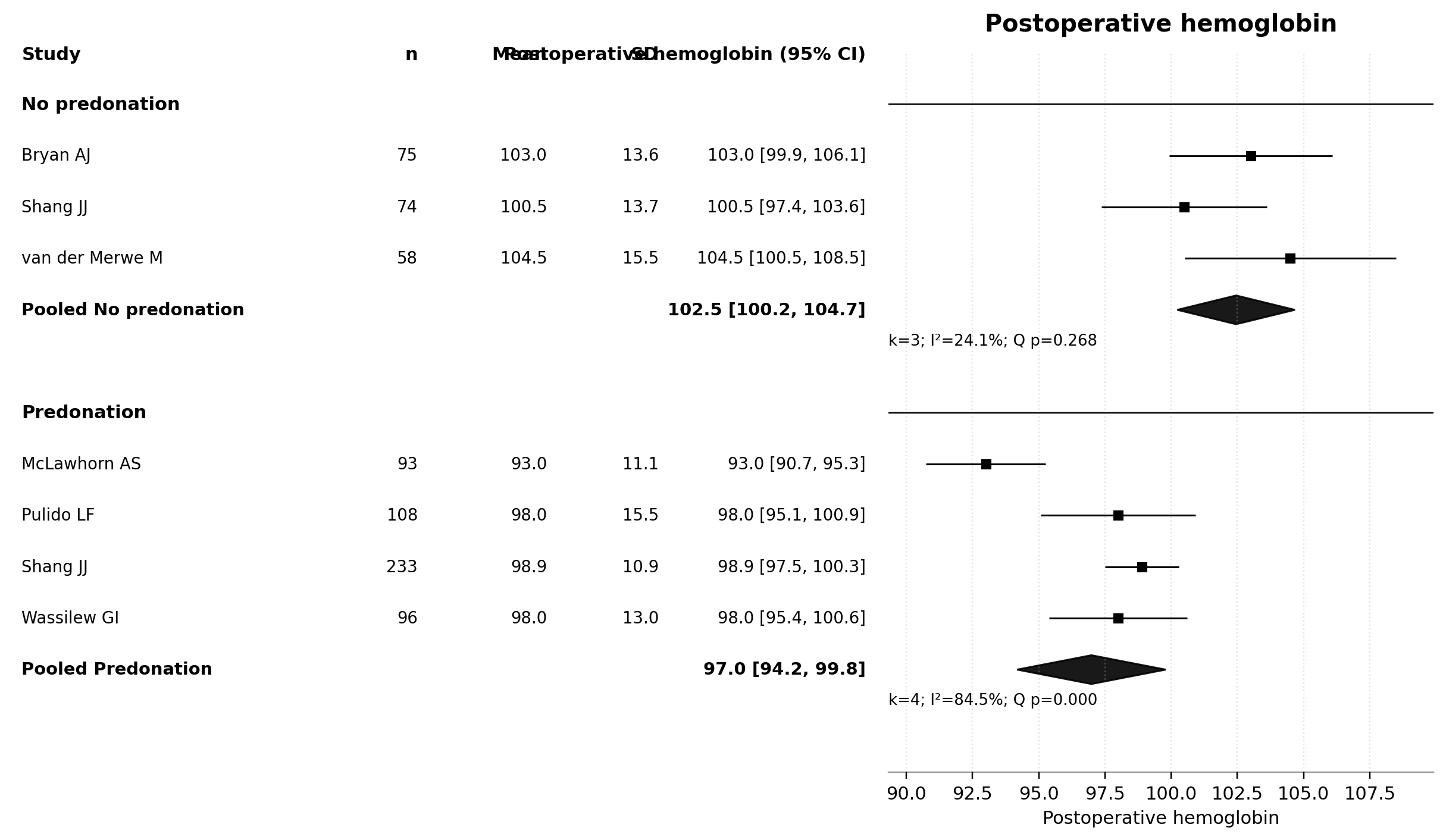

Supplement: Supplementary file 37 — Supplementary Figure 37. Forest plot: Autologous predonation – postoperative hemoglobin. Forest plot of the arm‐based multilevel random‐effects meta‐analysis comparing postoperative hemoglobin levels between predonation and no‐predonation groups. Predonation was associated with significantly lower postoperative hemoglobin. Effect sizes are presented as mean differences with 95% confidence intervals. [file JEO2-13-e70867-s030.png]

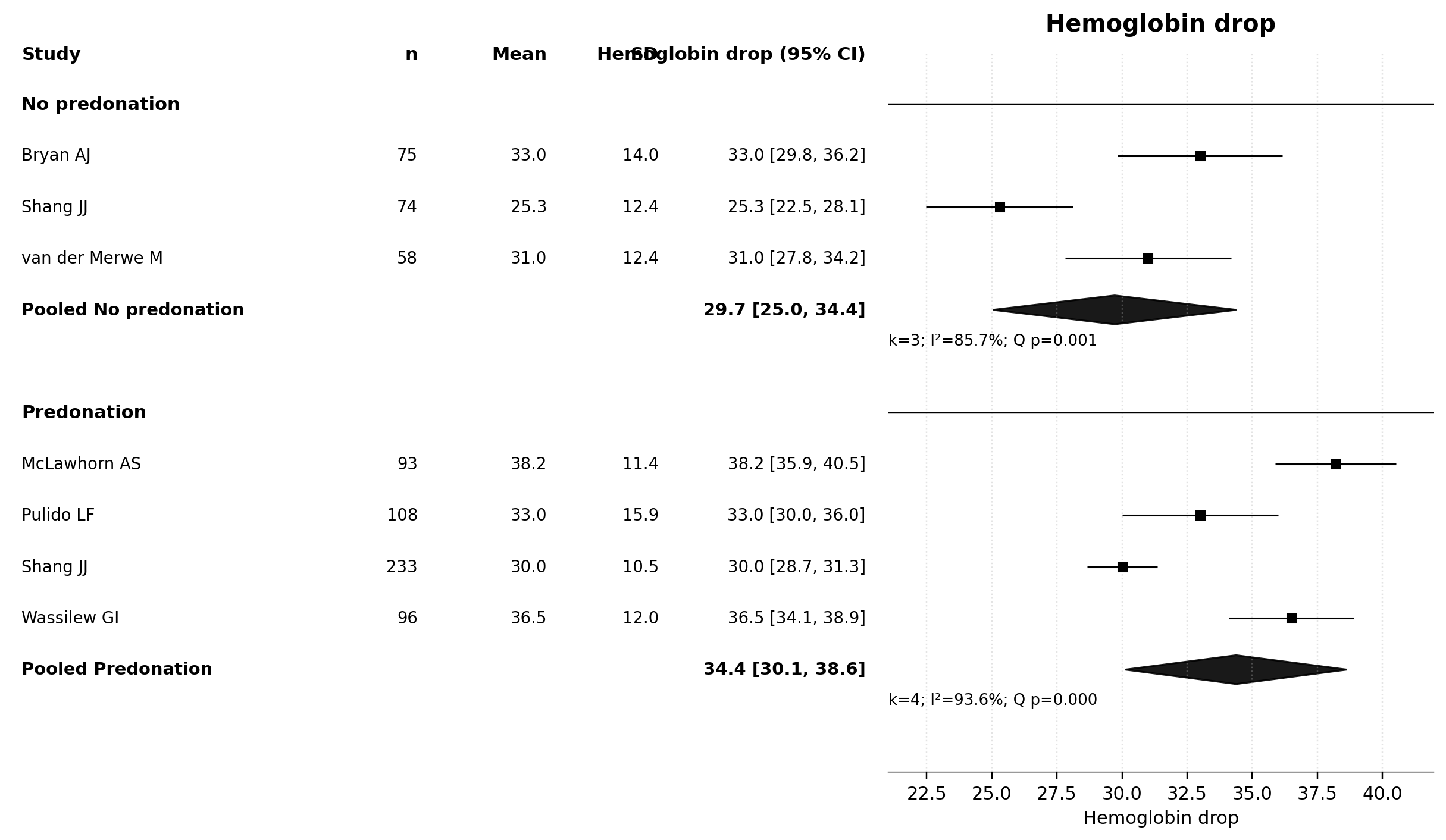

Supplement: Supplementary file 38 — Supplementary Figure 38. Forest plot: Autologous predonation – hemoglobin drop. Forest plot of the arm‐based multilevel random‐effects meta‐analysis comparing hemoglobin decrease between predonation and no‐predonation groups. No statistically significant difference was observed. Effect sizes are presented as mean differences with 95% confidence intervals. [file JEO2-13-e70867-s031.png]

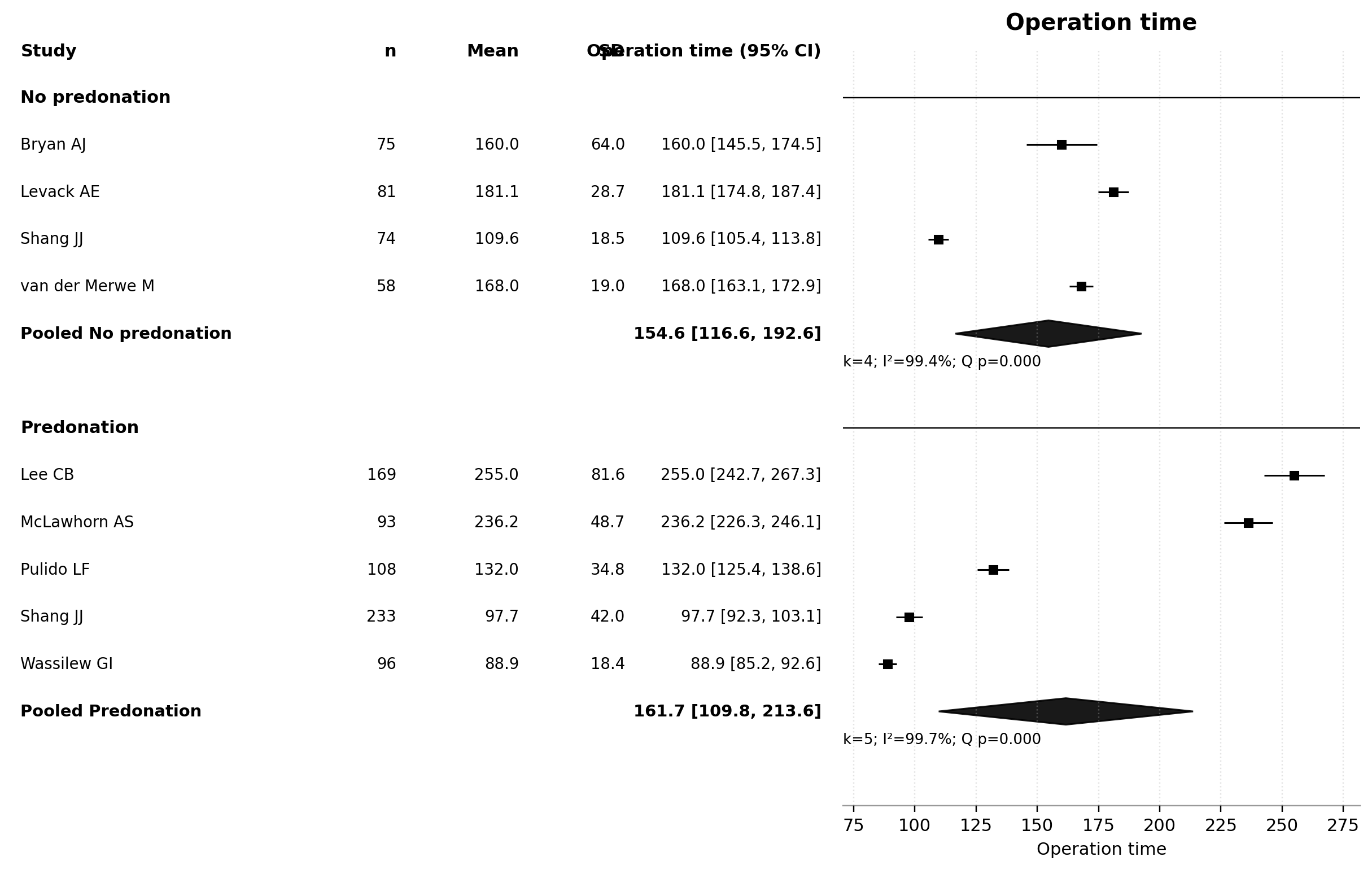

Supplement: Supplementary file 39 — Supplementary Figure 39. Forest plot: Autologous predonation – operation time. Forest plot of the arm‐based multilevel random‐effects meta‐analysis comparing operation time between predonation and no‐predonation groups. No statistically significant difference was observed. Effect sizes are presented as mean differences with 95% confidence intervals. [file JEO2-13-e70867-s003.png]

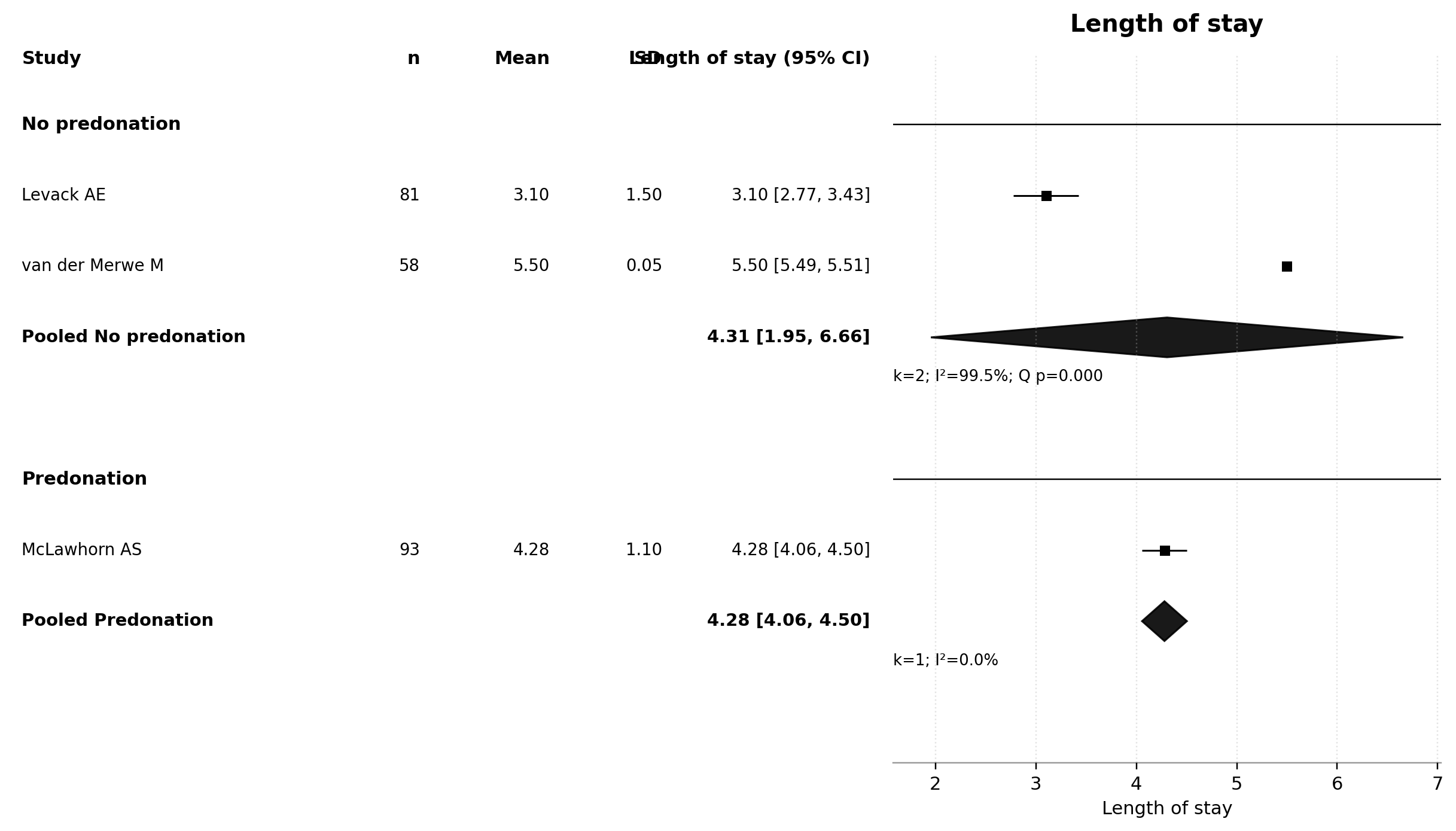

Supplement: Supplementary file 40 — Supplementary Figure 40. Forest plot: Autologous predonation – length of stay. Forest plot of the arm‐based multilevel random‐effects meta‐analysis comparing length of hospital stay between predonation and no‐predonation groups. No statistically significant difference was observed. Effect sizes are presented as mean differences with 95% confidence intervals. [file JEO2-13-e70867-s029.png]

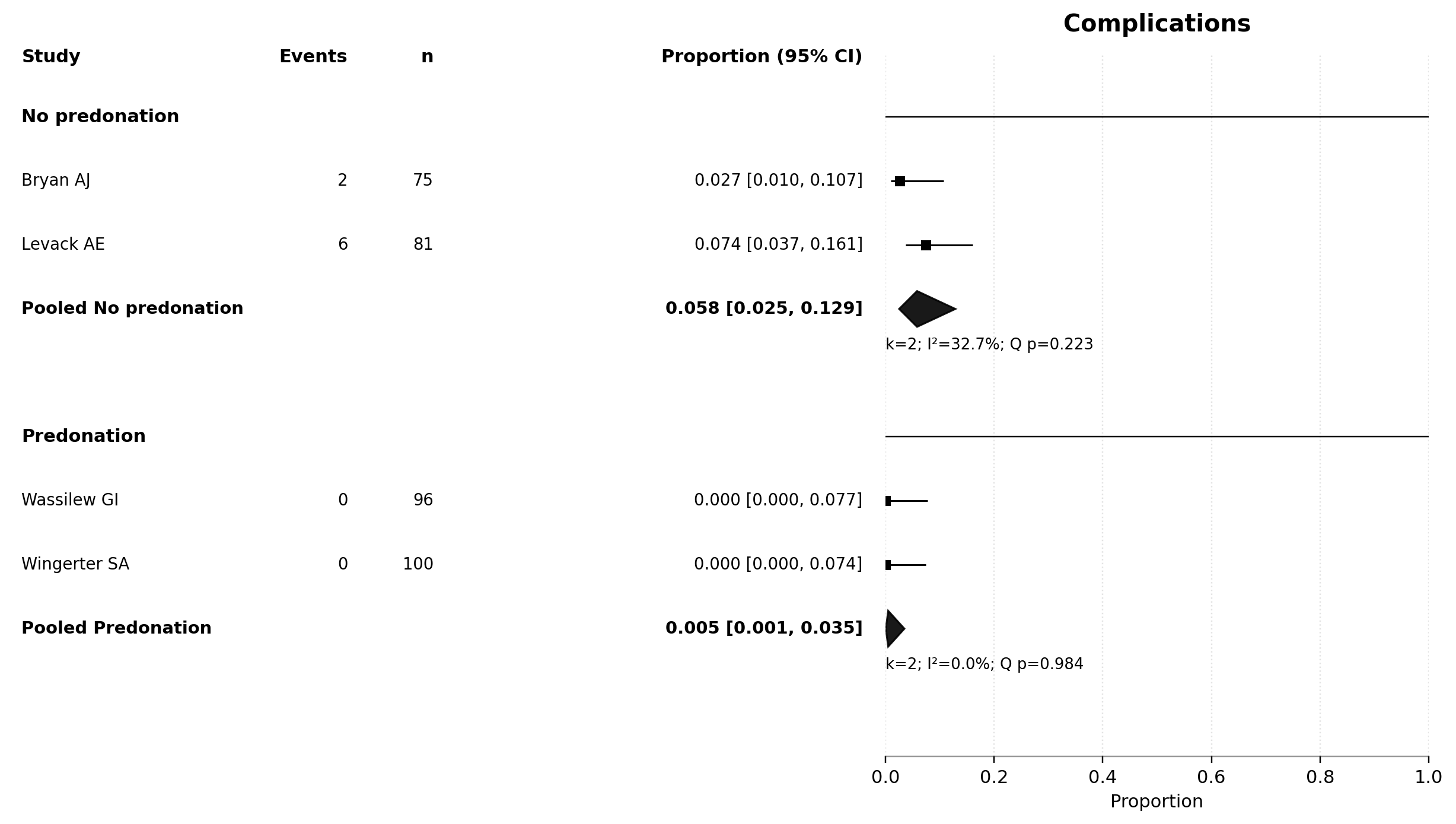

Supplement: Supplementary file 41 — Supplementary Figure 41. Forest plot: Autologous predonation – complications. Forest plot of the arm‐based multilevel random‐effects meta‐analysis comparing complication rates between predonation and no‐predonation groups. No statistically significant difference was observed. Effect sizes are presented as odds ratios with 95% confidence intervals. [file JEO2-13-e70867-s017.png]

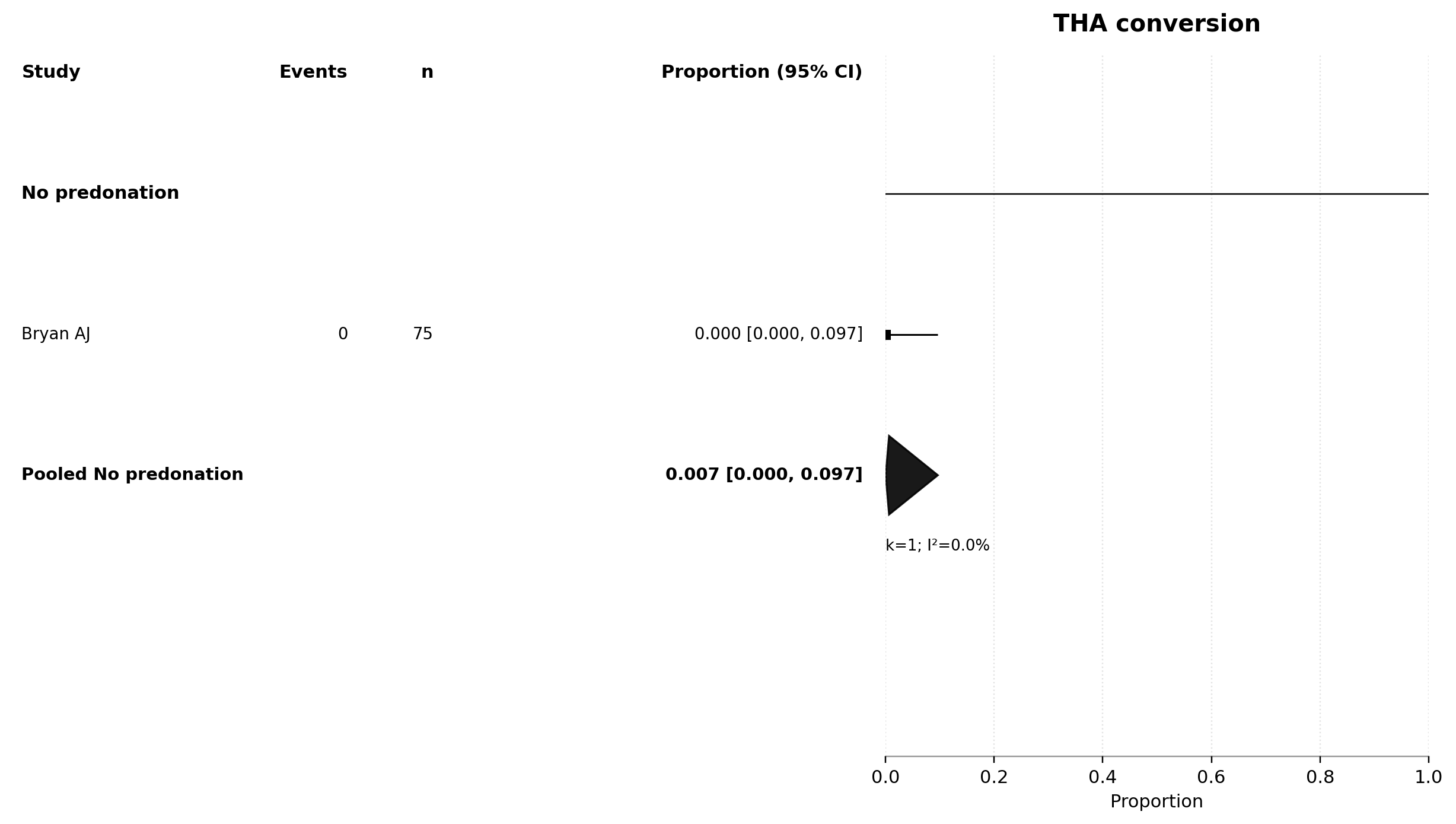

Supplement: Supplementary file 42 — Supplementary Figure 42. Forest plot: Autologous predonation – THA conversion. Forest plot of the arm‐based multilevel random‐effects meta‐analysis comparing conversion to total hip arthroplasty (THA) between predonation and no‐predonation groups. Data were insufficient for robust pooled analysis. [file JEO2-13-e70867-s034.png]
