# Supplementary material for: Association of tranexamic acid use and autologous predonation with blood loss and transfusion outcomes after periacetabular osteotomy: An arm‐based multilevel meta analysis
Source: J Exp Orthop. 2026 Jul 28;13(3):e70867. doi: 10.1002/jeo2.70867 (PMC13410948; doi:10.1002/jeo2.70867)
Supplement: Supplementary file 44 — Supplementary Table 2: Availability of standard deviation (SD) data for each included study. The table indicates whether SDs were directly reported or imputed according to the predefined imputation strategy described in the Methods. [file JEO2-13-e70867-s032.docx]

| Study | At least one reported SD | SD only imputed |
| --- | --- | --- |
| Albers 2013 |  | yes |
| Amano 2014 |  | yes |
| Atwal 2008 | yes |  |
| Bernstein 2007 |  | yes |
| Bryan 2016 |  | yes |
| Burke 2011 |  | yes |
| Clohisy 2005 |  | yes |
| Haertlé 2024 |  | yes |
| Khan 2017 |  | yes |
| Kim 2009 |  | yes |
| Kraeutler 2018 |  | yes |
| Lee 2013 |  |  |
| Lerch 2017 | yes |  |
| Levack 2020 | yes |  |
| Li 2022 |  | yes |
| Luo D 2016 | yes |  |
| Luo R 2021 | yes |  |
| Ma S 2022 | yes |  |
| Markhardt BK 2021 | yes |  |
| Marshall A 2025 |  | yes |
| McLawhorn AS 2016 | yes |  |
| Peters CL 2015 |  | yes |
| Peters CL 2006 | yes |  |
| Pogliacomi 2005 |  | yes |
| Pulido LF 2008 |  | yes |
| Sabbag CM 2019 | yes |  |
| Shang JJ 2020 | yes |  |
| Shon HC 2023 |  | yes |
| Siebenrock KA 1999 |  | yes |
| Sierra RJ 2017 |  | yes |
| Stambough JB 2014 |  | yes |
| Steppacher SD 2008 | yes |  |
| Tang Y 2022 |  | yes |
| Thawrani D 2010 | yes |  |
| Troelsen A et al. 2008 (1) |  | yes |
| Troelsen A et al. 2008 (2) |  | yes |
| van der Merwe M et al. 2019 |  | yes |
| Wassilew GI et al. 2015 |  | yes |
| Wingerter SA et al. 2015 |  | yes |
| Yilmaz M et al. 2022 |  | yes |
| Zaltz I et al. 2014 |  | yes |
| Zhu J et al. 2013 |  | yes |
| Ziran N et al. 2019 |  | yes |
